# Intestinal human carboxylesterase 2 (CES2) expression rescues drug metabolism and most metabolic syndrome phenotypes in global Ces2 cluster knockout mice

**DOI:** 10.1038/s41401-024-01407-4

**Published:** 2024-11-04

**Authors:** Yao-geng Wang, Chang-pei Gan, Joke Beukers-Korver, Hilde Rosing, Wen-long Li, Els Wagenaar, Maria C. Lebre, Ji-ying Song, Colin Pritchard, Rahmen Bin Ali, Ivo Huijbers, Jos H. Beijnen, Alfred H. Schinkel

**Affiliations:** 1 Division of Pharmacology, The Netherlands Cancer Institute, 1066 CX Amsterdam, The Netherlands; 2 Department of Pharmacy & Pharmacology, The Netherlands Cancer Institute, 1066 CX Amsterdam, The Netherlands; 3 Division of Experimental Animal Pathology, The Netherlands Cancer Institute, 1066 CX Amsterdam, the Netherlands; 4 Transgenic Core Facility, Mouse Clinic for Cancer and Aging (MCCA), The Netherlands Cancer Institute, 1066 CX Amsterdam, The Netherlands; 5 Faculty of Science, Department of Pharmaceutical Sciences, Division of Pharmacoepidemiology & Clinical Pharmacology, Utrecht University, 3584 CG Utrecht, The Netherlands

**Keywords:** metabolic syndrome, carboxylesterase 2, capecitabine, vinorelbine, lipid metabolism, glucose homeostasis

## Abstract

Carboxylesterase 2 (CES2) is expressed mainly in liver and intestine, but most abundantly in intestine. It hydrolyzes carboxylester, thioester, and amide bonds in many exogenous and endogenous compounds, including lipids. CES2 therefore not only plays an important role in the metabolism of many (pro-)drugs, toxins and pesticides, directly influencing pharmacology and toxicology in humans, but it is also involved in energy homeostasis, affecting lipid and glucose metabolism. In this study we investigated the pharmacological and physiological functions of CES2. We constructed *Ces2* cluster knockout mice lacking all eight *Ces2* genes (*Ces2*^*–/–*^ strain) as well as humanized hepatic or intestinal CES2 transgenic strains in this *Ces2*^*–/–*^ background. We showed that oral availability and tissue disposition of capecitabine were drastically increased in *Ces2*^*–/–*^ mice, and tissue-specifically decreased by intestinal and hepatic human CES2 (hCES2) activity. The metabolism of the chemotherapeutic agent vinorelbine was strongly reduced in *Ces2*^*–/–*^ mice, but only marginally rescued by hCES2 expression. On the other hand, *Ces2*^*–/–*^ mice exhibited fatty liver, adipositis, hypercholesterolemia and diminished glucose tolerance and insulin sensitivity, but without body mass changes. Paradoxically, hepatic hCES2 expression rescued these metabolic phenotypes but increased liver size, adipose tissue mass and overall body weight, suggesting a “healthy” obesity phenotype. In contrast, intestinal hCES2 expression efficiently rescued all phenotypes, and even improved some parameters, including body weight, relative to the wild-type baseline values. Our results suggest that the induction of intestinal hCES2 may combat most, if not all, of the adverse effects of metabolic syndrome. These CES2 mouse models will provide powerful preclinical tools to enhance drug development, increase physiological insights, and explore potential solutions for metabolic syndrome-associated disorders.

## Introduction

Mammalian carboxylesterases (EC 3.1.1.1) belong to a multigene superfamily encoding enzymes that have broad substrate specificity and catalyze the hydrolysis of ester, thioester and amide bonds [1–3]. Carboxylesterases (CESs) are enzymes involved in the detoxification and metabolism of (pro-)drugs and environmental toxicants [4–7]. However, carboxylesterases also hydrolyze endogenous esters and thioesters, including lipids, and some of these enzymes play important physiological functions in lipid metabolism and energy homeostasis [8]. Carboxylesterase genes encoding six human carboxylesterases (hCES1, hCES1P1, hCES2, hCES3, hCES4A and hCES5A) and twenty mouse carboxylesterases in five clusters (mCes1, mCes2, mCes3, mCes4 and mCes5) have been identified. Among these carboxylesterases, CES1 and CES2 are thought to be the most important members given their extensive functional implications. With respect to CES2, the mouse contains eight *Ces2* genes (*Ces2a* to *Ces2h*), including one pseudogene designated *Ces2d-ps*. All *Ces2* genes are located in a 286-kb gene cluster (*Ces2* cluster) on mouse chromosome 8. In contrast, humans have only one single *CES2* gene, which, together with *CES3* and *CES4A*, is located in a cluster on human chromosome 16 [9, 10]. Notably, unlike mCes1c, which is primarily secreted into plasma due to a lack of an ER retention signal, almost all other mouse and human CES proteins possess the HXEL ER retrieval sequence at their C-terminus (such as HIEL and HTEL for hCES1 and hCES2, respectively). This sequence is crucial for the localization of these enzymes to the ER lumen in mammalian cells [1, 8, 11]. However, as we previously reported that mCes2a may also be present in the blood [12] and as protein disulfide isomerases (ER retention signal-containing) are found at the cell surface [13], the ER retrieval sequence may not fully guarantee that proteins cannot be secreted into the blood.

Human CES2 (hCES2) is expressed mainly in the liver and intestine but is most abundantly expressed in the intestine and can hydrolyze carboxylesters as well as amide and thioester linkages in both exogenous and endogenous compounds. hCES1 and hCES2 share 47% amino acid identity and exhibit distinct but partly overlapping substrate specificities. hCES1 preferentially catalyzes the hydrolysis of compounds esterified with a small alcohol group, whereas hCES2 hydrolyzes compounds with a relatively small acyl group and large alcohol group [7]. hCES2 has broad substrate specificity, including the hydrolysis of narcotics (heroin, cocaine) [14], chemotherapy (pro-)drugs (irinotecan, capecitabine and gemcitabine) [15–18] and other drugs (procaine, prasugrel and flutamide) [19, 20].

Considering the high expression of CES2 in the intestine, it likely plays a role in the presystemic elimination of substrate drugs following oral administration (first-pass metabolism). The CES2 present inside enterocytes can metabolize (part of) substrate drugs before they reach the liver, where they are subsequently further hydrolyzed by hepatocyte CES2. This combined first-pass metabolism may thus strongly influence overall systemic exposure and the efficacy of specific substrate drugs, as is the case for CYP3A-metabolized drugs. On the other hand, for CES-activated prodrugs, high CES2 levels in enterocytes may cause more active drug or metabolite accumulation in the intestinal tissue and thus local toxicity, such as chemotherapy-induced diarrhea (CID) [21]. CES2 is also expressed in various tumors, including hepatocellular carcinoma, esophageal squamous carcinoma, colon adenocarcinoma, and renal adenocarcinoma. However, in most of these tumors, CES2 expression is significantly lower than that in the corresponding normal tissues. CES2 expression also varies among different cancer types and individuals, so differences in CES2 activity in tumors may influence the response of tumor tissue to specific drugs [22]. In addition, systemic exposure changes may affect antitumor chemotherapies, such as irinotecan and gemcitabine [16, 23]. In summary, CES2 plays an important role in the metabolism of many exogenous compounds, such as (pro-)drugs, toxins and pesticides, which directly influences pharmacology and toxicology in humans.

As CES2 can further hydrolyze endogenous lipids, such as triglycerides, cholesteryl esters and retinyl esters, it is also involved in energy homeostasis, affecting lipid and glucose metabolism [8]. Li et al. demonstrated that hepatic CES2 plays an important role in controlling hepatic triglyceride homeostasis by regulating lipolysis, fatty acid oxidation (FAO), ER stress, and lipogenesis to alleviate liver steatosis and in improving glucose tolerance and energy expenditure. This process appears to be regulated through hepatic HNF-4a [24]. Similarly, another study suggested that CES2 promotes lipid oxidation to reverse hepatic steatosis and glucose intolerance [25]. A recent study further revealed that mCes2a knockout reduced diacylglycerol (DAG) and lysophosphatidylcholine (lysoPC) hydrolysis in the liver, caused obesity and fatty liver and disrupted glucose homeostasis via lipid signaling [26]. In addition to hepatic CES2, intestinal mCes2c overexpression protected mice from excessive diet-induced weight gain and liver steatosis [27]. These studies reveal the important functions of CES2 in energy homeostasis, including lipid metabolism and glucose handling, and a potential role of CES2 in protection from metabolic syndrome. However, compared with more substantial physiological studies of CES1 functions, such physiological research on CES2 remains limited.

The bifunctionality of CES2 in the metabolism of both xenobiotics (e.g., drugs) and various lipids, thus affecting pharmacology and toxicology on the one hand and lipid and glucose homeostasis on the other hand, may seem puzzling at first. However, given that excess lipids can act as lipotoxins that damage cells, organs, and ultimately the whole organism, it is clear that CES2 functions as a detoxification mechanism for lipids, similar to its role in detoxifying xenobiotic toxins. In that sense, there is no contradiction between the two functions of CES2 in limiting toxic damage due to xenobiotics such as drugs on the one hand and excess lipids on the other hand.

To better understand the pharmacological and physiological functions of CES2, we generated and characterized *Ces2* cluster knockout (*Ces2*^*–/–*^) mice. Considering the high intestinal and significant (albeit variable) hepatic CES2 expression in humans, we also generated and characterized homozygous transgenic mice with stable and abundant expression of human *CES2* cDNA in either the liver or intestine of *Ces2* cluster knockout mice (*Ces2*^*–/–*^A or *Ces2*^*–/–*^V mice, respectively). By analyzing the pharmacokinetics and metabolism of administered chemotherapy drugs (capecitabine and vinorelbine), we gained deeper insights into the pharmacological functions of CES2. This understanding can enhance our knowledge of the in vivo drug handling process and potentially improve drug administration regimens. In addition, a better understanding of the lipid and glucose homeostasis processes in which CES2 may be involved could provide clues on how to ameliorate aspects of metabolic syndrome.

## Materials and methods

### Materials

Capecitabine was purchased from Carbosynth (Berkshire, UK). Vinorelbine (GlaxoSmithKline) was obtained from the pharmacy of The Netherlands Cancer Institute. The Lipid Extraction Kit was obtained from PromoCell GmbH (Heidelberg, Germany). The LabAssay Triglyceride Kit was from Wako Chemicals (Tokyo, Japan). Isoflurane was purchased from Pharmachemie (Haarlem, The Netherlands), heparin (5000 IU/mL) was from Leo Pharma (Breda, The Netherlands). All other chemicals and reagents were obtained from Sigma-Aldrich (Steinheim, Germany).

### Animals

Mice were housed and handled according to institutional guidelines complying with Dutch and EU legislation. All mouse strains were generated and maintained at the Netherlands Cancer Institute. All experimental animal protocols were evaluated and approved by the institutional animal care and use committee. Wild-type, *Ces2* cluster knockout mice (*Ces2*^*–/–*^), and CES2-humanized *Ces2*^*–/–*^ mice, with abundant expression of hCES2 in liver (*Ces2*^*–/–*^A) or intestine (*Ces2*^*–/–*^V), respectively, were all of a > 99% FVB genetic background. Mice between 9 and 16 weeks of age were used for pharmacokinetic studies, between 4 and 20 weeks of age for body weight monitoring and basic physiology studies (hematology, plasma chemistry, histology/pathology, etc.), between 12 and 16 weeks old for VLDL (Very Low-Density Lipoprotein) production, lipid tolerance test, glucose tolerance test and insulin resistance test, and aging mice of ~60 weeks old were used for basic physiology analyses. All the animals were kept in a temperature-controlled environment with 12-h light and 12-h dark cycle and they received a standard medium-fat diet (Transbreed, SDS Diets, Technilab-BMI, fat content 10% by weight, 24% by calories, Someren, The Netherlands) and acidified water *ad libitum*.

### Generation of *Ces2*^*–/–*^, *Ces2*^*–/–*^A and *Ces2*^*–/–*^V mice

Details of the development of *Ces2*^*–/–*^, *Ces2*^*–/–*^A and *Ces2*^*–/–*^V mice are described in Supplementary Methods. Briefly, *Ces2*^*–/–*^ were generated using CRISPR-Cas9 technology, and transgenic *Ces2*^*–/–*^A and *Ces2*^*–/–*^V were generated using two tissue-targeting human *CES2* cDNA expression plasmids with ApoE (primarily liver) and Villin (primarily intestinal) promoters, respectively.

### Real-time PCR analysis

RNA was isolated by RNeasy Mini Kit (Qiagen, Hilden, Germany) from mouse liver and small intestine (SI). Subsequently cDNA synthesis was done by Maxima First Strand cDNA Synthesis Kit (Thermo Scientific, Waltham, MA, USA), and real-time (RT)-PCR using specific primers (Qiagen, Hilden, Germany) for mouse *Ces1a*, *Ces1b*, *Ces1c*, *Ces1d*, *Ces1e*, *Ces1f*, *Ces1g*, *Ces1h*, *Ces2a*, *Ces2b*, *Ces2c*, *Ces2e*, *Ces2f*, *Ces2g*, *Ces2h*, *Ces3a* and *Ces3b* was performed as described previously [28].

### Western blot and immunohistochemical analysis

Crude total cellular membrane fractions were isolated from mouse liver, kidney, and small intestine as described previously [29]. Protein concentration was quantified by the BCA protein Assay Kit (Thermo Scientific, Waltham, MA, USA). After size separation and transfer, proteins were probed with rabbit anti-human CES2 monoclonal antibody (ab184957, Abcam, Cambridge, UK) (diluted 1:2000) or rabbit anti-β-actin monoclonal antibody (#4970, Cell Signaling Technology, Danvers, MA, USA) (dilution 1:2,000), followed by HRP-labeled goat anti-rabbit second antibody (diluted 1:5000) (Agilent Dako, Santa Clara, CA, USA). Immunohistochemistry on wild-type, *Ces2*^*–/–*^, *Ces2*^*–/–*^A and *Ces2*^*–/–*^V tissues was conducted with the same rabbit anti-human CES2 monoclonal antibody (ab184957), and secondary antibody conjugated to HRP-labeled polymers (EnVision+ System-HRP; Agilent Dako, Santa Clara, CA, USA).

### Body weight monitoring, histology/pathology, plasma clinical chemistry and hematology analysis

The body weights of mice (~20 female and ~20 male mice from each mouse strain) were monitored every week from 4 weeks to 20 weeks old. At week 20, mice were fasted overnight (16 h) and sacrificed for blood and organ collection. Isolated tissues were handled as described previously for Hematoxylin and Eosin (H&E)-staining, and Oil Red O-staining [30]. Parts of liver samples were snap-frozen in dry ice for further lipid content analysis. The semi-quantitative assessment standard for H&E-staining of white adipose tissue (WAT) and Oil Red O-staining of liver can be found in Supplementary Methods. Standard clinical-chemistry analyses on plasma were performed on a Roche Hitachi 917 analyzer to determine levels of alkaline phosphatase, alanine aminotransaminase, Na^+^, K^+^, Ca^2+^, Cl^−^, urea, uric acid, glucose, triglycerides and cholesterol. Hemoglobin, hematocrit, mean corpuscular volume, red and white blood cell counts, and platelets were analyzed in peripheral blood on a Cell Dyn 1200 analyzer (Abbott, Chicago, IL, USA). The aging mice at ~60 weeks (3–6 mice, either female or male) were sacrificed for histology, plasma clinical-chemistry and hematology analyses as well. Moreover, 12-13 weeks old young adult mice ( ~ 10 females and ~10 males from each strain) were sacrificed and different tissues, including organs and adipose tissues, were collected for absolute weight and tissue-to-body weight ratio analysis.

### Drug solutions

Capecitabine was first dissolved in dimethyl sulfoxide (DMSO) at a concentration of 500 mg/mL and further diluted with mixed buffer (polysorbate 20 : absolute ethanol = 1 : 1, *v/v*) and 40 mM Na-Acetate (NaAc, pH 4.2), to make up the final working solution of 50 mg/mL in [DMSO : Polysorbate 20 : absolute ethanol : 40 mM NaAc (pH 4.2) = 10 : 15 : 15 : 60, (*v/v/v/v*)]. Vinorelbine (10 mg/mL) was diluted with normal saline fivefold to 2 mg/mL for intravenous injection and tenfold to 1 mg/mL for oral administration. All dosing solutions were prepared freshly on the day of experiment. Although ethanol can somewhat inhibit CES1, in a preceding study we demonstrated a marked in vivo contribution of both mCes1 and hepatic transgenic hCES1 to capecitabine hydrolysis under the same experimental conditions [31].

### Plasma and organ pharmacokinetics of capecitabine and vinorelbine in mice

In order to minimize variation among individuals, mice were fasted for 3 h before drug was administered orally or intravenously. For capecitabine 2-h experiments, 6–7 female mice received oral capecitabine (500 mg/kg, 10 μL/g), and tail vein blood samples were collected at 0.125, 0.25, 0.5 and 1 h after drug administration. For the vinorelbine 4-h experiment, 6-7 male mice received vinorelbine intravenously (10 mg/kg, 5 μL/g) or orally (10 mg/kg, 10 μL/g) using a blunt-ended needle. Tail vein blood samples were collected at 0.125, 0.25, 0.5, 1 and 2 h after drug administration. Time points of termination were chosen to allow both reasonable assessment of the plasma exposure of the compound and its main metabolites, and adequate measurement of tissue concentrations. In order to minimize the generation and use of mice, and as no pronounced sex differences were expected, female mice were used for the capecitabine studies and male mice for the vinorelbine studies. Tail vein blood sample collection (~50 μL) was performed using microvettes containing dipotassium-EDTA. All blood samples were rapidly placed on ice to limit any ongoing hydrolysis. At the last time point in each experiment (2 or 4 h), mice were anesthetized with 5% isoflurane and blood was collected by cardiac puncture. Cardiac puncture blood samples (600–800 μL) were collected in Eppendorf tubes containing heparin as an anticoagulant. The mice were then sacrificed by cervical dislocation and brain, liver, kidney, lung, small intestine and testis were rapidly removed. Plasma was isolated from the blood by centrifugation at 9000 × *g* for 6 min at 4 °C, and the plasma fraction was collected and stored at −30 °C until analysis. Organs were homogenized with 4% (w/v) bovine serum albumin and stored at −30 °C until analysis. Relative metabolite-to-parental drug ratio after drug administration was calculated by determining metabolite tissue concentration relative to corresponding parental drug concentration at the last time point.

### HPLC-MS analysis

Concentrations of capecitabine (and its metabolites) and vinorelbine (and its metabolite) in mouse plasma samples and organ homogenates were determined using two independent validated high-performance liquid chromatography-tandem mass spectrometry assays [32, 33].

### VLDL secretion and lipid tolerance tests

For the VLDL secretion experiment, mice were fasted for 16 h and then Poloxamer-407 (1 g/kg) was injected intraperitoneally. Blood samples were collected from the tail vein before injection (0 h) and 1, 2, 3, and 4 h after injection of the lipase inhibitor. For the lipid tolerance test, mice were fasted for 16 h and olive oil (10 μL/g) was then orally administered by a blunt-ended needle. Blood samples were collected from the tail vein at different time points as described above. All the plasma samples were processed and triglyceride levels were analyzed with the LabAssay Triglyceride Kit.

### Glucose and insulin tolerance tests

For the glucose tolerance test, mice fasted for 16 h received orally administered glucose (1 g/kg). Blood glucose levels in tail vein samples were monitored at baseline (0 min, before glucose was administered) and various time points (15, 30, 60, 90, and 120 min) after glucose was administered with an ACCU-CHEK Performa glucose meter (Roche). For the insulin tolerance test, mice were first fasted for 6 h and then received an intraperitoneal injection with insulin (0.5 U/kg). Blood glucose levels were measured at different time points as described above.

### Data and statistical analysis

Pharmacokinetic parameters were calculated by non-compartmental methods using the PK solver software [34]. The area under the plasma concentration-time curve (AUC) was calculated using the trapezoidal rule, without extrapolating to infinity. The peak plasma concentration (*C*_max_) and the time of maximum plasma concentration (*T*_max_) were estimated from the original (individual mouse) data. One-way analysis of variance (ANOVA) was used when multiple groups were compared and the Tukey’s test *post hoc* correction was used to accommodate multiple testing. The two-sided unpaired Student’s *t*-test was used when treatments or differences between two specific groups were compared. Kruskal-Wallis rank test was used for semi-quantitative assessment for (H&E)-staining of white adipose tissue and Oil Red O-staining of liver. All statistical analyses were performed using the software GraphPad Prism 8 (GraphPad Software Inc., La Jolla, CA, USA). All the linear data were log-transformed before statistical tests were applied. Differences were considered statistically significant when *P* < 0.05. All data are presented as mean ± SD.

## Results

### Generation of *Ces2* cluster knockout mice

**Fig. 1:**
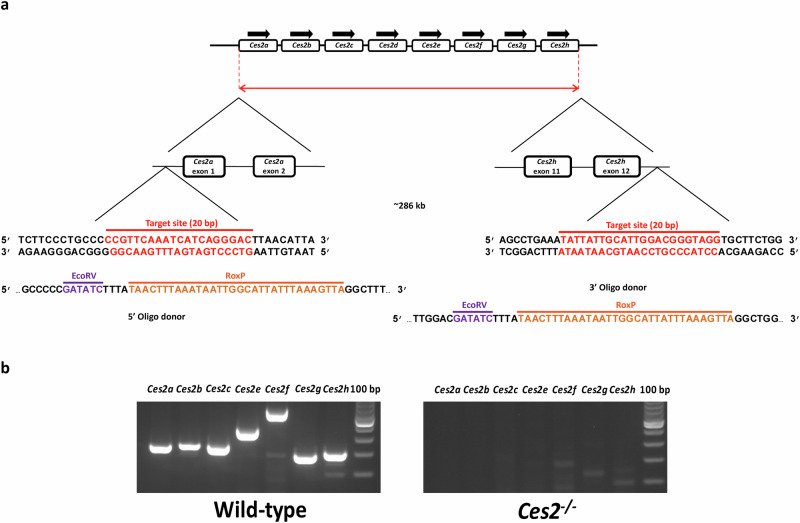
Ces2 knockout mouse model generation and characterization. **a** Schematic overview of the CRISPR-Cas9 strategy for deletion of the Ces2 cluster genes and (**b**) PCR analysis for all known functional *Ces2* genes in tail DNA of WT and *Ces2*^*−/−*^ mice.

In mice, 7 full-length *Ces2* genes (*Ces2a-2c*, *Ces2e-2h*) and 1 pseudogene (*Ces2d*) have been identified [10]. We originally aimed to obtain a conditional whole *Ces2* cluster (from *Ces2a* to *Ces2h*) deletion via the insertion of 5’ and 3’ RoxP recombination sites flanking the *Ces2* locus using the CRISPR/Cas9 method in fertilized oocytes (Fig. 1a). Offspring with RoxP sites flanking the whole *Ces2* cluster could then be further crossbred with Dre-expressing mice to generate a conditional *Ces2* knockout mouse strain [35]. In our first attempts, we obtained only one candidate line with a RoxP site located upstream of *Ces2a* but not downstream of *Ces2h*. However, PCR amplification and DNA sequencing of offspring revealed that a full *Ces2* cluster deletion resulting from direct ligation of the two CRISPR/Cas9 cutting sites without RoxP insertion was also obtained. This complete *Ces2* cluster deletion allele was then backcrossed to the wild-type (FVB/NRj) background for at least three generations to dilute any potential off-target mutations. Homozygotes of the complete *Ces2* cluster deletion (*Ces2*^*–/–*^) were then generated by crossbreeding heterozygous knockout mice. *Ces2*^*–/–*^ mice were viable, fertile and without prominent anatomic alterations, removing the need for further generation of conditional *Ces2* knockout lines.

Deletion of the *Ces2* cluster genes was verified by comparing wild-type and *Ces2*^*–/–*^ mice using PCR to assess the presence of each *Ces2* gene (except for the pseudogene *Ces2d*) (primers in Supplementary Table S1). PCR products of all functional *Ces2* genes (*Ces2a-2c* and *Ces2e-2h*) were present in WT mice but absent in *Ces2*^*-/-*^ mice (Fig. 1b). In addition, quantitative real-time PCR (qRT‒PCR) analysis was performed for all functional *Ces1*, *Ces2* and *Ces3* genes in the liver and small intestine of both wild-type and *Ces2*^*–/–*^ mice. The results revealed that in wild-type mice, m*Ces1* genes, especially *Ces1b-1g*, were highly expressed in the liver, whereas Ces2 genes, such as *Ces2a-2e*, were highly expressed in the small intestine. Notably, *Ces2a-2e* were also expressed quite abundantly in the liver, although their expression was not as high as that of some *Ces1* genes (Supplementary Table S2). Compared with those in WT mice, all the functional *Ces2* mRNA signals were dramatically decreased in the liver and small intestine of *Ces2*^*–/–*^ mice, whereas no significant changes in *Ces1* gene expression were observed (Supplementary Table S2 and Supplementary Fig. S1). There was further a significant decrease in the *Ces3a* and *Ces3b* levels in the small intestine, although this decrease occurred from an already very low basal expression level.

### Humanized mice with stable transgenic human CES2 expression in the liver or intestine in a *Ces2* knockout background

**Fig. 2:**
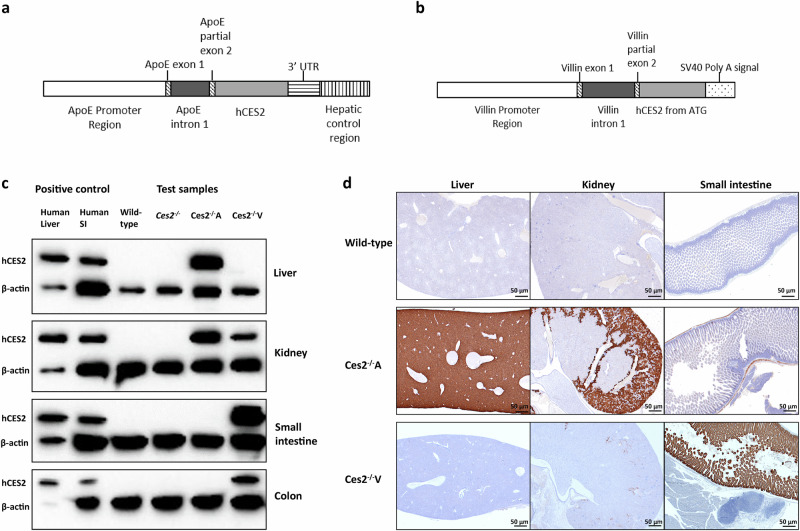
Generation and characterization of human CES2 liver- or intestine-specific transgene expressing mouse models. **a** Schematic structure of ApoE promoter-HCR1-driven expression cassette containing human CES2 cDNA; (**b**) schematic structure of Villin promoter-driven expression cassette containing human CES2 cDNA; (**c**) western blot analysis of crude membrane fractions of liver, kidney, small intestine (SI) and colon from wild-type, *Ces2*^*–/–*^, human CES2 liver transgenic (*Ces2*^*–/–*^A) and human CES2 intestine transgenic (*Ces2*^*–/–*^V) mice; human liver and human small intestinal (SI) lysates were used as positive controls and for comparison on the Western blots; (**d**) immunohistochemical staining of human CES2 in liver, small intestine and kidney of wild-type, *Ces2*^*–/–*^A and *Ces2*^*–/–*^V mice.

Considering the substantial expression of CES2 in the human liver and intestine, homozygous transgenic mice containing human *CES2* cDNA expressed primarily in the liver or intestine in a mouse *Ces2* cluster deletion background were generated via zygote injection of two expression cassettes (ApoE-hCES2-HCR-driven liver-targeting cassette and villin-hCES2-SV40-driven intestine-targeting cassette, with the hepatic control region (HCR) in the ApoE-hCES2-HCR cassette further boosting liver expression, Fig. 2a, b). The cross-breeding strategy from heterozygous to stable homozygous *hCES2* transgenic and *Ces2* knockout mice is described in the Supplementary Methods. We hereafter refer to these strains as *Ces2*^*–/–*^A and *Ces2*^*–/–*^V, respectively.

Crude membrane fractions from the liver, kidney, small intestine and colon of wild-type, *Ces2*^*–/–*^, *Ces2*^*–/–*^A, and *Ces2*^*–/–*^V mice, together with hCES2-expressing human donor tissues as positive controls, were analyzed for hCES2 expression via Western blotting with a specific rabbit anti-human CES2 monoclonal antibody. As expected, hCES2 was not detected in any of the tissues collected from wild-type or *Ces2*^*–/–*^ mice. hCES2 in *Ces2*^*–/–*^A mice was expressed in the liver but not in the intestine, whereas hCES2 was highly expressed in the small intestine and at relatively lower levels in the colon but not in the liver in *Ces2*^*–/–*^V mice (Fig. 2c). However, some hCES2 expression in *Ces2*^*–/–*^A and *Ces2*^*–/–*^V mice was also detected in the kidney, a site of expression that was also reported in a previous study of these expression cassettes [30].

To further identify the location of hCES2 expression, immunohistochemical staining was performed with the same antibody. The results revealed strong hCES2 expression in nearly all hepatocytes, some expression in renal convoluted ducts and slight expression in the muscularis layer of the intestine in *Ces2*^*–/–*^A mice (Fig. 2d). In contrast, in *Ces2*^*–/–*^V mice, hCES2 was highly expressed in all enterocytes, slightly expressed in renal convoluted ducts, and undetectable in the liver parenchyma. This finding is consistent with the Western blot results. A similar localization and expression profile was previously observed for human CYP3A4 in analogous transgenic mouse models [30]. Thus, two transgenic mouse strains expressing hCES2 predominantly in the liver or intestine were obtained. hCES2 expression was rechecked using Western blot after 9 generations of breeding and was found to be stable. Similar as noted for the *Ces2*^*–/–*^ mice, both the *Ces2*^*–/–*^A and *Ces2*^*–/–*^V mouse strains were viable and fertile, with normal lifespans and no prominent anatomic alterations.

### Mouse Ces2 and human CES2 influence capecitabine metabolism, affecting plasma and tissue disposition: validation in female mice

Capecitabine, an oral anticancer prodrug, is biotransformed into active 5-fluorouracil (5-FU) in three steps, and carboxylesterases are believed to be involved in the first metabolic step from capecitabine to 5’-DFCR [36] (Supplementary Fig. S2). In vitro, both hCES1 and hCES2 hydrolyze the carbamate bond in capecitabine to yield its first metabolite, 5’-DFCR, with roughly equal efficiency [18]. We previously demonstrated that mCes1 and, to a lesser extent, liver transgenic hCES1 play a marked role in the in vivo conversion of capecitabine to 5’-DFCR [31]. To study the impact of CES2 on capecitabine metabolism and disposition in vivo, 500 mg/kg capecitabine was orally administered to female wild-type, *Ces2*^*–/–*^, *Ces2*^*–/–*^A and *Ces2*^*–/–*^V mice, and plasma and tissue concentrations of capecitabine and its four metabolites (5’-DFCR, 5’-DFUR, 5-FU and FBAL) were measured [33]. As there was no sex preference, this experiment was performed using females on the basis of mouse availability.

**Fig. 3:**
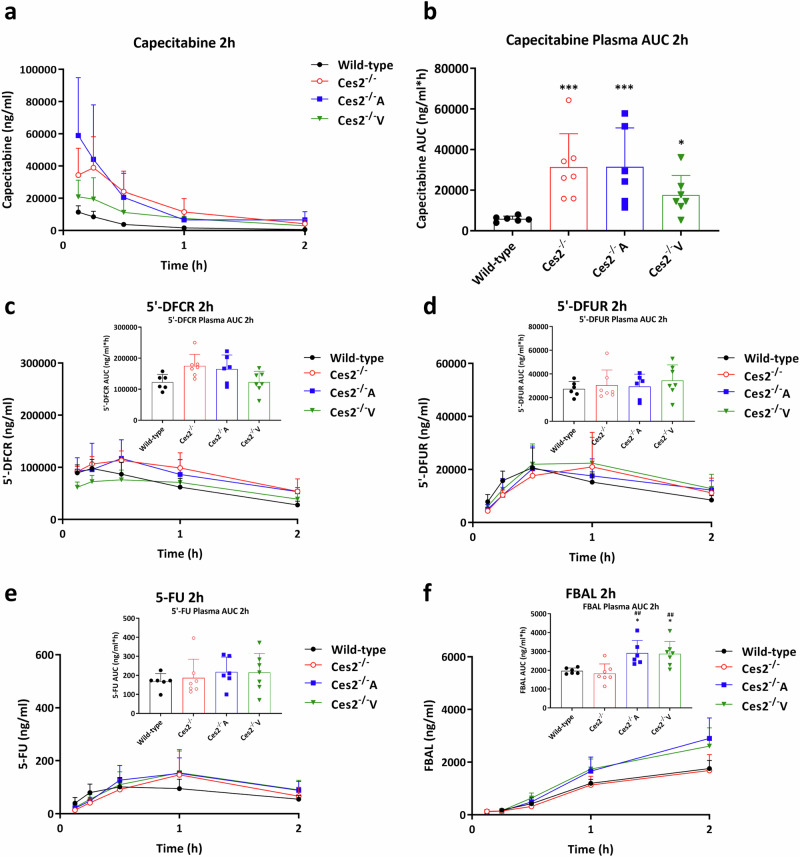
Capecitabine plasma pharmacokinetic results in CES2-modified mouse models. Plasma concentration-time curves (**a**) and AUC_0-2h_ (**b**) of capecitabine and corresponding results of metabolites 5’-DFCR (**c**), 5’-DFUR (**d**), 5-FU (**e**) and FBAL (**f**) in female wild-type, *Ces2*^*–/–*^, *Ces2*^*–/–*^A and *Ces2*^*–/–*^V mice over 2 h after oral administration of 500 mg/kg capecitabine. Data are given as mean ± SD (*n* = 5–7). *, *P* < 0.05; ***, *P* < 0.001 compared to wild-type mice. ^##^, *P* < 0.01; compared to *Ces2*^*–/–*^ mice. No statistical differences were found between *Ces2*^*–/–*^A and *Ces2*^*–/–*^V mice. Statistical analysis was applied after log-transformation of linear data.

We observed a 5.3-fold greater plasma AUC_0-2h_ of capecitabine in *Ces2*^*–/–*^ mice than in wild-type mice (*P* < 0.001, Fig. 3a, b). The plasma AUC_0-2h_ of capecitabine in *Ces2*^*–/–*^A mice was similar to that in *Ces2*^*–/–*^ mice, whereas the value was 44% lower in *Ces2*^*–/–*^ V mice (Table 1). Surprisingly, the absolute values of the plasma AUC_0-2h_ for the first 3 metabolites (5’-DFCR, 5’-DFUR and 5-FU) were similar among these four strains, with only FBAL showing greater plasma exposure in *Ces2*^*–/–*^A and *Ces2*^*–/–*^V mice (Fig. 3c–f and Table 1). Nevertheless, owing to the differences in capecitabine plasma exposure (Fig. 3a), the dynamic conversion from capecitabine to metabolites, as calculated based on the metabolite-to-capecitabine ratios in plasma, was clearly impacted by mCes2 and hCES2, especially in *Ces2*^*–/–*^V mice (Table 1).

**Table 1:** Plasma pharmacokinetic parameters of capecitabine and its metabolites 5’-DFCR, 5’-DFUR, 5-FU and FBAL in female wild-type, *Ces2*^*–/–*^*, Ces2*^*–/–*^A and *Ces2*^*–/–*^V mice over 2 h after oral administration of 500 mg/kg capecitabine.

Capecitabine parameter	Genotype/Groups			
	Wild-type	*Ces2*^*–/–*^	*Ces2*^*–/–*^A	*Ces2*^*–/–*^V
AUC_0-2h_, ng/mL*h	5846 ± 1375	31267 ± 16563***	31533 ± 19150***	17535 ± 9707*
*C*_max_, ng/mL	12192 ± 3796	42600 ± 17764**	47033 ± 34131*	23369 ± 14079
*T*_max_, h	0.19 ± 0.07	0.21 ± 0.06	0.17 ± 0.07	0.16 ± 0.06

5’-DFCR parameter	Genotype/Groups			
	Wild-type	*Ces2*^*–/–*^	*Ces2*^*–/–*^A	*Ces2*^*–/–*^V
AUC_0-2h_, ng/mL*h	122720 ± 24948	174979 ± 37341	164965 ± 45226	122870 ± 34694
AUC_0-2h_ ratio (5’-DFCR/capecitabine)	21.3 ± 3.5	6.4 ± 1.9***	6.4 ± 2.4***	8.0 ± 2.2***
*C*_max_, ng/mL	101350 ± 18009	120557 ± 23850	117133 ± 35728	80014 ± 18212^#^
*T*_max_, h	0.25 ± 0.14	0.46 ± 0.27	0.46 ± 0.10	0.61 ± 0.38

5’-DFUR parameter	Genotype/Groups			
	Wild-type	*Ces2*^*–/–*^	*Ces2*^*–/–*^A	*Ces2*^*–/–*^V
AUC_0-2h_, ng/mL*h	27337 ± 6291	30434 ± 12950	29452 ± 10370	34477 ± 13143
AUC_0-2h_ ratio (5’-DFUR/capecitabine)	4.7 ± 0.9	1.0 ± 0.9***	1.1 ± 0.3***	2.1 ± 0.4***^###^^^^
*C*_max_, ng/mL	21133 ± 7207	22886 ± 11936	21100 ± 8366	23871 ± 8673
*T*_max_, h	0.42 ± 0.13	0.64 ± 0.24	0.58 ± 0.20	0.79 ± 0.27*

5-FU parameter	Genotype/Groups			
	Wild-type	*Ces2*^*–/–*^	*Ces2*^*–/–*^A	*Ces2*^*–/–*^V
AUC_0-2h_, ng/mL*h	168 ± 41	187 ± 98	218 ± 79	215 ± 100
AUC_0-2h_ ratio (×10^−3^) (5-FU/capecitabine)	29 ± 8.1	6.4 ± 2.1***	8.0 ± 2.7***	13 ± 1.6***^###^^
*C*_max_, ng/mL	105 ± 36	147 ± 96	159 ± 56	156 ± 81
*T*_max_, h	0.50 ± 0.27	0.93 ± 0.19**	0.92 ± 0.20*	0.86 ± 0.24*

FBAL parameter	Genotype/Groups			
	Wild-type	*Ces2*^*–/–*^	*Ces2*^*–/–*^A	*Ces2*^*–/–*^V
AUC_0-2h_, ng/mL*h	1966 ± 158	1833 ± 504	2910 ± 671*^##^	2874 ± 659*^##^
AUC_0-2h_ ratio (FBAL/capecitabine)	0.35 ± 0.08	0.07 ± 0.04***	0.12 ± 0.06**	0.20 ± 0.09^##^
*C*_max_, ng/mL	1755 ± 308	1684 ± 597	2923 ± 760*^##^	2606 ± 698^#^
*T*_max_, h	2.0 ± 0.0	2.0 ± 0.0	1.8 ± 0.4	2.0 ± 0.0

Data are given as mean ± SD (*n* = 6). AUC_0-2h_, area under plasma concentration-time curve; *C*_max_, maximum concentration in plasma; *T*_max_, time point (h) of maximum plasma concentration; *, *P* < 0.05; **, *P* < 0.01; ***, *P* < 0.001 compared to wild-type mice. ^#^, *P* < 0.05; ^##^, *P* < 0.01; ^###^, *P* < 0.001 compared to *Ces2*^*–/–*^mice. No statistical difference was found between *Ces2*^*–/–*^A and *Ces2*^*–/–*^V mice. Statistical analysis was applied after log-transformation of linear data.

**Fig. 4:**
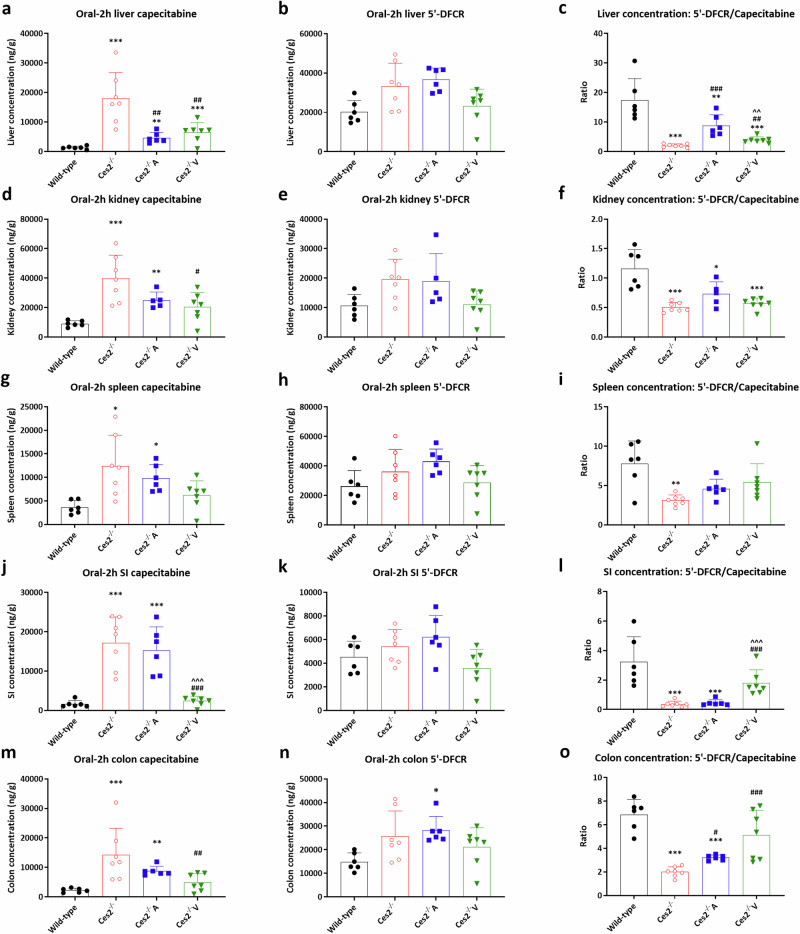
Tissue distribution results of capecitabine and its first metabolite 5’-DFCR. Liver, kidney, spleen, small intestine (SI) and colon concentrations of capecitabine (**a**, **d**, **g**, **j** and **m**) and 5’-DFCR (**b**, **e**, **h**, **k** and **n**), and capecitabine to 5’DFCR conversion ratios in above mentioned tissues (**c**, **f**, **i**, **l** and **o**) in female wild-type, *Ces2*^*–/–*^, *Ces2*^*–/–*^A and *Ces2*^*–/–*^V mice over 2 h after oral administration of 500 mg/kg capecitabine. Data are given as mean ± SD (*n* = 5–7). *, *P* < 0.05; **, *P* < 0.01; ***, *P* < 0.001 compared to wild-type mice. ^#^, *P* < 0.05; ^##^, *P* < 0.01; ^###^, *P* < 0.001 compared to *Ces2*^*–/–*^ mice. ^^, *P* < 0.01; ^^^, *P* < 0.001 for comparison between *Ces2*^*–/–*^A and *Ces2*^*–/–*^V mice. Statistical analysis was applied after log-transformation of linear data.

The liver, kidney, spleen, lung, SI, SIC and colon were collected 2 h after drug administration, and the absolute drug concentrations and tissue-to-plasma ratios were analyzed. In the liver, the capecitabine concentration was 13.8-fold greater in *Ces2*^*–/–*^ mice than in wild-type mice (*P* < 0.001). Compared with that in *Ces2*^*–/–*^ mice, the capecitabine liver concentration in *Ces2*^*–/–*^A mice and *Ces2*^*–/–*^V mice was reduced by hCES2 activity by 74.2% and 63.5%, respectively (Fig. 4a and Supplementary Table S3). Compared with that noted in *Ces2*^*–/–*^ mice, the capecitabine liver-to-plasma ratios were also decreased due to the expression of hCES2, especially in *Ces2*^*–/–*^A mice (Supplementary Fig. 3a, b). In small intestine, the absolute capecitabine concentration was again dramatically higher in *Ces2*^*-/-*^ mice than in wild-type mice (10.6-fold, *P* < 0.001), whereas *Ces2*^*–/–*^V mice showed profoundly reduced capecitabine concentrations from 17,217 ± 6548 ng/g to 2411 ± 1191 ng/g (*P* < 0.001), reverting to the same level as seen in wild-type mice (Fig. 4j and Supplementary Table S3). In contrast, *Ces2*^*–/–*^A mice still had intestinal concentrations similar to those of *Ces2*^*–/–*^ mice. For the 3 first metabolites (5’-DFCR, 5’-DFUR and 5-FU), the absolute tissue concentrations were mostly similar among the strains (Supplementary Figs. S4–S6). As CES enzymes primarily catalyze the conversion of capecitabine to 5’-DFCR, we also plotted the tissue 5’-DFCR-to-capecitabine ratios. These findings clearly reflected the tissue-specific activity of hCES2 in the liver and intestine (Fig. 4c, f, i, l, o). FBAL showed somewhat higher tissue exposure in the transgenic mice, especially in the *Ces2*^*–/–*^A mice, but this reflected the higher plasma levels, as determined from the tissue-to-plasma ratios (Supplementary Fig. S7).

### Mouse Ces2 markedly affects vinorelbine metabolism but human CES2 only has a minimal effect: validation in male mice

Vinorelbine (Navelbine), a semisynthetic vinca alkaloid agent, is used for the treatment of advanced non-small cell lung cancer (NSCLC) and breast cancer [37–39]. Two main vinorelbine metabolites have been identified in humans: inactive CYP3A4-generated vinorelbine N-oxide [40, 41] and active deacetylvinorelbine, which is assumed to be formed by carboxylesterase enzymes [42] (Supplementary Fig. S8). A previous study suggested that in mice, hepatic mCes2a, but not mCes1, was likely involved in this process [12]. Vinorelbine might therefore represent a relatively Ces2-specific drug substrate. Thus, to study the metabolic impact of the mCes2 family and hCES2 on vinorelbine-to-deacetylvinorelbine conversion in vivo, we administered vinorelbine (10 mg/kg) either orally or intravenously (i.v.) to male wild-type, *Ces2*^*–/–*^, *Ces2*^*–/–*^A and *Ces2*^*–/–*^V mice. Then, we measured drug and metabolite levels in plasma and tissues. As there was no sex preference, these experiments were performed in male mice on the basis of mouse availability.

**Fig. 5:**
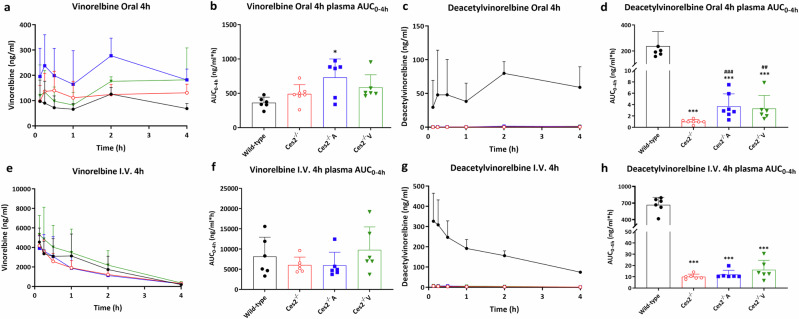
Vinorelbine plasma pharmacokinetic results in CES2-modified mouse models. Plasma concentration-time curves and AUC_0–4h_ of vinorelbine and its metabolite deacetylvinorelbine in male wild-type, *Ces2*^*–/–*^, *Ces2*^*–/–*^A and *Ces2*^*–/–*^V mice over 4 h after oral administration (**a**–**d**) or intravenous injection (**e**–**h**) of 10 mg/kg vinorelbine. Data are given as mean ± SD (*n* = 6–7). *, *P* < 0.05; ***, *P* < 0.001 compared to wild-type mice. ^##^, *P* < 0.01; ^###^, *P* < 0.001 compared to *Ces2*^*–/–*^ mice. Statistical analysis was applied after log-transformation of linear data.

For the oral experiments, no significant difference in the vinorelbine plasma AUC_0-4h_ was noted between the wild-type and *Ces2*^*–/–*^ mice; however, at most time points, the concentrations were greater in the *Ces2*^*–/–*^ mice. We did observe significantly greater plasma levels of vinorelbine in *Ces2*^*–/–*^A mice than in wild-type but not *Ces2*^*–/–*^ mice, whereas the *Ces2*^*–/–*^V values were also higher than those in wild-type mice but not significantly (Fig. 5a, b). Interestingly, the plasma AUC_0-4h_ of the metabolite deacetylvinorelbine was profoundly lower (238-fold) in *Ces2*^*–/–*^ mice than in wild-type mice (1.0 ± 0.37 vs. 238 ± 112 ng/mL*h, *P* < 0.001) (Fig. 5c, d and Table 2). Unexpectedly, the deacetylvinorelbine plasma AUC_0-4h_ was only modestly (albeit significantly) increased in both *Ces2*^*–/–*^A (3.7-fold, 3.7 ± 2.2 ng/mL*h, *P* < 0.001) and *Ces2*^*–/–*^V (3.3-fold, 3.3 ± 2.3 ng/mL*h, *P* < 0.001) mice compared with *Ces2*^*–/–*^ mice (Fig. 5c, d and Table 2). These low plasma deacetylvinorelbine levels accordingly yielded a profoundly lower plasma AUC_0-4h_ ratio of deacetylvinorelbine-to-vinorelbine in *Ces2*^*–/–*^, *Ces2*^*–/–*^A and *Ces2*^*–/–*^V mice than in wild-type mice (Table 2).

**Table 2:** Plasma pharmacokinetic parameters of vinorelbine and its active metabolite deacetylvinorelbine in male wild-type, *Ces2*^*-/-*^*, Ces2*^*-/-*^A and *Ces2*^*-/-*^V mice over 4 h after oral administration or i.v. injection of 10 mg/kg vinorelbine.

Vinorelbine parameter	Genotype/Groups							
	Oral administration				I.V. injection			
	Wild-type	*Ces2*^*–/–*^	*Ces2*^*–/–*^A	*Ces2*^*–/–*^V	Wild-type	*Ces2*^*–/–*^	*Ces2*^*–/–*^A	*Ces2*^*–/–*^V
AUC_0-4h_, ng/mL*h	362 ± 81	491 ± 135	730 ± 270*	585 ± 186	8143 ± 4784	6043 ± 1954	5949 ± 3247	9792 ± 5674
*C*_max_, ng/mL	149 ± 59	188 ± 63	333 ± 99**^#^	243 ± 110	N.A.	N.A.	N.A.	N.A.
*T*_max_, h	1.7 ± 0.7	1.9 ± 1.9	1.3 ± 0.9	2.4 ± 1.5	N.A.	N.A.	N.A.	N.A.

Deacetylvinorelbine parameter	Genotype/Groups							
	Oral administration				I.V. injection			
	Wild-type	*Ces2*^*–/–*^	*Ces2*^*–/–*^A	*Ces2*^*–/–*^V	Wild-type	*Ces2*^*–/–*^	*Ces2*^*–/–*^A	*Ces2*^*–/–*^V
AUC_0-4h_, ng/mL*h	238 ± 112	1.0 ± 0.4***	3.7 ± 2.2***^###^	3.3 ± 2.3***^###^	666 ± 138	10.1 ± 2.3***	12.0 ± 3.8***	16.2 ± 8.4***
AUC_0-4h_ ratio (× 10^−3^) (deacetylvinorelbine/vinorelbine)	640 ± 170	2.0 ± 0.4	6.3 ± 7.8***^#^	5.3 ± 1.9***^##^	110 ± 60	1.7 ± 0.2***	2.2 ± 0.5***	1.7 ± 0.2***
*C*_max_, ng/mL	91 ± 44	0.42 ± 0.15***	1.5 ± 1.1***^##^	1.4 ± 1.2***^##^	346 ± 104	5.9 ± 1.4***	7.6 ± 2.7***	8.2 ± 3.5***
*T*_max_, h	1.7 ± 0.7	3.7 ± 0.8*	2.6 ± 1.0	2.7 ± 1.0	0.46 ± 0.76	0.13 ± 0.00	0.25 ± 0.19	0.17 ± 0.07

Data are given as mean ± SD (*n* = 6). AUC_0-4h_, area under the plasma concentration-time curve; *C*_max_, maximum concentration in plasma; *T*_max_, time point (h) of maximum plasma concentration; *N.A.* Not Applicable; *, *P* < 0.05; **, *P* < 0.01; ***, *P* < 0.001 compared to wild-type mice. ^#^, *P* < 0.05; ^##^, *P* < 0.01; ^###^, *P* < 0.001 compared to *Ces2*^*–/–*^ mice. No statistical difference was found between *Ces2*^*–/–*^A and *Ces2*^*–/–*^V mice. Statistical analysis was applied after log-transformation of linear data and compared within either oral administration groups or i.v. injection groups.

With respect to tissues, similar to the plasma results, we observed that vinorelbine concentrations at 4 h were higher in all three gene-modified mouse strains than in wild-type mice. Nonetheless, for most tissues, there were no meaningful differences in tissue-to-plasma ratios (Supplementary Fig. S9, S10). However, for the liver, we observed significantly higher vinorelbine tissue-to-plasma ratios in *Ces2*^*–/–*^ and *Ces2*^*–/–*^V mice than in wild-type mice, whereas this increase was not observed in *Ces2*^*–/–*^A mice (Supplementary Fig. S9b). A pronounced decrease in deacetylvinorelbine generation was observed in *Ces2*^*–/–*^ mice compared with wild-type mice in most tissues, with limited but mostly significant increases again in both *Ces2*^*–/–*^A and *Ces2*^*–/–*^V mice, except for the intestinal compartments (Supplementary Fig. S11, S12). To better understand the transformation of vinorelbine to deacetylvinorelbine, we also calculated the ratios of the concentrations of deacetylvinorelbine to vinorelbine in all the tissues. In the liver, the deacetylvinorelbine-to-vinorelbine ratio in wild-type mice was 5.0 ± 1.0. This ratio was strongly reduced in *Ces2*^*–/–*^ mice (333-fold decrease), *Ces2*^*–/–*^A mice (263-fold decrease) and *Ces2*^*–/–*^V mice (238-fold decrease) (Supplementary Fig. S17a and Supplementary Table 4). In the small intestine tissue, the transformation was somewhat lower in wild-type mice with a ratio of 0.88 ± 0.22. However, Ces2 deficiency further markedly decreased this ratio to 0.034 ± 0.010, and transgenic hCES2 only slightly increased the ratios to 0.039 ± 0.008 and 0.049 ± 0.015 in *Ces2*^*–/–*^A and *Ces2*^*–/–*^V mice, respectively (Supplementary Fig. S17b and Supplementary Table 4). Similar results were obtained in other tissues and matrices, including the spleen, kidney, small intestine contents and colon (data not shown).

The results of the i.v. experiments were qualitatively similar to those of the oral administration experiments. Although the overall plasma levels of i.v. vinorelbine were much greater than those noted for oral vinorelbine, the plasma AUC_0-4h_ of deacetylvinorelbine in wild-type mice (666 ± 138 ng/mL*h) was still markedly reduced in *Ces2*^*–/–*^ mice (66-fold decrease), *Ces2*^*–/–*^A mice (55.5-fold decrease) and *Ces2*^*–/–*^V mice (41.1-fold decrease) (Fig. 5e–h and Table 2). With respect to tissues, mCes2 deficiency drastically reduced the deacetylvinorelbine-to-vinorelbine ratio in the liver from 2.5 ± 1.3 in wild-type mice to 0.021 ± 0.002 in *Ces2*^*–/–*^ mice, and hCES2 did not seem to greatly alter the ratio, with values of 0.025 ± 0.0070 in *Ces2*^*–/–*^A mice and 0.022 ± 0.007 in *Ces2*^*–/–*^V mice. In the small intestine, wild-type mice presented a somewhat lower conversion ratio (0.63 ± 0.05), and the ratio decreased to 0.015 ± 0.001 in *Ces2*^*–/–*^ mice, 0.014 ± 0.001 in *Ces2*^*–/–*^A mice and 0.017 ± 0.002 in *Ces2*^*–/–*^V mice (Supplementary Fig. 17c, d and Supplementary Table 4). The results obtained for the kidney, lung, spleen, small intestine contents, and colon were mostly qualitatively consistent with what we observed for the oral vinorelbine experiment (Supplementary Fig. 13–16, conversion data not shown).

### Ces2 deficiency does not noticeably affect irinotecan disposition

In humans, CES2 is thought to be the primary enzyme hydrolyzing the anticancer prodrug irinotecan, although CES1 certainly also contributes markedly [22, 43]. Unexpectedly, we found no significant effect of mCes2 cluster knockout on the plasma levels of irinotecan (either i.v. or orally administered) or its primary metabolite SN-38 (data not shown). We previously reported that mCes1 enzymes, especially mCes1c, which is highly abundant in plasma, have a major effect on irinotecan and SN-38 pharmacokinetics in mice [31]. Because all mCes1 genes remain fully active in *Ces2*^*–/–*^ mice, mCes1-mediated irinotecan hydrolysis may have dominated and obscured any separate contribution of mCes2. Consistent with this high impact of mCes1, irinotecan pilot experiments in hCES2-transgenic strains also did not yield useful additional information (data not shown). These negative results are not further discussed in this paper.

### Mouse Ces2 and human CES2 affect body weight and white adipose tissue adipositis

The experiments described above established a clear pharmacological role of Ces2/CES2 in our mouse models, as well as the functionality of various gene modifications, especially the human CES2 transgenes. We next performed a series of basic physiological studies in the CES2 gene-modified mouse strains (*Ces2*^*–/–*^, *Ces2*^*–/–*^A and *Ces2*^*–/–*^V) and wild-type mice under relatively moderate conditions using a standard medium‒fat diet (24% caloric intake from fat). Body weight was monitored between 4 and 20 weeks of age for both female and male mice of all the strains (*n* = 19–20). At 20 weeks, all the mice were fasted overnight and sacrificed, and liver and gonadal white adipose tissue (gWAT) were collected and weighed to explore potential changes in body weight.

**Fig. 6:**
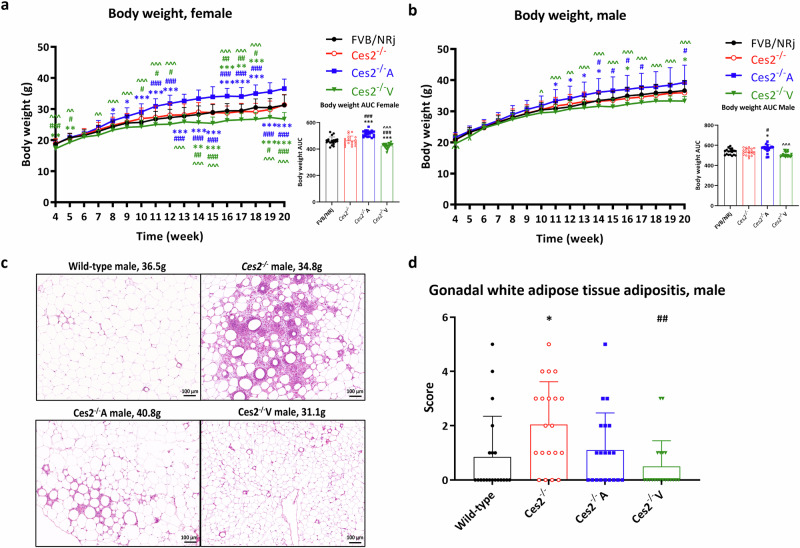
Basic physiological analysis of the CES2 mouse models. Body weight development from 4 to 20 weeks of age for females (**a**) and males (**b**); representative images of gonadal white adipose tissue of male mice from each mouse strain (**c**) and semi-quantified gonadal white adipose tissue adipositis levels (**d**) of 20-week-old wild-type, *Ces2*^*–/–*^, *Ces2*^*–/–*^A and *Ces2*^*–/–*^V mice after body weight measurement from 4 weeks to 20 weeks of age. Data are given as mean ± SD (*n* = 19–20). *, *P* < 0.05; **, *P* < 0.01; ***, *P* < 0.001 compared to wild-type mice. ^#^, *P* < 0.05; ^##^, *P* < 0.01; ^###^, *P* < 0.001 compared to *Ces2*^*–/–*^ mice. ^, *P* < 0.05; ^^, *P* < 0.01; ^^^, *P* < 0.001 for comparison between *Ces2*^*–/–*^A and *Ces2*^*–/–*^V mice. Statistical analysis was applied after log-transformation of linear data. For the scoring criteria in panel (**d**), please refer to the supplementary methods section 2.5.1; the Kruskal-Wallis rank test was used.

During the whole monitoring period, the body weights of female or male *Ces2*^*–/–*^ mice did not differ from those of wild-type mice (Fig. 6a, b). The absolute weights of the liver and gWAT were also similar between the wild-type and *Ces2*^*–/–*^ mice, reflecting the body weight results. However, *Ces2*^*–/–*^A mice (especially females) presented an average greater body weight than did wild-type mice beginning at 8 weeks of age and greater body weight than did *Ces2*^*–/–*^ mice beginning at 11 weeks of age, and these differences gradually increased over time. Female *Ces2*^*–/–*^A mice also clearly presented increased liver and gWAT weights (Supplementary Fig. S18). In contrast, *Ces2*^*–/–*^V mice (especially females) had an average lower body weight than did wild-type and *Ces2*^*–/–*^ mice, even at 4 weeks of age. These differences became more pronounced after 10 weeks of age. The profiles of the male mice were similar, albeit somewhat less pronounced (Fig. 6a, b and Supplementary Fig. S18). Female and male *Ces2*^*–/–*^V mice had significantly lower gWAT weights than the *Ces2*^*–/–*^ mice did (Supplementary Fig. S18c). After correction for body weight, differences in the gWAT-to-body weight ratio were more pronounced than those in the liver-to-body weight ratio, with *Ces2*^*–/–*^A female but not male mice showing higher gWAT-to-body weight ratios. In contrast, these ratios were significantly lower in both female and male *Ces2*^*–/–*^V mice than in *Ces2*^*–/–*^ mice (Supplementary Fig. S18e). This finding potentially indicates that the body weight differences were due mainly to changes in adipose tissue (Supplementary Fig. S18). Female and male aged mice (*n* = 3–6) at approximately 60 weeks of age were also sacrificed, and body and organ weights were measured. The results for all the strains were similar to those for the 20-week-old mice, especially the females (data not shown).

To better understand the reasons for the body weight differences, young adult mice between 12 and 13 weeks of age (*n* = 9–16) were sacrificed and dissected. Body weights and weights of different organs, including the liver, kidney, spleen, lung, and heart, and weights of different adipose tissues, including inguinal, perigonadal, mesenteric and retroperitoneal white adipose tissue (iWAT, gWAT, mWAT, and rWAT, respectively) and interscapular brown adipose tissue (iBAT), were measured (Supplementary Fig. S19). The body weight results were consistent with our earlier results in the 20-week experiment, with no difference noted between wild-type and *Ces2*^*–/–*^ mice with respect to both absolute organ and white adipose tissue (WAT) weights and relevant ratios corrected for body weight. *Ces2*^*–/–*^A mice had greater body weights than wild-type and *Ces2*^*–/–*^ mice did, and this difference was also consistently (albeit not always significant) reflected in the weights of the liver, other organs, WAT and BAT (Supplementary Fig. S19a, c). After correction for body weight, most of these differences disappeared (Supplementary Fig. S19b, d). Compared with wild-type and *Ces2*^*–/–*^ *mice, Ces2*^*–/–*^A male mice presented slightly but significantly greater liver-to-body weight ratios and spleen-to-body weight ratios. In contrast, the weights of *Ces2*^*–/–*^V mice were similar to or slightly lower than those of *Ces2*^*–/–*^ mice; however, the overall difference was less pronounced than that noted at 20 weeks of age. The differences in absolute organ and WAT weights were mostly related to body weight differences. Compared with *Ces2*^*–/–*^ female mice, *Ces2*^*–/–*^V mice presented a significantly lower mWAT-to-body weight ratio. No other differences were observed in the organ or WAT-to-body weight ratios.

A whole-body histology/pathology examination was performed on 20-week-old mice and aging mice. Although female mice presented clear and pronounced differences in body weight between the strains, there were no marked histological abnormalities. However, in males, we detected pronounced gonadal white adipose tissue inflammation (adipositis) in *Ces2*^*–/–*^ mice, and hCES2 somewhat rescued this phenotype in *Ces2*^*–/–*^A mice and very effectively rescued it in *Ces2*^*–/–*^V mice (for representative images, see Fig. 6c). Semi-quantification of adipositis (Fig. 6d) revealed that mCes2 deficiency resulted in significantly greater adipositis than seen in wild-type mice, and hCES2 expression in the liver mostly reversed this effect. However, hCES2 expression in the intestine profoundly reversed adipositis, resulting in an even lower average score than observed in wild-type male mice (Fig. 6d). In contrast, no significant adipositis was observed in the gWAT of females in any of the strains (data not shown), despite the significant changes in gWAT mass. This finding is consistent with our previous observations that female mice from the FVB/NRj background are much less prone to developing gWAT adipositis than male mice are [31]. A general histology/pathology examination was also performed in ~60-week-old aging mice. However, except for some basic geriatric disease phenotypes (such as focal necrotizing hepatitis), no particular pathology was observed, whereas all strains at this age presented similar levels of mild to severe adipositis (data not shown).

### Mouse Ces2 and human CES2 alter plasma lipid concentrations

20-week-old mice of all four strains were subjected to plasma chemistry and hematological examinations. The total plasma cholesterol concentrations were significantly greater in *Ces2*^*–/–*^ mice compared with wild-type mice, especially in females (Supplementary Fig. S20l). A similar profile for plasma HDL and, in particular, LDL cholesterol was observed (Supplementary Fig. 20j–k). The plasma triglyceride levels were not significantly different between *Ces2*^*–/–*^ and wild-type mice, nor were any of the other analyzed parameters (Supplementary Fig. S20). Hepatic transgenic hCES2 did not significantly lower the increased total, HDL, or LDL plasma cholesterol levels. However, intestinal transgenic hCES2 did limit cholesterol exposure, with clear decreases in total, high-density lipoprotein (HDL), and low-density lipoprotein (LDL) cholesterol levels in female but not male *Ces2*^*–/–*^ mice. Reduced values of several other plasma parameters, such as triglyceride levels and levels of the liver damage markers alkaline phosphatase (ALP) and alanine transaminase (ALAT) in males, were also detected in both strains and especially the enterocyte transgenic mouse strains (Supplementary Fig. S20). Although the differences were all modest, the shifts always occurred in a relatively “healthy” direction.

With respect to hematology, significantly lower mean corpuscular volume (MCV) and mean corpuscular hemoglobin (MCH) were observed in *Ces2*^*–/–*^ mice, and transgenic hCES2 increased these values in both *Ces2*^*–/–*^A and *Ces2*^*–/–*^V mice. In contrast, mCes2 deficiency led to a greater red cell distribution width (RDW), and the presence of hCES2 somewhat reversed this situation (Supplementary Fig. S21). Considering that the changes are limited and that all of these parameters are within a normal physiological range, these differences are probably not pathologically meaningful and will not be further discussed. The basic plasma clinical chemistry and hematologic analysis of aging mice ( ~ 60 weeks old) did not reveal any meaningful differences among the mouse strains (data not shown).

### hCES2 protects mice from liver lipid accumulation without influencing triglyceride secretion from the liver to the blood

**Fig. 7:**
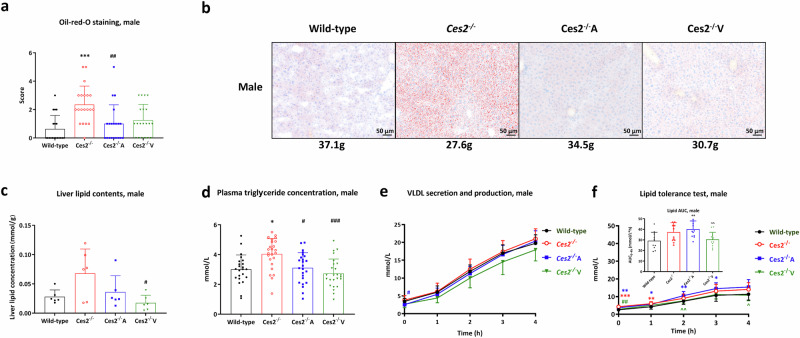
Lipid disposition, metabolism and homeostasis in the CES2 mouse models. Semi-quantified Oil-red-O staining lipid droplet levels (**a**) and representative Oil-red-O staining images for liver lipid accumulation for each mouse strain (**b**) in male wild-type, *Ces2*^*–/–*^, *Ces2*^*–/–*^A and *Ces2*^*–/–*^V 20-week old mice (*n* = 19–21; body weight of each individual mouse presented is indicated); (**c**) liver lipid contents in male wild-type, *Ces2*^*–/–*^, *Ces2*^*–/–*^A and *Ces2*^*–/–*^V 20-week old mice (*n* = 8); (**d**) plasma triglyceride basal concentration (overnight fast) before very low-density lipoprotein (VLDL) production and secretion and oral lipid tolerance test in male wild-type, *Ces2*^*–/–*^, *Ces2*^*–/–*^A and *Ces2*^*–/–*^V 12-week old mice (*n* = 20–24); (**e**) VLDL production and secretion and (**f**) oral lipid tolerance test in male wild-type, *Ces2*^*-/-*^, *Ces2*^*-/-*^A and *Ces2*^*-/-*^V 12-week old mice (*n* = 10–12). Data are given as mean ± SD. *, *P* < 0.05; **, *P* < 0.01; ***, *P* < 0.001 compared to wild-type mice. ^#^, *P* < 0.05; ^##^, *P* < 0.01; ^###^, *P* < 0.001 compared to *Ces2*^*-/-*^ mice. ^^, *P* < 0.01; for comparison between *Ces2*^*–/–*^A and *Ces2*^*–/–*^V mice. Statistical analysis was applied after log-transformation of linear data. For the scoring criteria in panel (**a**), please refer to the supplementary methods section 2.5.2; the Kruskal-Wallis rank test was used.

Oil Red O staining of the liver was performed, and the results were analyzed semi-quantitatively to assess the neutral lipid content. As frequently observed in mice, the hepatic lipid content in female wild-type mice was greater [44] and more resistant to diet-induced lipid accumulation than that in male mice and was not significantly altered in response to Ces2 deficiency. However, this lipid accumulation was somewhat increased by hepatocyte hCES2 expression but reduced by enterocyte hCES2 expression, and enterocyte hCES2 expression also yielded the lowest directly measured hepatic lipid content (Supplementary Fig. S22a–c). In contrast, in male mice, the absence of mCes2 profoundly increased hepatic lipid levels, and hCES2 significantly reduced these to levels similar to those in wild-type mice, especially in *Ces2*^*–/–*^A mice (Fig. 7a, b). The hepatic lipid concentration was increased due to the absence of mCes2 (albeit not statistically significant because of high variation), whereas both hepatic and especially intestinal hCES2 expression reduced the liver lipid concentration back to wild-type levels (Fig. 7c).

We further observed differences in the plasma triglyceride concentration (increased in *Ces2*^*–/–*^ and then decreased, especially in *Ces2*^*–/–*^V mice), primarily in males (Fig. 7d), but to a lesser extent also in females (Supplementary Fig. 22d). We therefore studied hepatic triglyceride secretion in these mouse strains after lipases were inhibited via the injection of poloxamer-407. However, we did not detect significant differences among the mouse strains in either females or males at any time point (Fig. 7e and Supplementary Fig. 22e), also after we normalized the results by subtracting the basal plasma triglyceride levels (Supplementary Fig. S23a, b). We next explored whether lipid uptake and/or chylomicron clearance were altered in the various CES2 mouse strains. For this purpose, an oral lipid tolerance test (oLTT) using olive oil delivered by gavage was performed in both female and male mice (*n* = 9–11). Compared with wild-type mice, *Ces2*^*–/–*^ mice had slightly higher plasma triglyceride concentrations during the experiments (0–4 h). Surprisingly, hepatocyte hCES2 expression even further increased these values. In contrast, enterocyte hCES2 expression reduced plasma triglyceride levels back to wild-type levels (Fig. 7f and Supplementary Fig. 22f). These phenotypes were further supported by the plasma triglyceride AUC_0-4h_ and were more obvious in male mice. However, normalization of the results by subtracting the baseline triglyceride concentrations revealed that *Ces2*^*–/–*^ mice had absolute triglyceride absorption levels similar to those of wild-type mice, and only hepatic hCES2 expression but not intestinal hCES2 expression slightly increased this absorption (Supplementary Fig. 23c, d).

### hCES2 improves glucose tolerance and insulin sensitivity

**Fig. 8:**
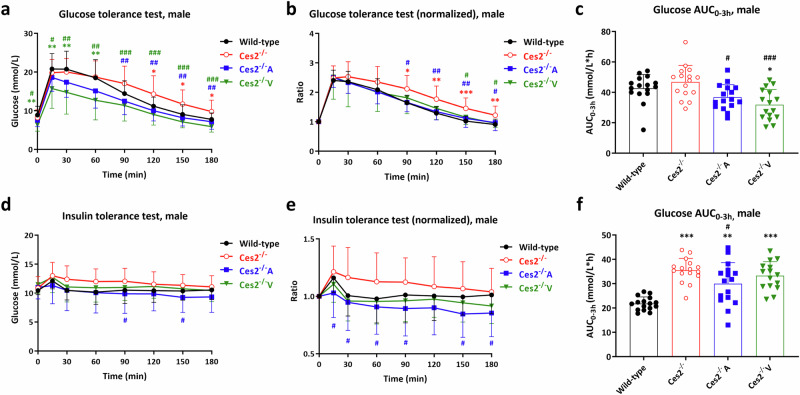
Glucose metabolism and homeostasis in the CES2 mouse models. The plasma glucose concentration time curve (**a**), plasma glucose concentration to basal glucose concentration (before glucose administration) ratio time curve (**b**) and glucose AUC_0-3h_ (**c**) in male wild-type, *Ces2*^*–/–*^, *Ces2*^*–/–*^A and *Ces2*^*–/–*^V 12-week old mice over a 3 h glucose tolerance test after oral administration of 1 mg/g glucose; The plasma glucose concentration time curve (**d**), plasma glucose concentration to basal glucose concentration (before insulin injection) ratio time curve (**e**) and glucose AUC_0-3h_ (**f**) in male wild-type, *Ces2*^*–/–*^, *Ces2*^*–/–*^A and *Ces2*^*–/–*^V 16-week old mice over a 3-h insulin tolerance test after i.p. injection of 0.5 U/kg insulin. Data are given as mean ± SD (*n* = 15–16). *, *P* < 0.05; **, *P* < 0.01; ***, *P* < 0.001 compared to wild-type mice. ^#^, *P* < 0.05; ^##^, *P* < 0.01; ^###^, *P* < 0.001 compared to *Ces2*^*–/–*^ mice. Statistical analysis was applied after log-transformation of linear data.

As CES2 expression induces changes in lipid metabolism, we also investigated the potential influence of CES2 on the interconnected glucose homeostasis. We therefore performed both oral glucose tolerance tests and insulin tolerance tests. *Ces2*^*–/–*^ female mice presented marginally higher blood glucose levels in the glucose tolerance test than wild-type mice did; however, the blood glucose levels were reduced by hCES2 expression, yielding even lower values than those in wild-type controls before 1 h, especially in *Ces2*^*–/–*^V female mice (Supplementary Fig. S24a–c). The differences were more pronounced in male mice, where both hCES2 transgenic mouse strains, especially *Ces2*^*–/–*^V, exhibited reduced plasma glucose levels compared with those in *Ces2*^*–/–*^ mice throughout the experimental period. These observations were further confirmed by the AUC data (Fig. 8a–c).

In the insulin tolerance test, females exhibited more sensitive responses to insulin challenge than male mice. In both sexes, *Ces2*^*–/–*^ mice clearly presented higher glucose concentrations than did wild-type mice. Hepatocyte hCES2 expression significantly decreased glucose levels after insulin injection in both sexes. In contrast, enterocyte hCES2 expression had an even greater effect on glucose levels, but this effect was primarily observed in female mice (Fig. 8d and Supplementary Fig. S24d). The ratio of the glucose concentration at each time point to the basal glucose level (T = 0) and the AUC results also confirmed these differences (Fig. 8e, f and Supplementary Fig. S24e, f).

## Discussion

In recent years, the roles of CESs, especially CES1 and CES2, in the metabolism of both endogenous and xenobiotic compounds have promoted great interest in the discovery of CES modulators to regulate lipid metabolism or to optimize the activity of ester (pro)drugs [1, 4, 19, 45–49]. Lehner and coworkers studied the physiological functions of mCes1 using single Ces1d or Ces1g knockout mouse strains [50–54]. Recently, we generated and characterized Ces1 cluster knockout (*Ces1*^*−/−*^) mice and a *Ces1*^*−/−*^ mouse strain expressing transgenic hCES1 in the liver. We found that both mCes1 and hCES1 are substantially involved in irinotecan and capecitabine metabolism, as well as lipid and glucose homeostasis, in vivo [31]. Compared with CES1, research on the physiological functions of CES2 remains limited. Recently, several papers focused on mouse *Ces2a* and *Ces2c* as well as hCES2 functions in metabolic syndrome by knocking down or knocking out the *Ces2a* and *Ces2c* genes and/or overexpressing hCES2 using adenoviral vectors [24–27]. To the best of our knowledge, no Ces2 family knockout mouse models have been described to date, and stable hepatic or intestinal hCES2 transgenic models in a fully mCes2-deficient background have not been established. In this study, we generated and characterized an entire Ces2 cluster knockout mouse model that exhibited no meaningful expression changes in other *Ces* genes. We further generated hepatic and intestinal hCES2 transgenic models on a mCes2-deficient background. Although slight renal expression of human CES2 was observed in these models, its contribution to overall drug metabolism is probably modest, considering that the liver is the main detoxification organ and that the intestine is a primary location for first-pass metabolism. All the above-mentioned mouse models were fully viable and fertile.

Using these mouse models, we found that mCes2 can markedly limit the oral availability of capecitabine, whereas enterocyte hCES2 expression, but not hepatocyte hCES2 expression, can also substantially decrease capecitabine plasma exposure (Fig. 3a). These findings suggest that hCES2 plays an important role in the first-pass intestinal metabolism of capecitabine. However, neither mCes2 nor hCES2 activity markedly influenced the levels of capecitabine metabolites, including 5-FU. The plasma concentrations of 5’-DFCR, the first hydrolysis product of capecitabine, were considerably higher than those of capecitabine in all strains (Fig. 3c), and the 5’-DFCR concentrations did not significantly differ between the strains. It therefore seems most likely that other esterases, including mCes1 enzymes [31], still catalyze very substantial formation of 5’-DFCR, converting a significant portion of the available capecitabine in plasma (potentially 70%–80% or more based on the plasma 5’-DFCR/capecitabine ratios we observed). However, Ces2 efficiently converts most of the remaining capecitabine, so the deletion of Ces2 still results in a marked increase in the plasma capecitabine concentration. Because the impact of Ces2 on the total amount of 5’-DFCR formed is relatively limited, the levels of 5’-DFCR and other metabolites are not significantly affected by the absence of Ces2.

The different tissue capecitabine exposure profiles between the liver and intestinal hCES2 transgenic mouse strains demonstrated specific local effects of hCES2 on capecitabine hydrolysis in the liver (Supplementary Fig. S3a, b) and small intestine (Supplementary Fig. S3g, h). The reduced kidney-to-plasma ratio in *Ces2*^*–/–*^A transgenic mice (Supplementary Fig. S3c, d) likely reflects the renal expression of transgenic hCES2, as shown in Fig. 2d. These local activities are especially apparent based on the 5’-DFCR/capecitabine ratios (Fig. 4c, f, i, l, o). The significantly increased intestine 5’-DFCR/capecitabine ratios in *Ces2*^*–/–*^V mice (Fig. 4l) suggest that enterocyte hCES2 expression, but not hepatocyte hCES2 expression, directly affects the first-pass metabolism of oral capecitabine and, subsequently, the exposure noted in other tissues. This finding may also apply to other esterase-sensitive drugs (or prodrugs), potentially further inducing local toxicity in tissues (such as chemotherapy-induced diarrhea) or efficacy in solid tumors. We obtained analogous results for *Ces2*^*–/–*^A mice, where the liver 5’-DFCR/capecitabine ratios were especially high (Fig. 4c). Collectively, our capecitabine results clearly demonstrate the role of CES2 in the initial hydrolysis of capecitabine and validate the in vivo enzymatic functionality of both the liver and intestinal hCES2 transgenes in converting capecitabine to 5’-DFCR. However, they also suggest that this may have only a limited effect on the formation and exposure of the active compound 5-FU, presumably because of the activity of alternative esterases in the body. Notably, in several clinical pharmacokinetic studies with single oral doses of capecitabine (1250 to 1255 mg/m^2^), the mean *C*_*max*_ of 5-FU ranged from 218 ng/mL to 310 ng/mL [55]. In our mouse models, the mean *C*_*max*_ of 5-FU was of the same order of magnitude, ranging from 105 ng/mL to 159 ng/mL, illustrating the relevance of the dose of 500 mg/kg we used (Table 1).

We further explored the contribution of CES2 enzymes to the conversion of vinorelbine to deacetylvinorelbine and how this affects both plasma exposure and tissue distribution. The deficiency of mCes2 led to a marked decrease in the deacetylvinorelbine plasma AUC_0-4h_ (238-fold decrease upon i.v. injection and 66-fold decrease upon oral administration), as well as the plasma AUC_0-4h_ ratio of deacetylvinorelbine to vinorelbine, compared with those of wild-type mice (Fig. 5c, d and Table 2). However, human CES2 only marginally rescued deacetylvinorelbine formation after oral administration. Notably, although abundant levels of mCes1 enzymes are present in *Ces2*^*–/–*^ mice, including a high amount of mCes1c in blood, deacetylvinorelbine generation is extremely low when mCes2 is absent. These findings indicate that the conversion of vinorelbine to deacetylvinorelbine is primarily mediated by one or more mCes2 enzymes but not mCes1 or other esterases. The finding that hCES2 had only a very limited effect on vinorelbine metabolism in mice was unexpected since the preceding capecitabine experiments demonstrated good functionality of the transgenic hCES2 enzymes. It is possible that human CES2 hydrolyzes vinorelbine very poorly. However, our previous study suggested that mCes2 proteins (likely mCes2a) may be secreted in part into the blood and contribute to vinorelbine hydrolysis, despite the presence of an HXEL ER retention signal in mCes2a [12]. This might disproportionately influence vinorelbine metabolism directly in the circulation, in contrast to transgenic hCES2, which is restricted to the liver or intestine. Notably, when 80 mg/m^2^ vinorelbine was orally administered to 24 patients, the plasma *C*_*max*_ values of vinorelbine and deacetylvinorelbine were 133.4 ± 42.3 and 9.1 ± 3.3 ng/mL, respectively [42]. These results indicate that the effects of absolute exposure to vinorelbine and its metabolite deacetylvinorelbine are similar in magnitude to those observed in our transgenic mice (Table 2). Overall, the vinorelbine results in our mouse models indicate that vinorelbine is a highly specific substrate of mCes2 but much less of hCES2.

Dietary lipids, including triglycerides and cholesterol, are taken up by enterocytes from the small intestine and packaged into chylomicrons, which are subsequently secreted into the lymph stream and then metabolized by lipoprotein lipase (LPL), releasing free fatty acids into the circulation. The remaining compounds are used in the formation of LDL and HDL and are then distributed in various tissues, including the liver. In the liver, free fatty acids are assembled, accumulated, re-esterified to glycerides and stored in lipid droplets (LDs). When secreted back into the circulation, this occurs mainly in the form of VLDL particles [56–59]. Carboxylesterases can hydrolyze many lipids, including triglycerides and cholesterol esters, thus affecting lipid homeostasis, atherosclerosis and inflammation, obesity, and type 2 diabetes (i.e., metabolic syndrome) [24–27, 50–54, 60, 61]. However, to date, physiological studies on CES2 have been more limited than those on CES1. We therefore applied a number of physiological studies in the CES2-modified mouse strains we generated.

The absence of all mouse Ces2 genes did not affect body weight development, which is consistent with some other recent studies on single *Ces2* gene knockout strains [24, 27]. However, mCes2 deficiency may increase lipid accumulation in the liver, especially in males (Fig. 7a–c). The disruption of lipid homeostasis in the liver may further induce remote white adipose tissue adipositis (Fig. 6c, d) [62]. mCes2 deficiency further increased plasma cholesterol levels in both genders but did not affect plasma triglyceride levels in 20-week-old mice (Supplementary Fig. S20). However, although Ces2 had no significant direct influence on hepatic lipid secretion or perhaps oral lipid absorption, relatively high basal plasma triglyceride concentrations in the VLDL and oLTT experiments were observed in 12-week-old *Ces2*^*–/–*^ male mice (Fig. 7d). Thus, depending on age, Ces2 can potentially and at least partly influence triglyceride levels in the circulation in males.

Compromised lipid homeostasis may induce glucose dysregulation and insulin resistance [63]. Indeed, *Ces2*^*–/–*^ mice presented slightly greater glucose levels than did wild-type mice in both the GTT and ITT (Fig. 8 and Supplementary Fig. S24). Although all our analyses were performed under medium-fat diet conditions, our results appear to be partly consistent with those of two recent studies, where either knockdown of hepatic *Ces2c* or global *Ces2a* knockout resulted in increased hepatic triglyceride levels without affecting VLDL secretion [24, 26]. Notably, hepatic diacylglycerol (DAG) and lysophosphatidylcholine (lysoPC) levels are markedly increased in high-fat diet-fed *Ces2a*-deficient mice, which also exhibit glucose intolerance, which is believed to occur via PKC activation due to DAG overaccumulation [26].

Surprisingly, *Ces2*^*–/–*^A mice presented greater body weights than both wild-type and *Ces2*^*–/–*^ male and especially female mice did, and this difference appeared to be driven mainly by greater liver and adipose tissue weights at both 12 and 20 weeks of age (Fig. 6 and Supplementary Fig. S18 and S19). Despite these seemingly adverse changes, hepatic hCES2 alleviated WAT adipositis and liver lipid overaccumulation in males compared with those in *Ces2*^*–/–*^ mice (Figs. 6c and d and 7). Compared with *Ces2*^*–/–*^ mice, hepatic hCES2 expression did not significantly alter VLDL production or plasma triglyceride exposure after oral lipid administration. However, compared with those of wild-type males and females, plasma triglyceride levels were significantly increased after oral lipid administration (Fig. 7e, f and Supplementary Fig. S22e, f). This latter effect may be partially responsible for the overall greater body weight and WAT weight in the *Ces2*^*–/–*^A mice. However, compared with *Ces2*^*–/–*^ mice, hepatic hCES2 expression did reduce the basal plasma triglyceride concentration in males but not in females (Fig. 7d). In some ways, *Ces2*^*–/–*^A mice seem to display a form of “healthy” obesity, characterized by increased body weight and WAT and liver compartment size. However, these mice also show reduced WAT inflammation and hepatic steatosis and improved responses in the GTT and ITT assays compared with *Ces2*^*–/–*^ mice (Fig. 8 and Supplementary Fig. S24).

Our data are again partly consistent with some of the abovementioned studies in C57BL/6 (generally only male) mice [24–26]. Li et al. demonstrated that hepatic hCES2 overexpression in HFD-fed mice had no effect on plasma triglyceride or cholesterol levels but reduced hepatic triglyceride levels and improved glucose tolerance. It appears that the gain of hepatic CES2 function attenuates hepatic triglyceride accumulation, likely through both enhancing fatty acid oxidation and inhibiting lipogenesis by reducing SREBP-1c transcription and processing [24]. However, why hepatic hCES2 expression increased the size of the liver and WAT and BAT compartments in *Ces2*^*–/–*^ mice with an FVB/N background remains unclear. Similarly, Ruby et al. revealed that hepatic hCES2 expression increased TAG/DAG hydrolysis activity, reversed high-fat diet-induced hepatic steatosis, and reduced hepatic TAG levels, which is partly consistent with our findings. However, it also decreases plasma cholesterol levels without affecting mouse body weight [25], which contrasts with our results. Ruby et al. further showed that despite the activation of ER stress, as well as that of the downstream effector proteins IKK and JNK, hepatic hCES2 overexpression may promote either independent or IRS2-related Akt activation, which further improves glucose tolerance [25], as was also observed in *Ces2*^*–/–*^A mice. Very recently, Chalhoub et al. reported that HFD-induced hepatic DAG accumulation and glucose intolerance in single *Ces2a* knockout mice can be reversed upon ectopic hCES2 expression without reducing liver or body weight [26]. Our own findings of alleviation of the hepatic lipid burden, improved glucose tolerance, and amelioration of WAT adipositis in *Ces2*^*–/–*^A mice are again partly consistent. Furthermore, even though the potential mechanisms remain unclear, it seems that hepatocyte hCES2 expression can enhance triglyceride clearance from the circulation in the long term. This likely occurs by increasing adipose tissue storage under healthy conditions, which increased body weight in our study. We note that there may be many explanations for the partial differences between our findings and those of previous studies, including the background mouse strain used (FVB/N vs. C57BL/6), the use of medium- or high-fat diet conditions, the knockout of single or all mouse *Ces2* genes, the continued presence of most or even all endogenous *Ces2* genes in hCES2 ectopic overexpression models, and the limitations associated with the use of only male mice in most of the cited studies.

Notably, some clear sex-dependent differences in the behavior of the Ces2 knockout and hCES2 liver transgenic mouse strains were observed. In male mice, Ces2 knockout significantly increased gWAT inflammation (Fig. 6c, d), increased liver Oil Red O staining and lipid concentrations, and increased plasma triglyceride levels, and all these phenotypes were partially or completely reversed by hepatic hCES2 expression (Fig. 7a–d). In contrast, in females, Ces2 knockout did not result in significantly increased gWAT adipositis (not shown), hepatic lipid accumulation or plasma triglyceride levels, and hepatic hCES2 expression did not further alter these parameters (Supplementary Fig. S22a–d). Although the mechanistic cause of these sex differences remains unknown, we previously reported similar sex-dependent differences in the development of gWAT adipositis in male but not female Ces1 knockout mice [31]. More generally, there are many examples of sex-dependent differences in lipid homeostasis, including in humans, which are still not completely understood.

Perhaps the most striking results were obtained with the intestinal transgenic expression of hCES2, as it induced physiological changes that are generally considered highly beneficial. Compared with *Ces2*^*–/–*^ mice and, occasionally, wild-type mice (Figs. 6 and 7 and Supplementary Fig. S18, S19, S20, S22), this expression not only decreased the body and WAT weights, which reflect lower body weights, but also considerably reduced the plasma cholesterol levels, especially in females, and profoundly restricted WAT inflammation and liver lipid content. Although enterocyte hCES2 expression did not significantly influence overall lipid absorption or liver VLDL production, it markedly decreased the basal plasma triglyceride concentrations compared with those in *Ces2*^*–/–*^ mice (Fig. 7 and Supplementary Fig. S22). Additionally, glucose tolerance and insulin resistance parameters were markedly improved in *Ces2*^*–/-–*^V mice, especially females (Fig. 8 and Supplementary Fig. S24). Similarly, a recent study indicated that after long-term high-fat diet feeding [27], transduced *Ces2c* (considered one of the orthologs of hCES2) abundantly expressed in the intestine in a *Ces2*-proficient mouse background also yielded a clear decrease in body weight. It further reduced plasma cholesterol levels and protected mice from liver steatosis compared with wild-type mice. Just as seen with our results, that study revealed that hepatic VLDL secretion, dietary fat absorption and chylomicron secretion rates were not altered in adenovirus-mediated enterocyte Ces2c-overexpressing [27] or hepatic *Ces2c*-knockdown mice [24]. However, postprandial triglyceride clearance was enhanced in enterocyte Ces2c-overexpressing mice. This could be related to somewhat increased chylomicron particle size and increased lipid shuttling into skeletal muscle (the mRNA expression of genes involved in fatty acid oxidation was increased in skeletal muscle but reduced in the livers of Ces2c enterocyte-overexpressing mice), thereby lowering lipid flux and triglyceride accumulation in the liver [27].

Our results are compatible with the possibility that enterocyte hCES2 expression may likewise increase triglyceride clearance from the circulation to muscle and thus decrease hepatic triglyceride accumulation and alleviate white adipose tissue expansion and inflammation. The improved lipid homeostasis may subsequently improve glucose tolerance and insulin resistance, as observed in both our study and the abovementioned study [27]. Regardless of the exact underlying mechanism(s), our data strongly suggest that stimulating high intestinal hCES2 expression or activity may be helpful in combatting or preventing the negative effects of developing metabolic syndrome.

Our findings further support that mouse Ces2 and human CES2 have bifunctional roles in both the detoxification of a range of drugs and other xenobiotics and in protection from possible toxic or adverse effects of (excess) lipids. This bifunctionality is similar to that previously described in various mouse models for mouse Ces1 and human CES1 proteins, as more extensively discussed in Gan et al. [31]. In future studies, investigating the possible functional overlap or redundancy between Ces2/CES2 and Ces1/CES1 with respect to both xenobiotic and lipid detoxification will be interesting.

In summary, we generated and characterized full Ces2 cluster deletion mice and two humanized CES2 transgenic mouse strains with targeted expression in either the liver or intestine. Our genetically engineered CES2 mouse models provide powerful preclinical tools for studying the pharmacological and physiological roles of the carboxylesterase 2 family. These mouse models are expected to facilitate the development of various (pro-)drug classes and improve drug administration regimens. In addition, a better understanding of the involvement of CES2 in lipid and energy metabolism processes and thus deeper physiological insights can be obtained. This could help us to further explore potential solutions for the metabolic syndrome.

## Supplementary information

Supplementary Methods

Supplementary Tables

Supplementary Fig. S1

Supplementary Fig. S2

Supplementary Fig. S3

Supplementary Fig. S4

Supplementary Fig. S5

Supplementary Fig. S6

Supplementary Fig. S7

Supplementary Fig. S8

Supplementary Fig. S9

Supplementary Fig. S10

Supplementary Fig. S11

Supplementary Fig. S12

Supplementary Fig. S13

Supplementary Fig. S14

Supplementary Fig. S15

Supplementary Fig. S16

Supplementary Fig. S17

Supplementary Fig. S18

Supplementary Fig. S19

Supplementary Fig. S20

Supplementary Fig. S21

Supplementary Fig. S22

Supplementary Fig. S23

Supplementary Fig. S24

Supplementary Figure Legend
