# Supplementary material for: Intestinal human carboxylesterase 2 (CES2) expression rescues drug metabolism and most metabolic syndrome phenotypes in global Ces2 cluster knockout mice
Source: Acta Pharmacol Sin. 2024 Nov 4;46(3):777–93. doi: 10.1038/s41401-024-01407-4 (PMC11845761; doi:10.1038/s41401-024-01407-4)
Supplement: Supplementary file 1 — Supplementary Methods [file 41401_2024_1407_MOESM1_ESM.docx]

**Supplementary Methods**

**2.2 Generation of *Ces2^-/-^*, *Ces2^-/-^*A and *Ces2^-/-^*V mice**

**2.2.1 *Ces2^-/-^* mice generation**

Mouse *Ces2* gene was searched at NCBI (https://www.ncbi.nlm.nih.gov/) and ENSEMBL database (http://www.ensembl.org/index.html), 5’-20 bp target sequence (5’-TCTTCCCTGCCCCCGTTCAAATCATCAGGGACTTAACATTA-3’) and 3’-20 bp target sequence (5’-AGCCTGAAATATTATTGCATTGGACGGGTAGGTGCTTCTGG-3’) were selected at ~3000 bp upstream of the *Ces2a* gene and ~2000 bp downstream of the *Ces2h* gene, respectively. In addition, two corresponding specifically designed (with *Eco*R V digestion site and RoxP sites introduced) homology-directed repair (HDR) oligos (5’-ATAGAGTGGAAACATCCTAAGTGGTCAATCTCACTGAGAGGAAGGTCTTCCCTGCCCCCGatatctttataactttaaataattggcattatttaaagttaggctTTCAAATCATCAGGGACTTAACATTATCCTTGTAATGGCCTTGTTTACACATCCTACCAG-3’ and 5’-TTGCTTTGTATCCTCGCAAATCCTTAAAGTAGTGAGCCTGAAATATTATTGCATTGGACGatatctttataactttaaataattggcattatttaaagttaggctGGTAGGTGCTTCTGGTTGTAGATGTTTCCCTAGATTTTATAGTGTCTGCTTCTAGAGAGA-3’) were micro-injected mixed with gRNAs (obtained by PCR, target sequence as template) and Cas9 mRNA into zygotes isolated from the FVB/NRj mouse strain. The whole *Ces2* cluster (from *Ces2a* to *Ces2h*) deletion and/or independent 5’-RoxP and 3’-RoxP insertion lines were expected to be generated. A candidate whole *Ces2* cluster deletion from two CRISPR/Cas9 cutting sites without RoxP insertion was obtained and backcrossed to wild-type (>99% FVB background) at least three generations to dilute any potential off-target events. The homozygous complete deletion of the *Ces2* cluster (*Ces2^-/-^*) was generated by crossbreeding among heterozygous knockout mice. *Ces2^-/-^* mice were verified by PCR for 7 individual *Ces2* genes (except for the pseudogene *Ces2d*) with wild-type mice as positive control (primers listed in Supplementary Table S1).

**2.2.2 Construction of the transgene liver-targeting expression plasmid**

In order to obtain liver-abundant expression of human CES2, a transgene construct was generated as follows (Fig. 2a). Briefly, pTargeT Mammalian Expression Vector containing human CES2 cDNA (NM_003869: 945–2889) (a gift from Prof. Nakajima, Kanazawa University, Kanazawa, Japan) (Fukami et al., 2010) was digested with *Sal* I and *Mlu* I to obtain a human CES2 cDNA linear fragment (1972 bp). pLIV-LE6 vector containing a hepatic control region (HCR) (a gift from Dr. J. Taylor, Gladstone Institute, University of California, San Francisco, USA) (Simonet et al., 1993) was digested with *Xho* I to obtain a linearized fragment. Then these two linear fragments were purified by Qiaquick PCR Purification Kit, blunted by Klenow enzyme, purified again and followed by Calf Intestinal Alkaline Phosphatase (CIP) treatment, and ligation using TaKaRa DNA Ligation Kit Ver. 2.1 (Catalog No: 6022) as last step. The ligated circular DNA product pLIV-LE6-hCES2-HCR was transformed into competent cell DH5a for mini-preparation. The resulting circular DNA product was digested with *Acl* I and *SaI* I to yield functional linearized ApoE-hCES2-HCR.

**2.2.3 Construction of the transgene intestine-targeting expression plasmid**

Another transgene was constructed for intestine-abundant expression of human CES2. Briefly, part of the hCES2 open reading frame was cloned behind the villin promoter by PCR. A forward 5’-CCTCTAGGCTCGTCCACCATGACTGCTCAGTCCCGCTCT-3’ primer with the last bases of the villin promoter and the first bases of hCES2 (including ATG-start sequence) and a reverse 5’-AATAGCGACGTCTCCGGATGGGACTGGCTGAGT-3’ primer aligning with the hCES2 sequence at its BspEI digestion site and followed by an introduced *Aat* II digestion site was used, using pTarget-hCES2 as a template. Subsequently, the obtained PCR product was elongated at its 5’ villin promoter side using pBluescript II KS-2kbvillin as a template (kindly provided by D. Louvard, Institut Curie, Paris, France) containing the last 2 kb 3’ of the villin promoter (Pinto, D., Robine, S., Jaisser, F., El Marjou, F.E., Louvard, D. (1999) Regulatory sequences of the mouse villin gene that efficiently drive transgenic expression in immature and differentiated epithelial cells of small and large intestines. J. Biol. Chem. 274: 6476-6482). This elongated product was used as the template for a subsequent PCR with forward 5’-GCCTTAAGCCGGCTGTGATAGC-3’ primer, aligning with the villin promoter sequence at its *Afl* II digestion site, and the earlier used reverse 5’-AATAGCGACGTCTCCGGATGGGACTGGCTGAGT-3’ primer, aligning with the hCES2 sequence at its BspEI digestion site and introduced a *Aat* II digestion site, and thereby a PCR product was generated spanning the last part of the villin promoter sequence connected with the ATG start sequence and subsequently the first part of hCES2, flanked by *Afl* II and *Aat* II digestion sites. The *Afl* II and *Aat* II digested fragment was then inserted into the digested *Afl* II and *Aat* II sites of pKS 9kbVill (kindly provided by D. Louvard) containing the full murine villin promoter that stops at the *Aat* II site, yielding pBluescript II KS-9kbVill-ATG-partial hCES2. The pTarget-hCES2 was digested by BspEI to obtain the fragment containing the second part of hCES2 together with the SV40 region. This linearized fragment was subsequently inserted into the BspEI-digested pBluescript II KS-9kbVillin-ATG-partial hCES2, yielding pBluescript II KS-9kbVillin-ATG-hCES2-SV40 plasmid. An *Eco*R V-*Aat* II excision of this clone was used to obtain functional linear insert Villin-hCES2-SV40 (Fig. 2b).

**2.2.4 *Ces2^-/-^*A and *Ces2^-/-^*V mice generation**

The two functional linearized fragments were subsequently injected by pronuclear injection into fertilized oocytes of *Ces2^-/-^* mice. Two-cell stage embryos were implanted into oviducts of pseudo-pregnant F1 fosters and carried to term. Transgenic founder lines among the offspring were detected by initial PCR screen with forward 5’-GCCTTAAGCCGGCTGTGATAGC-3’ and reverse 5’-CACCACTCCAAGATTCAGGG-3’ primers located in the villin promoter and hCES2 cDNA, respectively. DNA was extracted from toe clips of mice. Obtained transgenic mice with either ApoE-hCES2-HCR-driven liver-targeting or villin-hCES2-SV40-driven intestine-targeting expression of human CES2 were first back-crossed with FVB/NRj at least three generations and subsequently crossed to *Ces2^-/-^* mice in order to generate heterozygous hCES2 transgenics with homozygous *Ces2^-/-^* background. Further cross-breeding yielded homozygous transgenic mice (both *Ces2^-/-^* and human CES2 transgenes) referred to as *Ces2^-/-^*A and *Ces2^-/-^*V which were identified by real-time quantitative PCR with Qiagen human CES2 SYBR Green-based RT² qPCR Primer assays respectively. Transgenic hCES2 expression was monitored and was found to be stable around 9 generations (data not shown).

**2.5 Bodyweight monitoring, histology/pathology, plasma clinical chemistry and hematology analysis**

**2.5.1 Semi-quantitative assessment standard for (H&E)-staining of white adipose tissue**

For white adipose tissue (WAT) adipositis, the following standard of semi-quantification for each slide was used and assessed independently by a pathologist blinded to the identity of the strain on the slides:

Normal WAT tissue: -, score: 0;

Normal WAT tissue with limited mild local adipositis: -/+, score: 1;

Mild adipositis: +, score: 2;

Mild local adipositis with limited moderate local adipositis: +/++, score: 3;

Moderate adipositis: ++, score: 4;

Moderate local adipositis with severe local adipositis: ++/+++, score: 5;

Severe adipositis: +++, score: 6;

**2.5.2 Semi-quantitative assessment standard for Oil-red-O staining of liver lipid accumulation**

For Oil-red-O staining of liver lipid accumulation, the following standard of semi-quantification for each slide was used and assessed independently by a pathologist blinded to the identity of the strain on the slides:

Negative Oil-red-O staining (no lipid droplets): -, score: 0;

Negative Oil-red-O staining with limited mild (small lipid droplets) local lipid droplets: -/+, score: 1;

Mild Oil-red-O staining (small lipid droplets): +, score: 2;

Mild Oil-red-O staining with moderate (medium lipid droplets) local lipid droplets: +/++, score: 3;

Moderate Oil-red-O staining (medium lipid droplets): ++, score: 4;

Moderate Oil-red-O staining with severe (large lipid droplets) local lipid droplets: ++/+++, score: 5;

Severe Oil-red-O staining (large lipid droplets): +++, score: 6;
