# Supplementary material for: Intestinal human carboxylesterase 2 (CES2) expression rescues drug metabolism and most metabolic syndrome phenotypes in global Ces2 cluster knockout mice
Source: Acta Pharmacol Sin. 2024 Nov 4;46(3):777–93. doi: 10.1038/s41401-024-01407-4 (PMC11845761; doi:10.1038/s41401-024-01407-4)
Supplement: Supplementary file 2 — Supplementary Tables [file 41401_2024_1407_MOESM2_ESM.docx]

| Genes |  | Sequence | Length | Products (bp) |
| --- | --- | --- | --- | --- |
| *Ces2a* | FORWARD | TGAGCCAAGCAGAACATCAG | 20 | 335 |
|  | REVERSE | GCGAACCCTAAGCCTGGTT | 19 |  |
| *Ces2b* | FORWARD | TCAGCCTGGAGGCTAACTCT | 20 | 345 |
|  | REVERSE | GAGAGCTGAGGAGTCCCCTA | 20 |  |
| *Ces2c* | FORWARD | GCTTTACTGGTGTACGTGGC | 20 | 342 |
|  | REVERSE | ACAAAATGCCCTCTCCCCAA | 20 |  |
| *Ces2e* | FORWARD | TTCCTTGAGCTGGATGGAGTT | 21 | 471 |
|  | REVERSE | AGCAGTTGGGGGAAGGGTAA | 20 |  |
| *Ces2f* | FORWARD | CATAGCACACTGGGGGTCAG | 20 | 695 |
|  | REVERSE | TCCGCATGGCCTAGATGTTG | 20 |  |
| *Ces2g* | FORWARD | GGCATGAAACGTGAGTCTTCC | 21 | 285 |
|  | REVERSE | TGCCACCTCATGTAACTGCT | 20 |  |
| *Ces2h* | FORWARD | CTAACCACCGCGACCTGTG | 20 | 302 |
|  | REVERSE | TCTGCCACTCCTCAAAACCC | 21 |  |

Supplemental Table 1. The primers for PCR analysis of each *Ces2* gene present in the genome, except for the *Ces2d* pseudogene.

| **Tissue**  **Gene** | **Liver** | |  | **Small intestine** | |
| --- | --- | --- | --- | --- | --- |
|  | Wild-Type | *Ces2^-/-^* |  | Wild-Type | *Ces2^-/-^* |
| Ces1a | 12.8 ± 0.6 | 12.7 ± 0.5 |  | 14.4 ± 0.7 | 15.5 ± 1.2 |
| Ces1b | -3.0 ± 0.3 | -3.2 ± 0.4 |  | 3.7 ± 0.6 | 3.6 ± 0.3 |
| Ces1c | -1.9 ± 0.2 | -2.1 ± 0.5 |  | 7.4 ± 0.1 | 7.5 ± 0.3 |
| Ces1d | 1.9 ± 0.7 | 0.8 ± 0.9 |  | 6.4 ± 0.3 | 5.5 ± 1.0 |
| Ces1e | 2.1 ± 0.4 | 1.8 ± 0.2 |  | 6.7 ± 0.4 | 6.4 ± 0.3 |
| Ces1f | 2.0 ± 0.3 | 1.8 ± 0.3 |  | 3.9 ± 0.2 | 3.7 ± 0.3 |
| Ces1g | 3.2 ± 0.8 | 2.5 ± 0.7 |  | 6.1 ± 0.5 | 5.8 ± 0.5 |
| Ces1h | 14.7 ± 2.1 | 13.8 ± 1.2 |  | 10.4 ± 1.8 | 11.1 ± 1.2 |
| Ces2a | 2.1 ± 0.3 | 15.2 ± 1.2*** |  | 2.6 ± 0.4 | 14.4 ± 1.1*** |
| Ces2b | 4.1 ± 0.6 | 13.4 ± 0.6*** |  | 1.6 ± 0.3 | 12.7 ± 0.4*** |
| Ces2c | 2.1 ± 0.5 | 16.6 ± 0.5*** |  | 1.0 ± 0.2 | 16.9 ± 0.8*** |
| Ces2e | 3.2 ± 0.4 | 18.9 ± 1.3*** |  | 0.4 ± 0.2 | 20.6 ± 0.9*** |
| Ces2f | 17.2 ± 2.9 | 18.8 ± 1.2 |  | 13.9 ± 0.4 | 19.9 ± 0.9*** |
| Ces2g | 5.5 ± 0.3 | 16.3 ± 2.1*** |  | 6.7 ± 0.4 | 13.7 ± 0.9*** |
| Ces2h | 12.9 ± 0.8 | 18.2 ± 2.0** |  | 8.9 ± 0.8 | 16.5 ± 0.8*** |
| Ces3a | -0.04 ± 0.97 | -0.09 ± 0.61 |  | 10.69 ± 0.14 | 13.83 ± 0.52*** |
| Ces3b | 2.11 ± 0.64 | 1.97 ± 0.47 |  | 14.71 ± 0.27 | 17.94 ± 1.03*** |

Supplemental Table 2. Overview of ΔCt values of the RT-qPCR analysis to investigate expression of Ces1, Ces2 and Ces3 cluster genes in liver and small intestine of wild-type and *Ces2^-/-^* mice (*n* = 4). Quantification of the target cDNAs in all samples was normalized against the endogenous control GAPDH (ΔCt = C_target_ – C_GAPDH_). Accordingly, the lower the value, the higher the expression level. *, *P* < 0.05; **, *P* < 0.01; ***, *P* < 0.001 compared to wild-type mice.

Supplemental Table 3. Tissue pharmacokinetic parameters of capecitabine and its metabolites 5’-DFCR, 5’-DFUR, 5-FU and FBAL in female wild-type, *Ces2^-/-^, Ces2^-/-^*A and *Ces2^-/-^*V mice over 2 h after oral administration of 500 mg/kg capecitabine.

| **Tissue Parameter** | | **Genotype/Groups** | | | |
| --- | --- | --- | --- | --- | --- |
|  |  | **Oral administration** | | | |
|  |  | Wild-type | *Ces2^-/-^* | *Ces2^-/-^*A | *Ces2^-/-^*V |
| C_liver_  ng/g | Capecitabine | 1302 ± 522 | 18001 ± 8744*** | 4649 ± 1760**^##^ | 6566 ± 3220***^##^ |
|  | 5’-DFCR | 20310 ± 5741 | 33333 ± 11604 | 36650 ± 5867 | 23264 ± 8528 |
|  | Ratio (5’-DFCR/capecitabine) | 17.4 ± 7.3 | 2.0 ± 0.6*** | 8.8 ± 3.6**^###^ | 4.0 ± 1.0***##^^ |
|  | 5’-DFUR | 1750 ± 637 | 3447 ± 1643 | 2628 ± 793 | 2635 ± 1118 |
|  | Ratio (5’-DFUR/capecitabine) | 1.60 ± 0.96 | 0.21 ± 0.09*** | 0.66 ± 0.37*## | 0.44 ± 0.12***# |
|  | 5-FU | 747 ± 184 | 1525 ± 765 | 2141 ± 372** | 1422 ± 574 |
|  | Ratio (5-FU/Capecitabine) | 0.65 ± 0.31 | 0.09 ± 0.03*** | 0.52 ± 0.25^###^ | 0.24 ± 0.07***^###^^ |
|  | FBAL | 55840 ± 13861 | 59839 ± 11722 | 102959 ± 17203***^###^ | 77125 ± 16534* |
|  | Ratio (FBAL /Capecitabine) | 48 ± 19 | 4.2 ± 2.7*** | 25 ± 11^###^ | 18 ± 16**^###^ |
| C_SI_  ng/g | Capecitabine | 1631 ± 865 | 17217 ± 6548*** | 15234 ± 6017*** | 2411 ± 1191^###^^^^ |
|  | 5’-DFCR | 4514 ± 1341 | 5414 ± 1430 | 6224 ± 1826 | 3604 ± 1572 |
|  | Ratio (5’-DFCR/Capecitabine) | 3.20 ± 1.70 | 0.36 ± 0.20*** | 0.45 ± 0.21*** | 1.80 ± 0.88^###^^^^ |
|  | 5’-DFUR | 2119 ± 460 | 5099 ± 1783* | 4632 ± 1545 | 3194 ± 1571 |
|  | Ratio (5’-DFUR/Capecitabine) | 1.50 ± 0.62 | 0.32 ± 0.13*** | 0.33 ± 0.12*** | 1.50 ± 0.56^###^^^^ |
|  | 5-FU | 3016 ± 835 | 5201 ± 2037 | 4427 ± 1508 | 4125 ± 1844 |
|  | Ratio (5-FU/Capecitabine) | 2.1 ± 0.8 | 0.32 ± 0.10*** | 0.32 ± 0.11*** | 2.0 ± 0.9^###^^^^ |
|  | FBAL | 1174 ± 174 | 1239 ± 335 | 2193 ± 960**## | 1830 ± 655* |
|  | Ratio (FBAL /Capecitabine) | 0.86 ± 0.38 | 0.08 ± 0.05*** | 0.15 ± 0.04*** | 1.5 ± 2.0^###^^^^ |
| C_SIC_  ng/g | Capecitabine | 26460 ± 16294 | 97216 ± 37177** | 88454 ± 25100** | 49736 ± 45851# |
|  | 5’-DFCR | 4408 ± 2348 | 10829 ± 5610 | 8123 ± 2843 | 5180 ± 2406 |
|  | Ratio (5’-DFCR/Capecitabine) | 0.19 ± 0.08 | 0.11 ± 0.04 | 0.10 ± 0.06 | 0.13 ± 0.05 |
|  | 5’-DFUR | 1759 ± 941 | 3905 ± 2357 | 2689 ± 1092 | 2248 ± 905 |
|  | Ratio (5’-DFUR/Capecitabine) | 0.077 ± 0.032 | 0.036 ± 0.016 | 0.035 ± 0.025 | 0.060 ± 0.024 |
|  | 5-FU | 125 ± 90 | 200 ± 109 | 142 ± 64 | 263 ± 149 |
|  | Ratio (x 10^-3^)  (5-FU/Capecitabine) | 5.1 ± 3.4 | 2.1 ± 0.7 | 1.9 ± 1.6* | 6.8 ± 3.0##^^ |
|  | FBAL | N.A. | N.A. | N.A. | N.A. |
|  | Ratio (FBAL /Capecitabine) | N.A. | N.A. | N.A. | N.A. |
| C_colon_  ng/g | Capecitabine | 2249 ± 712 | 14273 ± 9019*** | 8776 ± 1631** | 4966 ± 2983## |
|  | 5’-DFCR | 14904 ± 3800 | 25745 ± 10651 | 28240 ± 5874 | 21166 ± 8096 |
|  | Ratio (5’-DFCR/Capecitabine) | 6.9 ± 1.3 | 2.0 ± 0.4*** | 3.2 ± 0.2***# | 5.1 ± 2.1^###^ |
|  | 5’-DFUR | 2694 ± 755 | 4754 ± 3040 | 3692 ± 793 | 5426 ± 27021.6 |
|  | Ratio (5’-DFUR/Capecitabine) | 1.2 ± 0.3 | 0.34 ± 0.06*** | 0.42 ± 0.06*** | 1.2 ± 0.5^###^^^^ |
|  | 5-FU | 1186 ± 486 | 1829 ± 1013 | 1455 ± 233 | 2103 ± 1022 |
|  | Ratio (5-FU/Capecitabine) | 0.53 ± 0.09 | 0.14 ± 0.03*** | 0.17 ± 0.02*** | 0.50 ± 0.18^###^^^^ |
|  | FBAL | 1322 ± 249 | 1355 ± 281 | 2105 ± 600**## | 2080 ± 446**## |
|  | Ratio (FBAL /Capecitabine) | 0.62 ± 0.16 | 0.12 ± 0.05*** | 0.24 ± 0.02**# | 0.60 ± 0.36^###^^ |

Data are given as mean ± SD (*n* = 6). C_liver_, liver concentration; SI, small intestine (tissue); SIC, small intestine contents; C_testis_, testis concentration; N.A., Not applicable; *, *P* < 0.05; **, *P* < 0.01; ***, *P* < 0.001 compared to wild-type mice; ^#^, *P* < 0.05; ^##^, *P* < 0.01; ^###^, *P* < 0.001 compared to *Ces2^-/-^* mice; ^, *P* < 0.05; ^^, *P* < 0.01; ^^^, *P* < 0.001 for comparison between *Ces2^-/-^*A and *Ces2^-/-^*V mice. Statistical analysis was applied after log-transformation of linear data and compared within either oral administration groups or I.V. injection groups.

Supplemental Table 4. Tissue pharmacokinetic parameters of vinorelbine and its active metabolite deacetylvinorelbine in male wild-type, *Ces2^-/-^, Ces2^-/-^*A and *Ces2^-/-^*V mice over 4 h after oral administration or i.v. injection of 10 mg/kg vinorelbine.

| **Tissue Parameter** | | **Genotype/Groups** | | | | | | | | |
| --- | --- | --- | --- | --- | --- | --- | --- | --- | --- | --- |
|  |  | **Oral administration** | | | |  | **I.V. Injection** | | | |
|  |  | Wild-type | *Ces2^-/-^* | *Ces2^-/-^*A | *Ces2^-/-^*V |  | Wild-type | *Ces2^-/-^* | *Ces2^-/-^*A | *Ces2^-/-^*V |
| C_liver_, ng/g | Vinorelbine | 1530 ± 409 | 8470 ± 8152*** | 8520 ± 3445*** | 8304 ± 2003*** |  | 12240 ± 4958 | 23365 ± 6113** | 24326 ± 3721*** | 24682 ± 4451*** |
|  | Deacetylvinorelbine | 7589 ± 2725 | 82 ± 29*** | 162 ± 52***^##^ | 178 ± 110***^##^ |  | 26773 ± 10517 | 487 ± 127*** | 584 ± 157*** | 513 ± 61*** |
|  | Ratio (x 10^-3^)  (deacetylvinorelbine /vinorelbine) | 5000 ± 1000 | 15 ± 7.5*** | 19 ± 1.7*** | 21 ± 7.8*** |  | 2500 ± 1300 | 21 ± 1.9*** | 25± 7.0*** | 22 ± 6.6*** |
| C_SI_  ng/g | Vinorelbine | 10755 ± 3700 | 18335 ± 4305 | 18081 ± 3649 | 15629 ± 6976 |  | 3466 ± 858 | 5925 ± 1348** | 5604 ± 644* | 7682 ± 2242*** |
|  | Deacetylvinorelbine | 9590 ± 4337 | 625 ± 219*** | 748 ± 225*** | 808 ± 578*** |  | 2214 ± 605 | 87 ± 15*** | 79 ± 13*** | 134 ± 47***^ |
|  | Ratio (x 10^-3^)  (deacetylvinorelbine /vinorelbine) | 880 ± 220 | 34 ± 10.0*** | 39 ± 8.0*** | 49 ± 15*** |  | 630 ± 46 | 15 ± 1.2*** | 14 ± 1.0*** | 17 ± 2.2***^^ |
| C_SIC_  ng/g | Vinorelbine | 76985 ± 29328 | 158738 ± 46695** | 177344 ± 72971** | 113728 ± 19392 |  | 27651 ± 13355 | 40070 ± 25523 | 47236 ± 30865 | 48363 ± 27038 |
|  | Deacetylvinorelbine | 15491 ± 4320 | 1091 ± 405*** | 1120 ± 623*** | 957 ± 441*** |  | 27594 ± 13421 | 291 ± 180*** | 400 ± 315*** | 511 ± 322*** |
|  | Ratio (x 10^-3^) (deacetylvinorelbine /vinorelbine) | 220 ± 73 | 6.7 ± 1.4*** | 5.9 ± 1.6*** | 8.4 ± 3.4*** |  | 1000 ± 270 | 7.4 ± 1.0*** | 8.1 ± 1.1*** | 10 ± 1.0***^##^ |
| C_colon_  ng/g | Vinorelbine | 348 ± 136 | 581 ± 175 | 788 ± 189** | 608 ± 203* |  | 2361 ± 692 | 5625 ± 2998* | 5776 ± 3086* | 6745 ± 1469** |
|  | Deacetylvinorelbine | 369 ± 240 | 5.0 ± 2.1*** | 6.0 ± 2.6*** | 8.1 ± 4.8*** |  | 780 ± 199 | 58 ± 45*** | 42 ± 43*** | 120 ± 45***^ |
|  | Ratio (x 10^-3^) (deacetylvinorelbine /vinorelbine) | 1000 ± 390 | 8.4 ± 1.5*** | 7.8 ± 2.6*** | 13 ± 3.7*** |  | 340 ± 70 | 9.4 ± 3.8*** | 7.0 ± 3.7*** | 17 ± 4.7***^^ |

Data are given as mean ± S.D. (n = 6). C_liver_, liver concentration; SI, small intestine (tissue); SIC, small intestine contents; C_testis_, testis concentration; *, *P* < 0.05; **, *P* < 0.01; ***, *P* < 0.001 compared to wild-type mice; ^#^, *P* < 0.05; ^##^, *P* < 0.01; ^###^, *P* < 0.001 compared to *Ces2^-/-^* mice; ^, *P* < 0.05; ^^, *P* < 0.01; ^^^, *P* < 0.001 for comparison between *Ces2^-/-^*A and *Ces2^-/-^*V mice. Statistical analysis was applied after log-transformation of linear data and compared within either oral administration groups or I.V. injection groups.
