# Supplementary figures and images for: Intestinal human carboxylesterase 2 (CES2) expression rescues drug metabolism and most metabolic syndrome phenotypes in global Ces2 cluster knockout mice
Source: Acta Pharmacol Sin. 2024 Nov 4;46(3):777–93. doi: 10.1038/s41401-024-01407-4 (PMC11845761; doi:10.1038/s41401-024-01407-4)

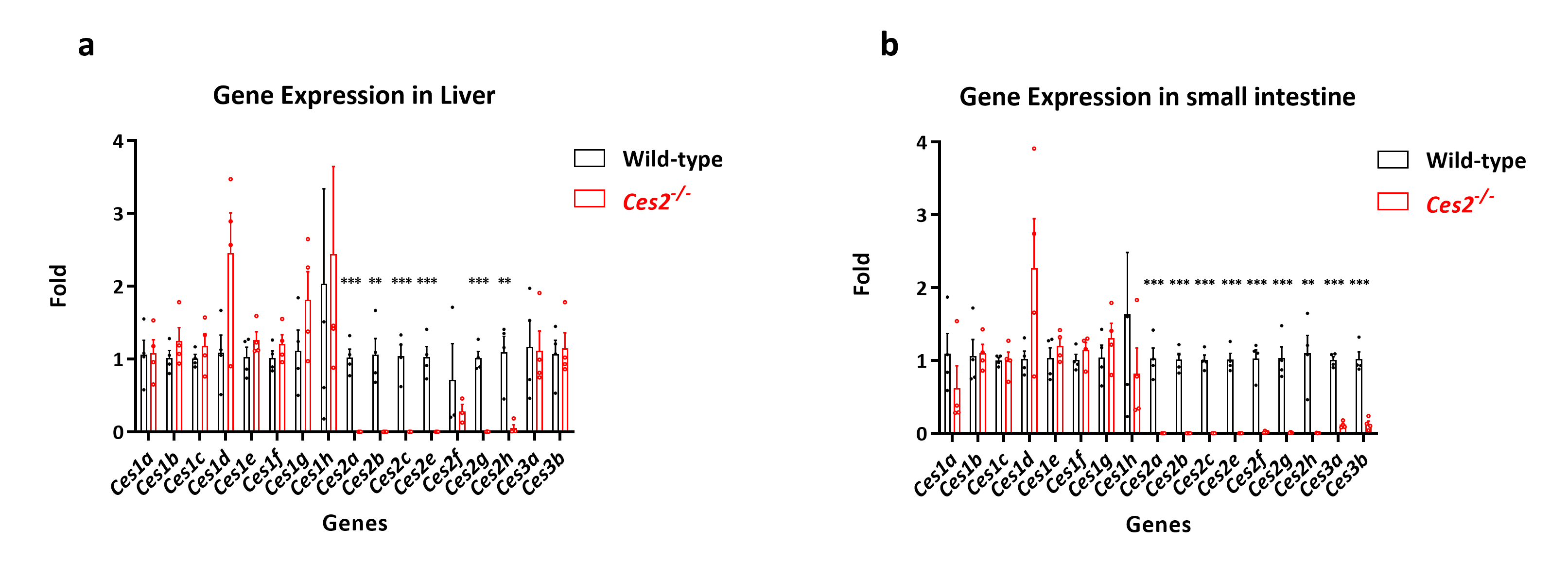

Supplement: Supplementary file 3 — Supplementary Fig. S1 [file 41401_2024_1407_MOESM3_ESM.tif]

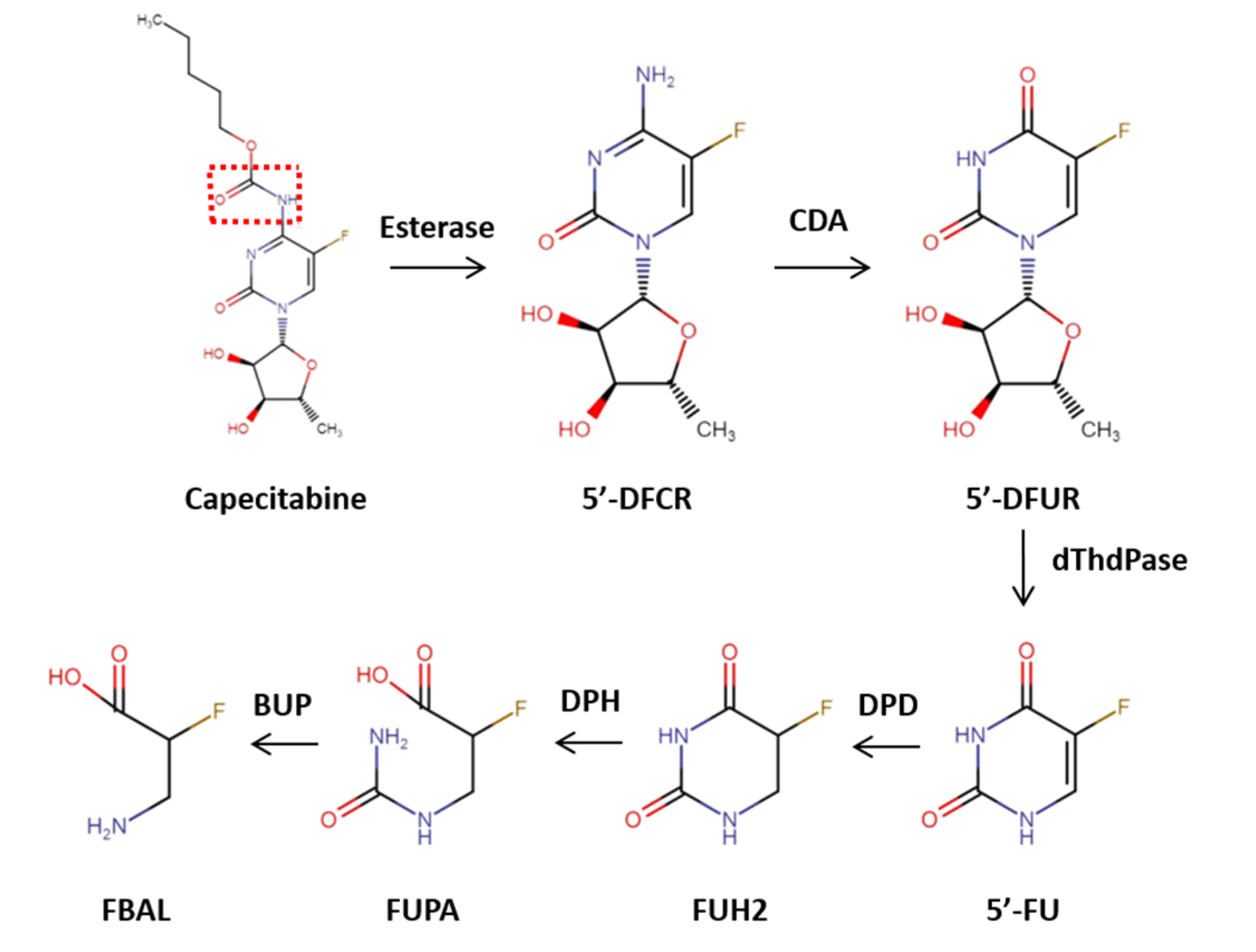

Supplement: Supplementary file 4 — Supplementary Fig. S2 [file 41401_2024_1407_MOESM4_ESM.tif]

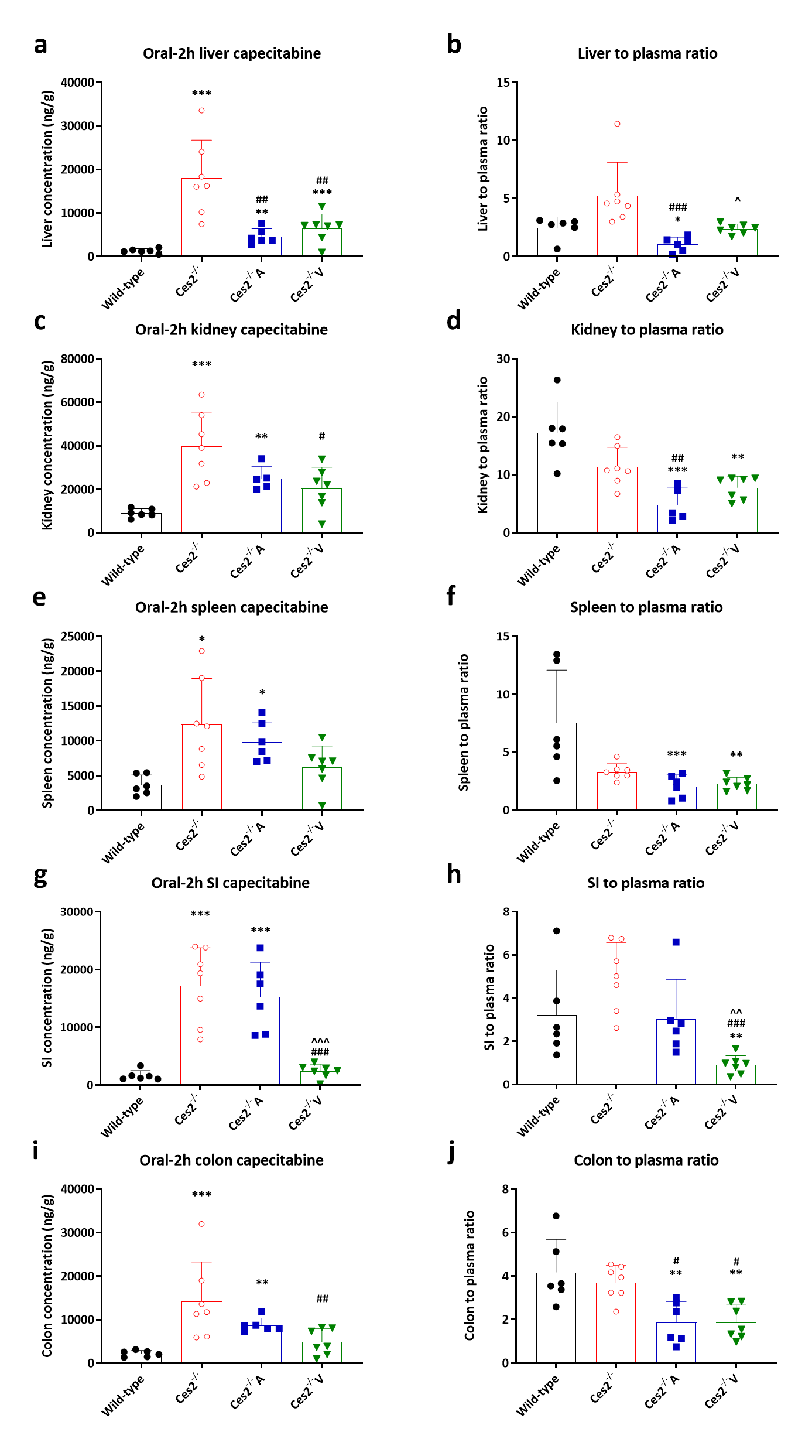

Supplement: Supplementary file 5 — Supplementary Fig. S3 [file 41401_2024_1407_MOESM5_ESM.tif]

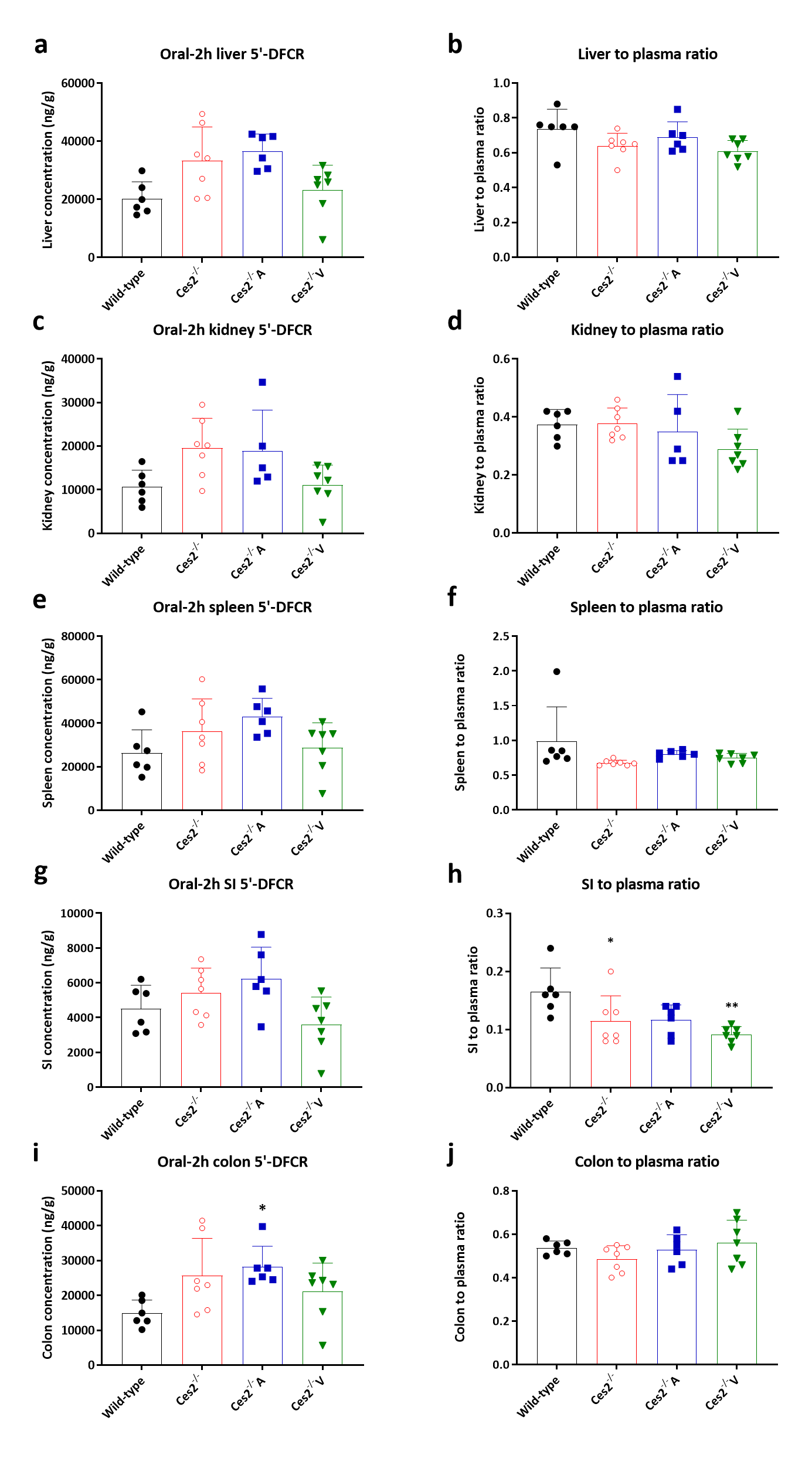

Supplement: Supplementary file 6 — Supplementary Fig. S4 [file 41401_2024_1407_MOESM6_ESM.tif]

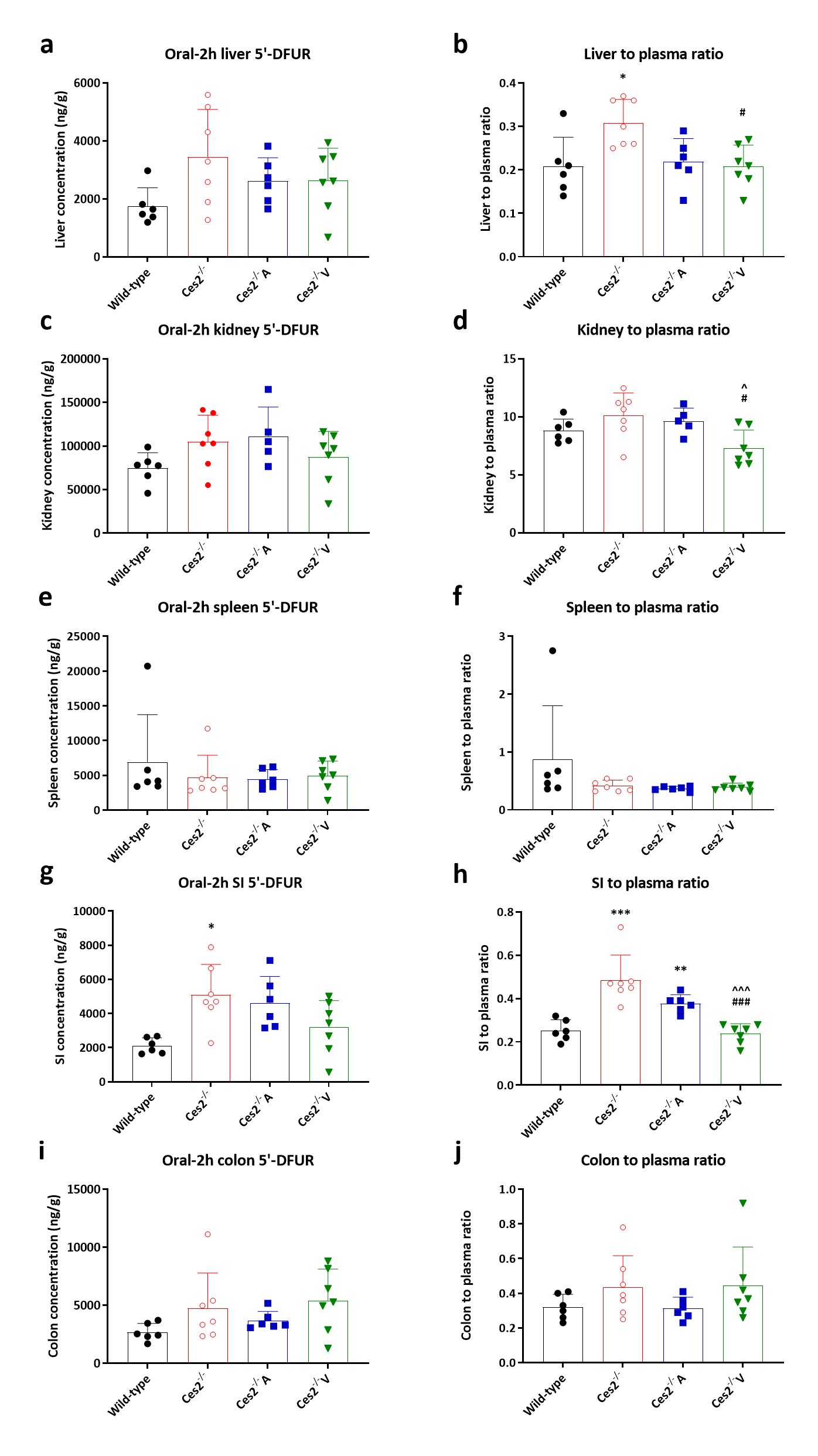

Supplement: Supplementary file 7 — Supplementary Fig. S5 [file 41401_2024_1407_MOESM7_ESM.tif]

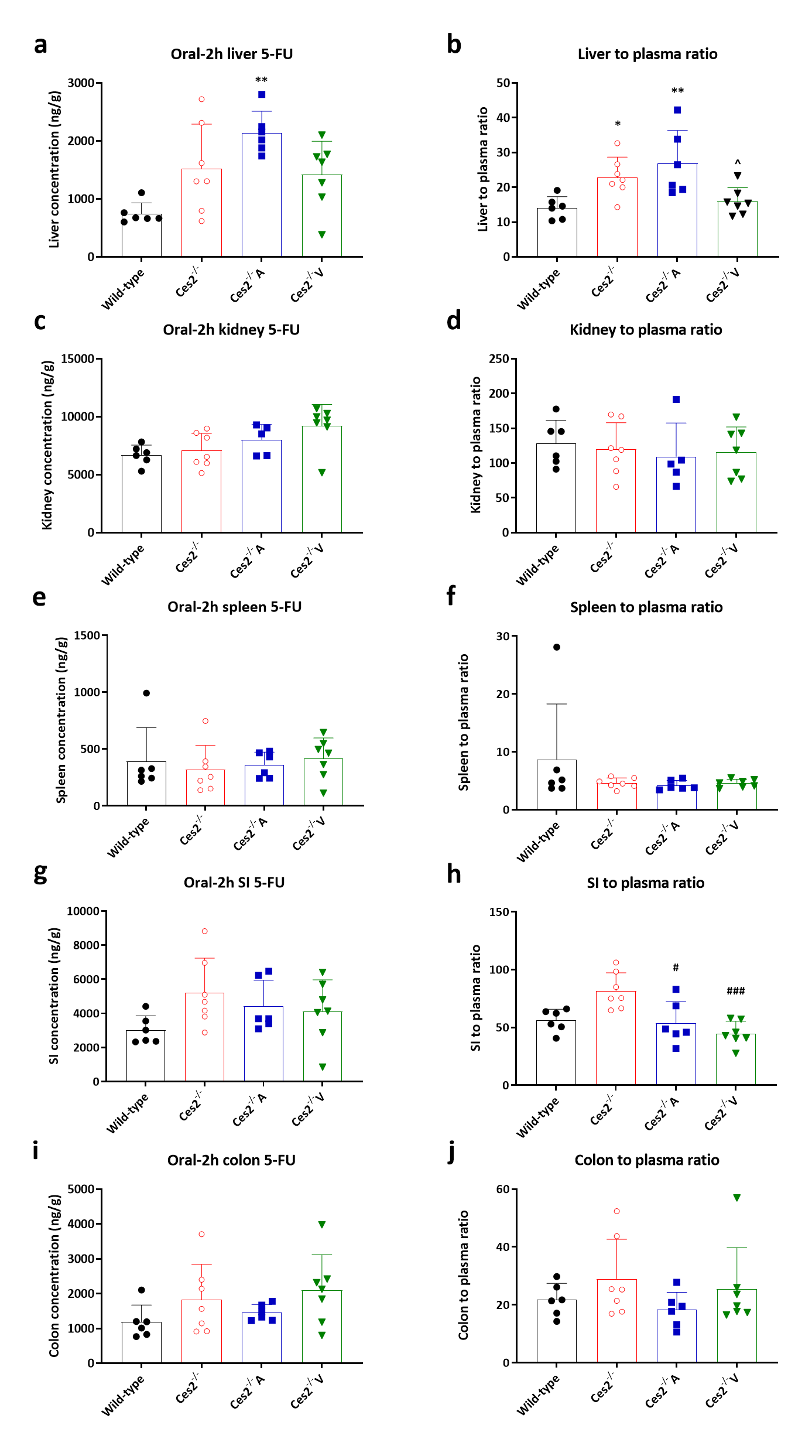

Supplement: Supplementary file 8 — Supplementary Fig. S6 [file 41401_2024_1407_MOESM8_ESM.tif]

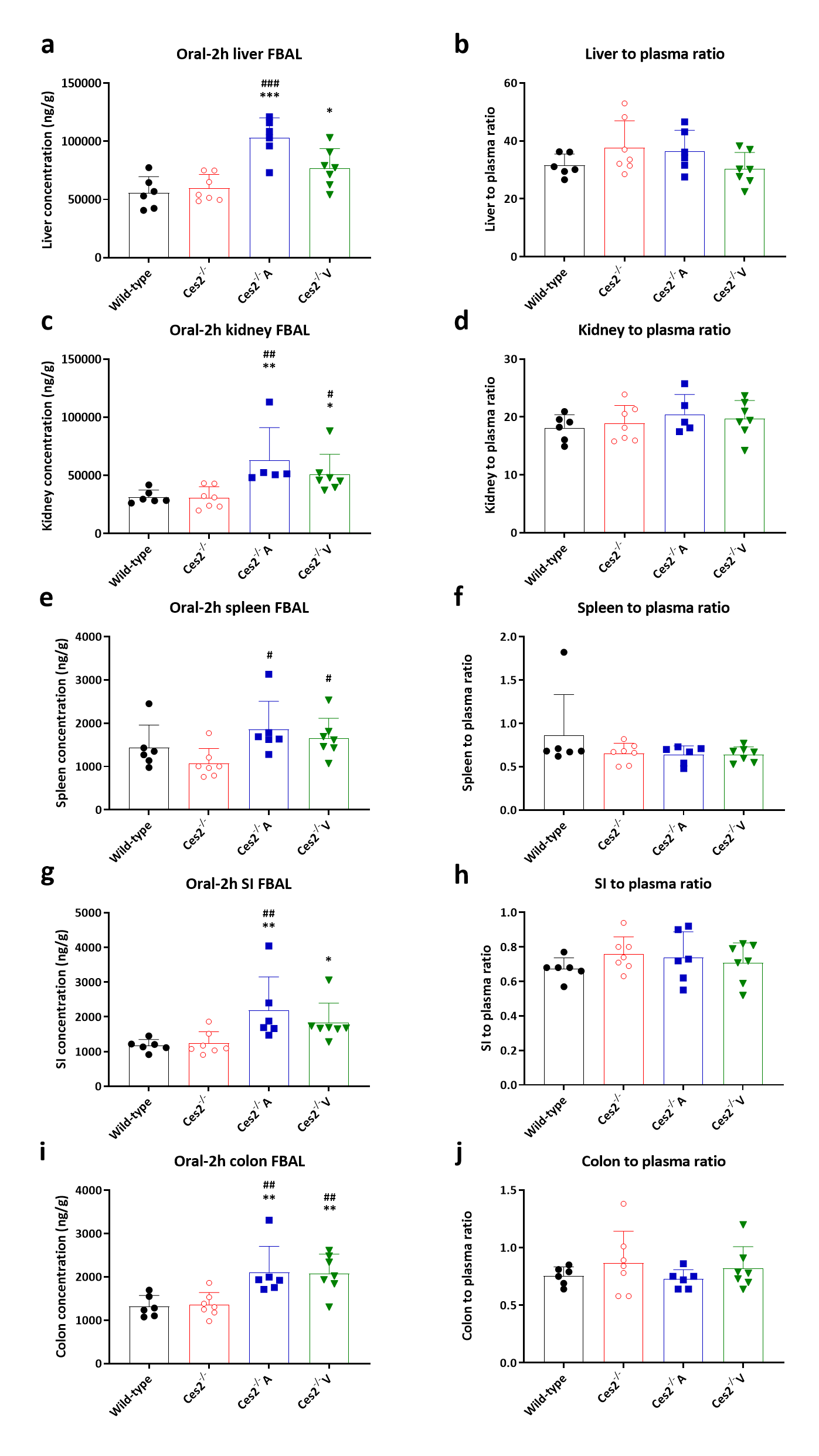

Supplement: Supplementary file 9 — Supplementary Fig. S7 [file 41401_2024_1407_MOESM9_ESM.tif]

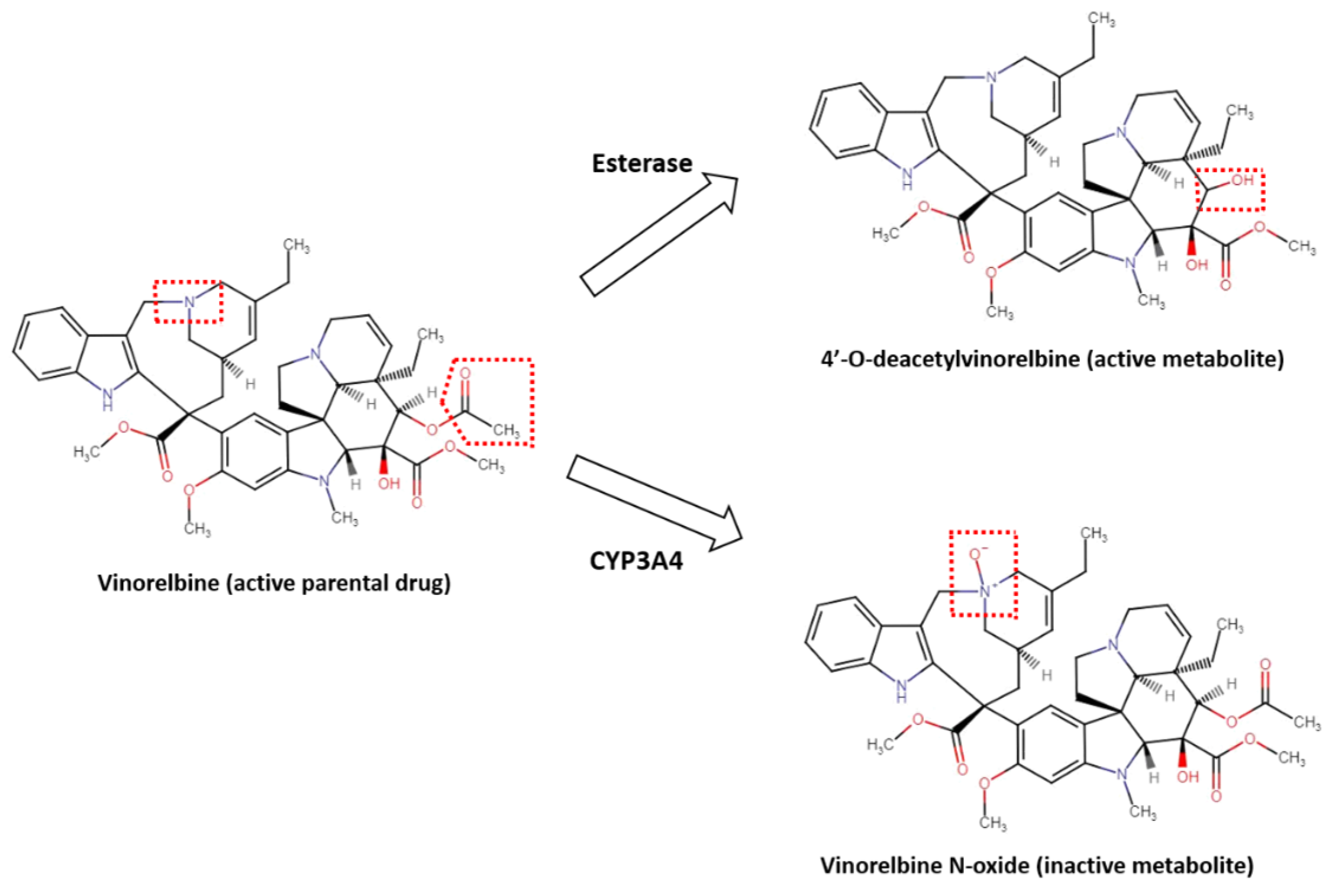

Supplement: Supplementary file 10 — Supplementary Fig. S8 [file 41401_2024_1407_MOESM10_ESM.tif]

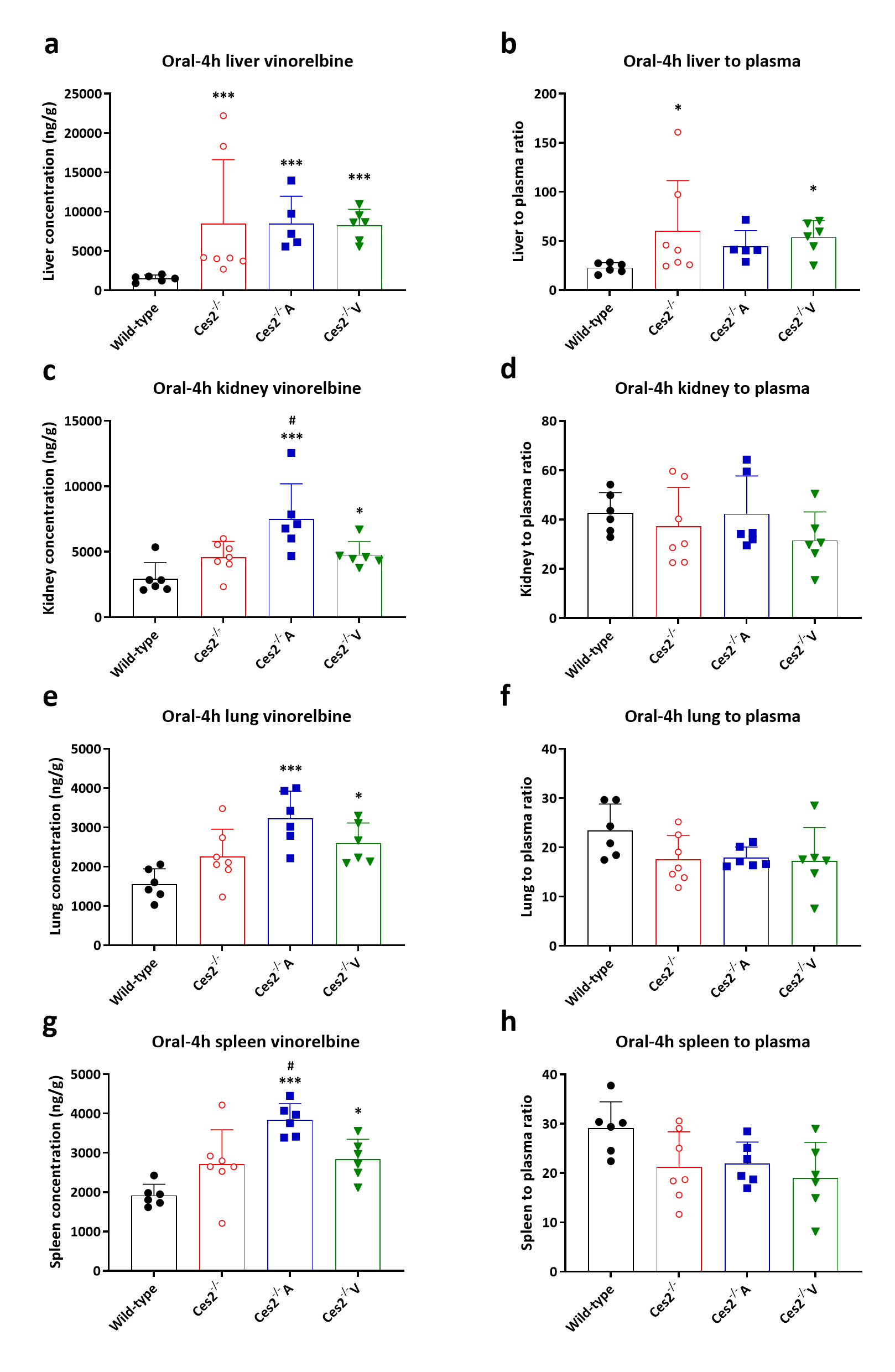

Supplement: Supplementary file 11 — Supplementary Fig. S9 [file 41401_2024_1407_MOESM11_ESM.tif]

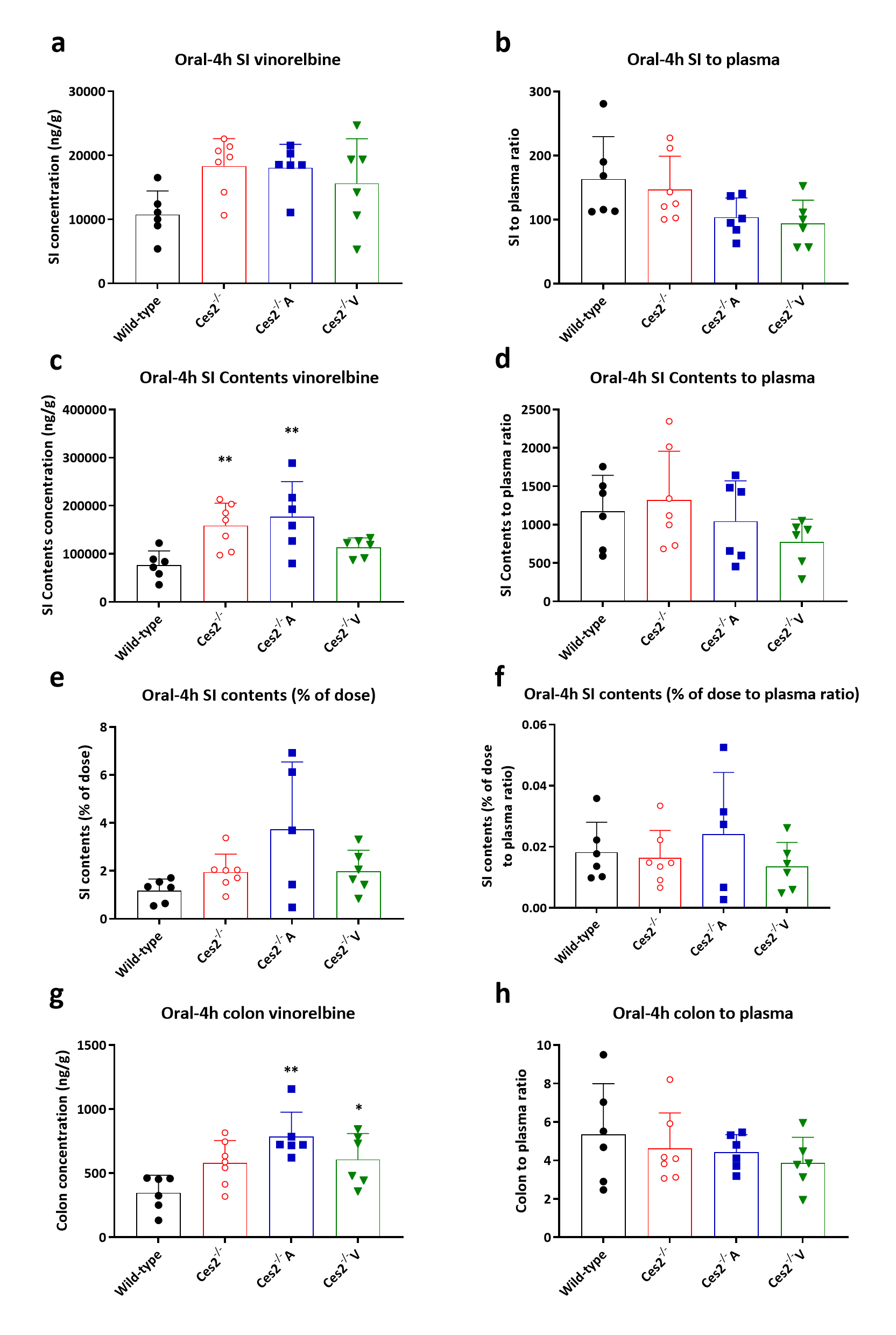

Supplement: Supplementary file 12 — Supplementary Fig. S10 [file 41401_2024_1407_MOESM12_ESM.tif]

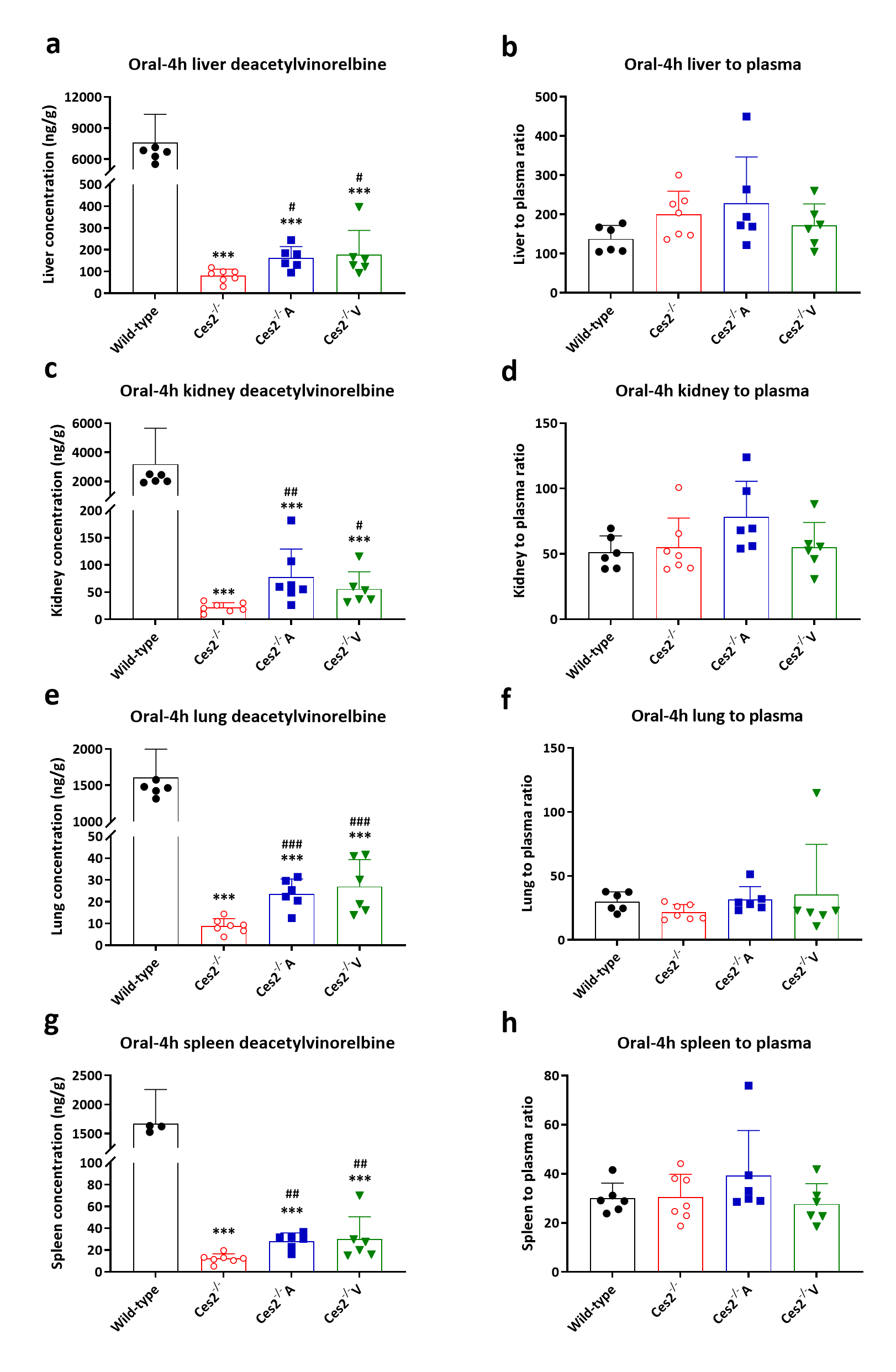

Supplement: Supplementary file 13 — Supplementary Fig. S11 [file 41401_2024_1407_MOESM13_ESM.tif]

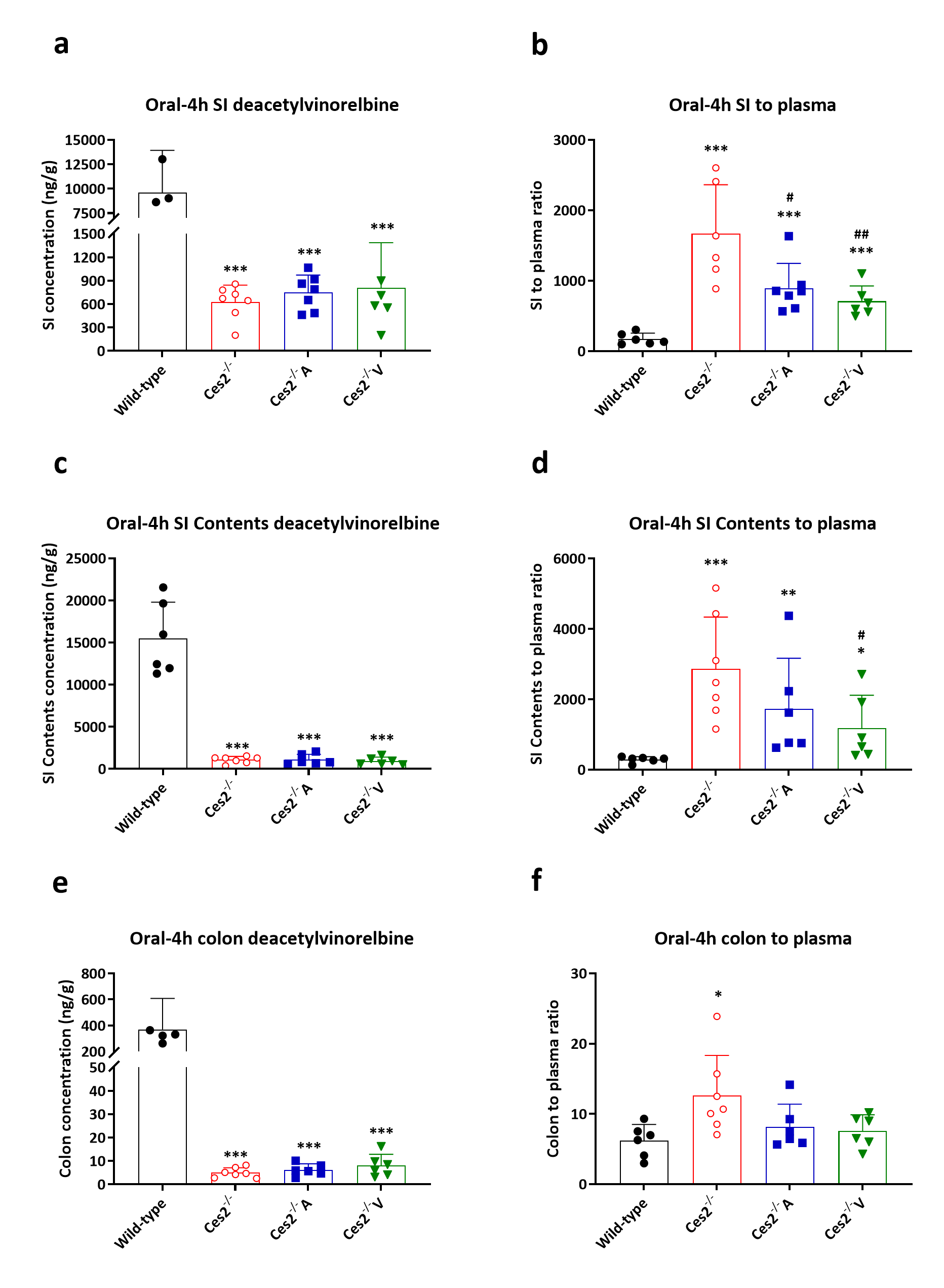

Supplement: Supplementary file 14 — Supplementary Fig. S12 [file 41401_2024_1407_MOESM14_ESM.tif]

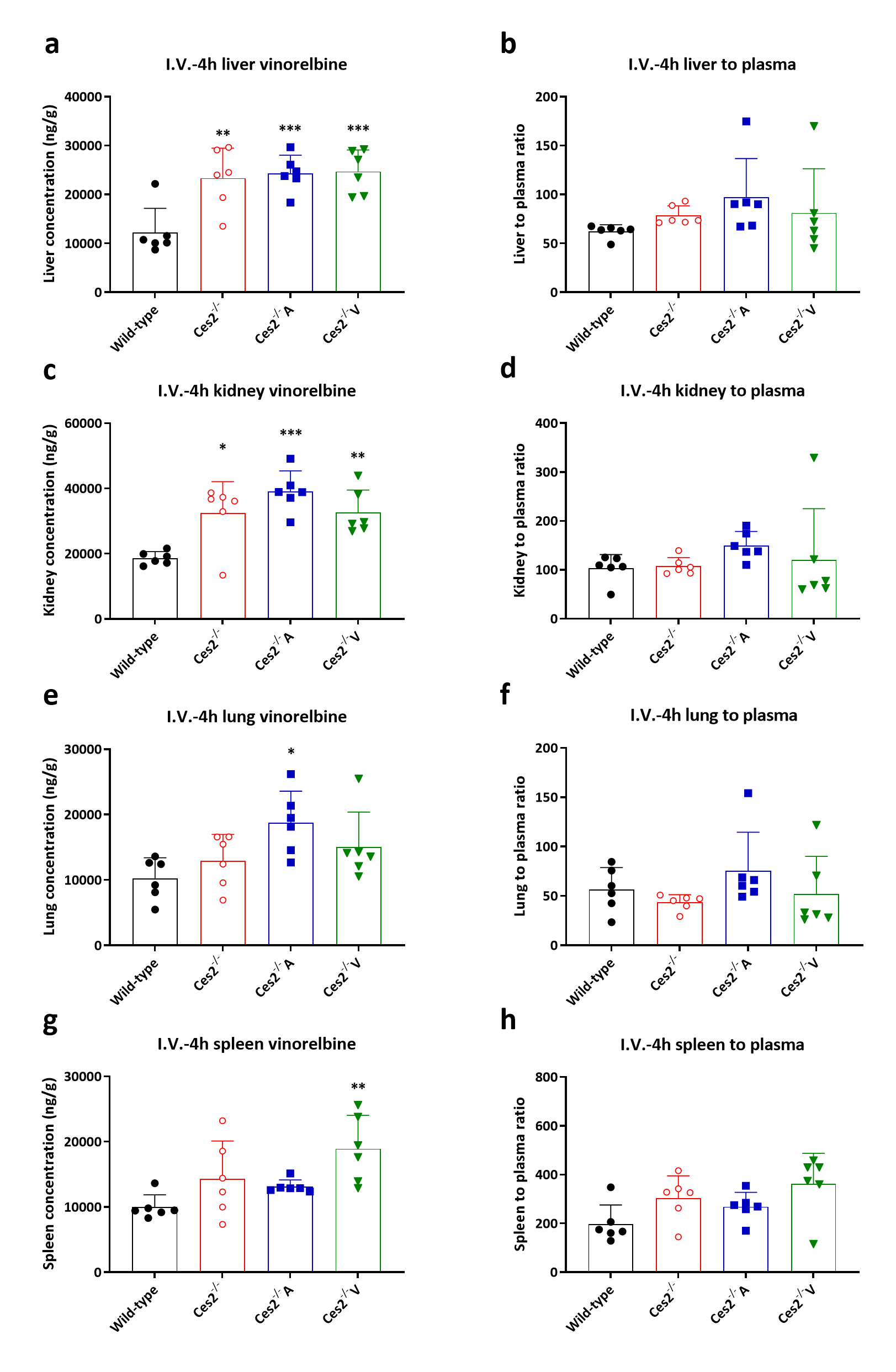

Supplement: Supplementary file 15 — Supplementary Fig. S13 [file 41401_2024_1407_MOESM15_ESM.tif]

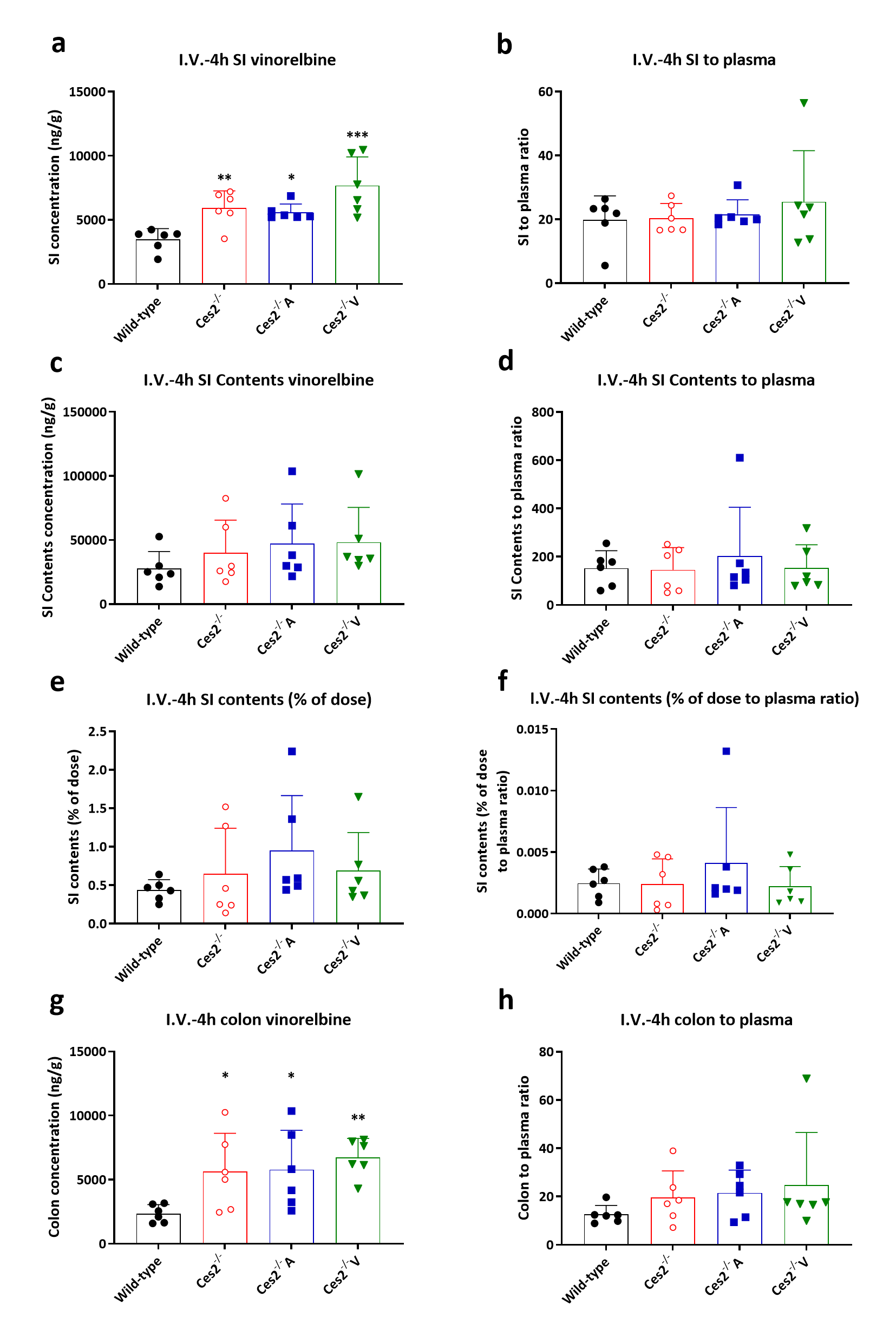

Supplement: Supplementary file 16 — Supplementary Fig. S14 [file 41401_2024_1407_MOESM16_ESM.tif]

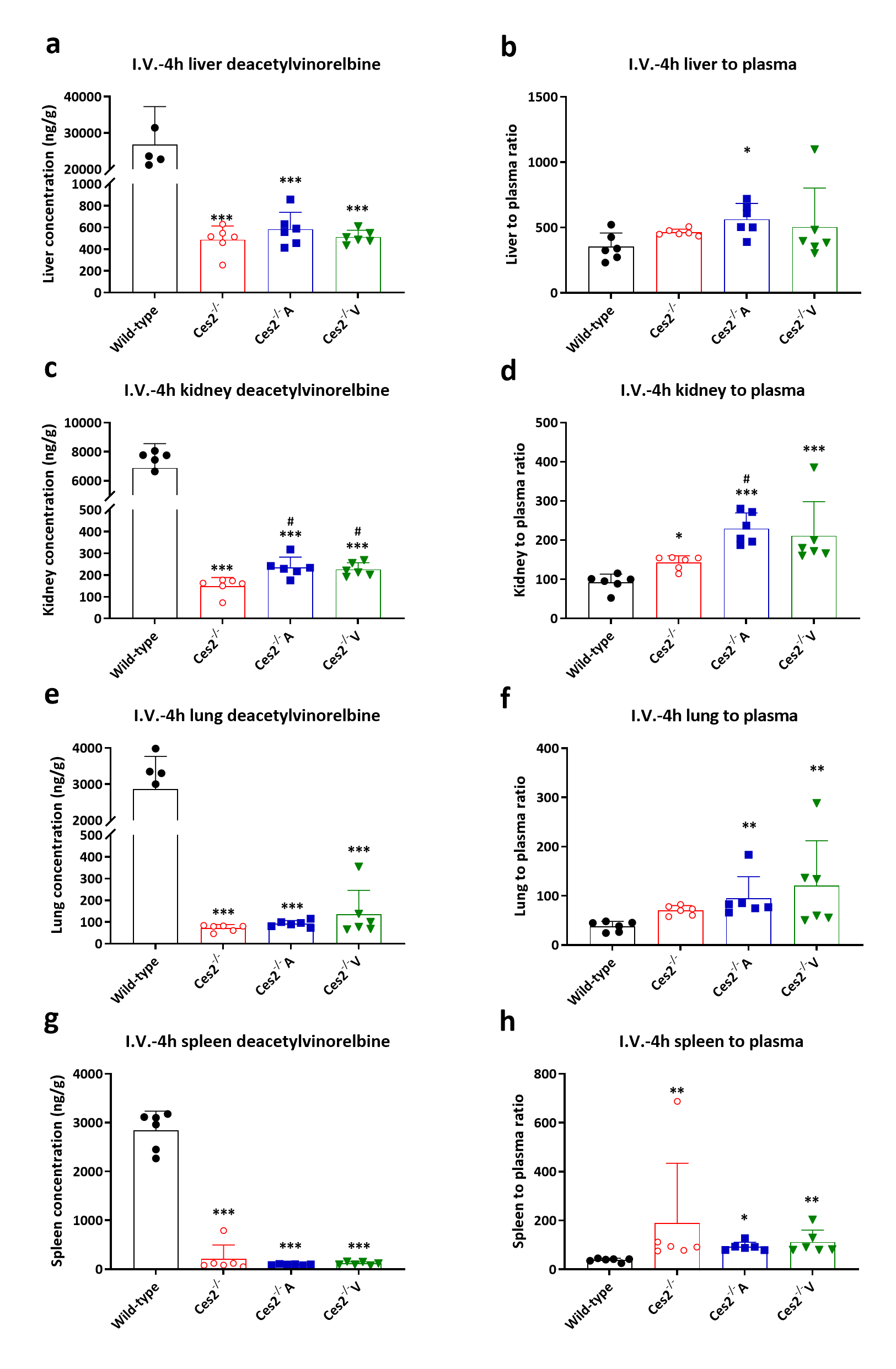

Supplement: Supplementary file 17 — Supplementary Fig. S15 [file 41401_2024_1407_MOESM17_ESM.tif]

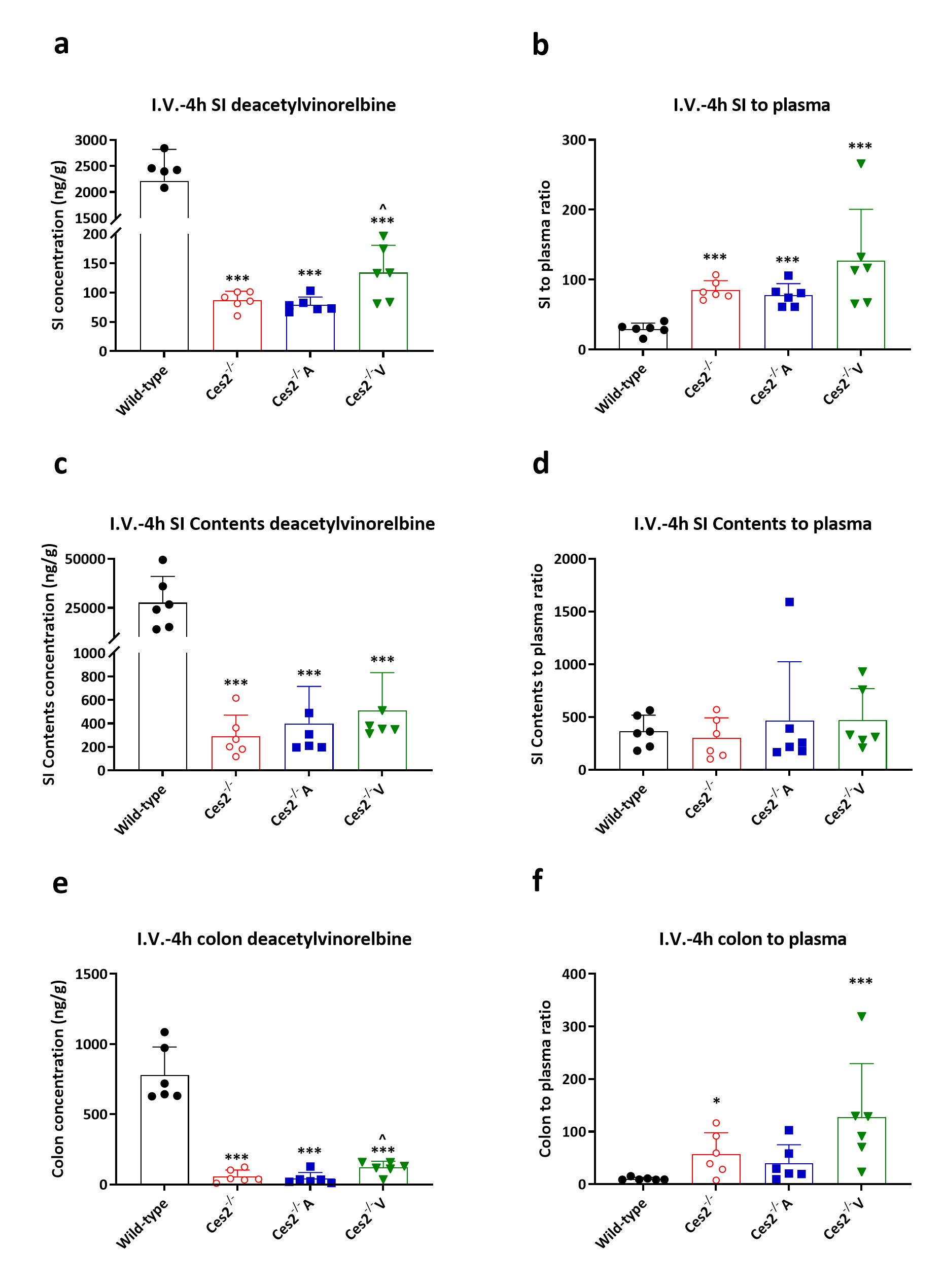

Supplement: Supplementary file 18 — Supplementary Fig. S16 [file 41401_2024_1407_MOESM18_ESM.tif]

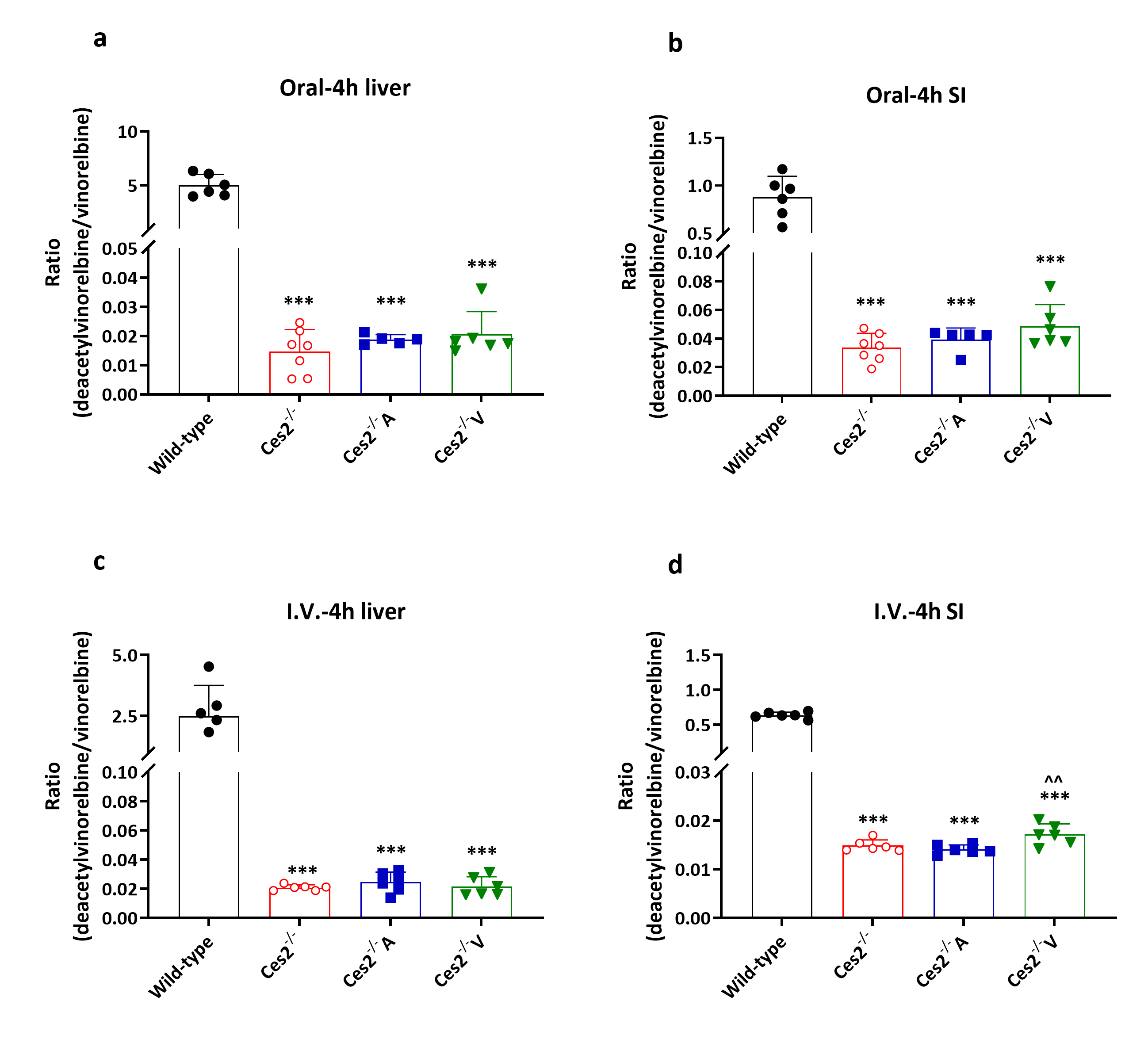

Supplement: Supplementary file 19 — Supplementary Fig. S17 [file 41401_2024_1407_MOESM19_ESM.tif]

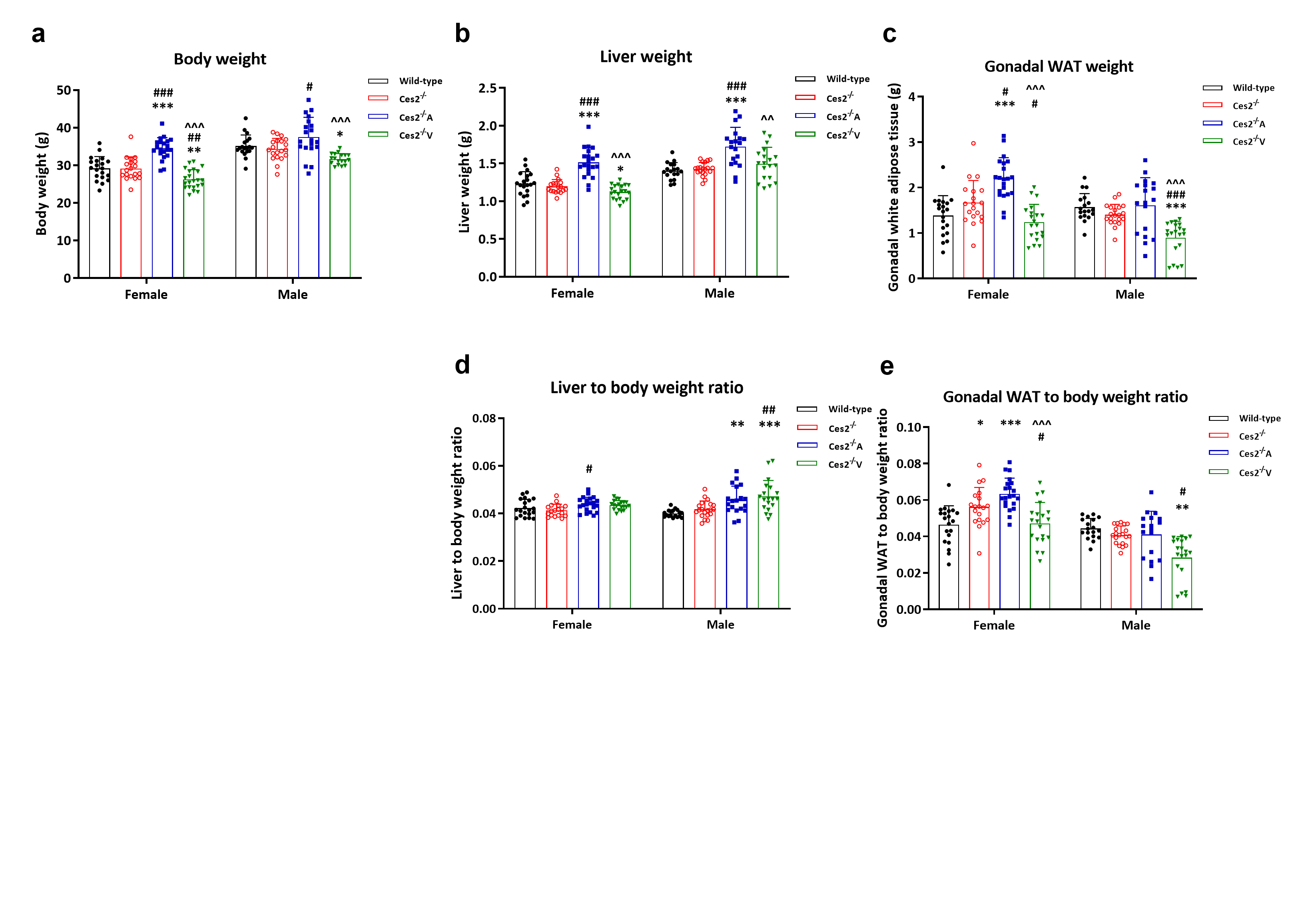

Supplement: Supplementary file 20 — Supplementary Fig. S18 [file 41401_2024_1407_MOESM20_ESM.tif]

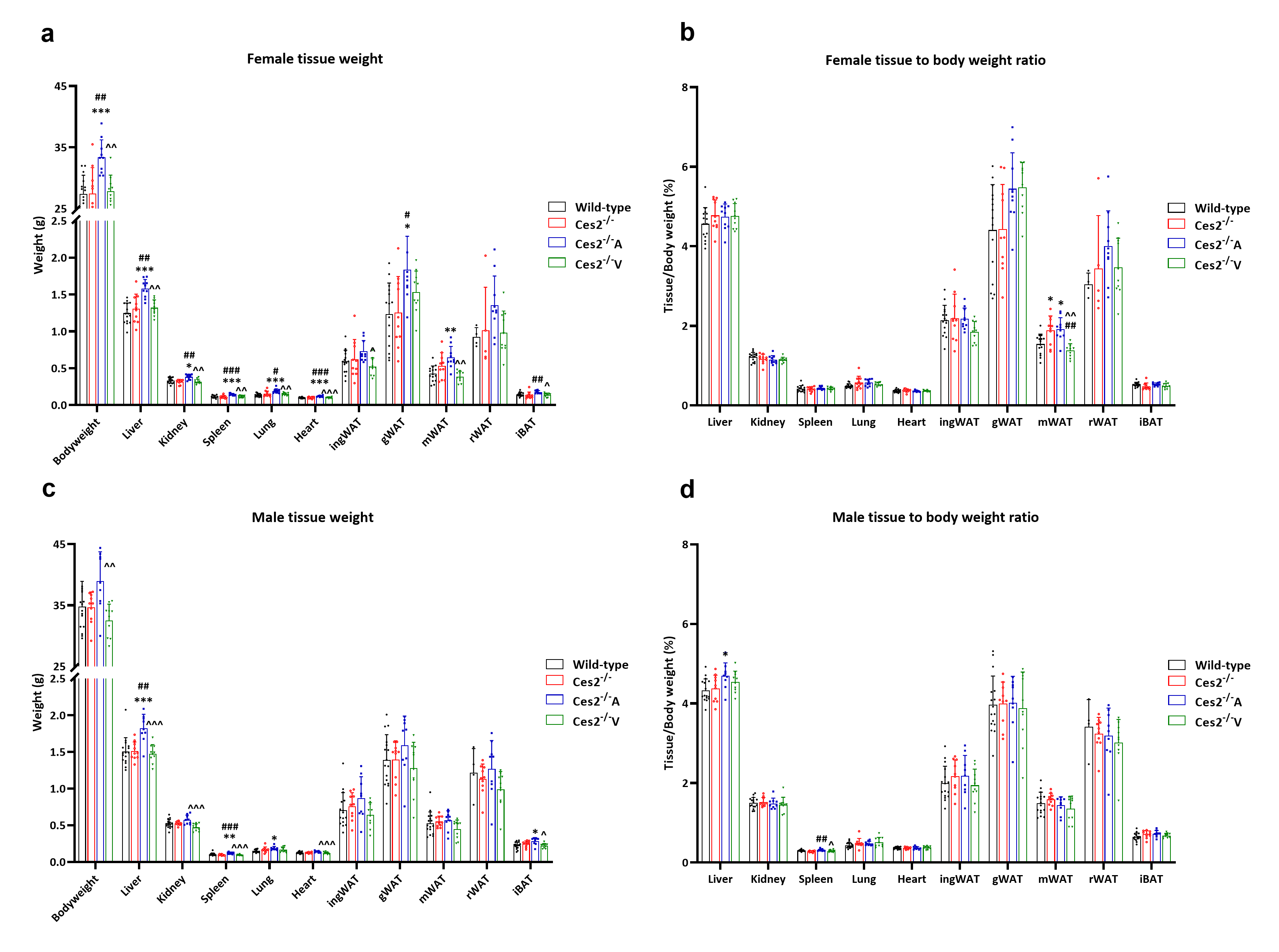

Supplement: Supplementary file 21 — Supplementary Fig. S19 [file 41401_2024_1407_MOESM21_ESM.tif]

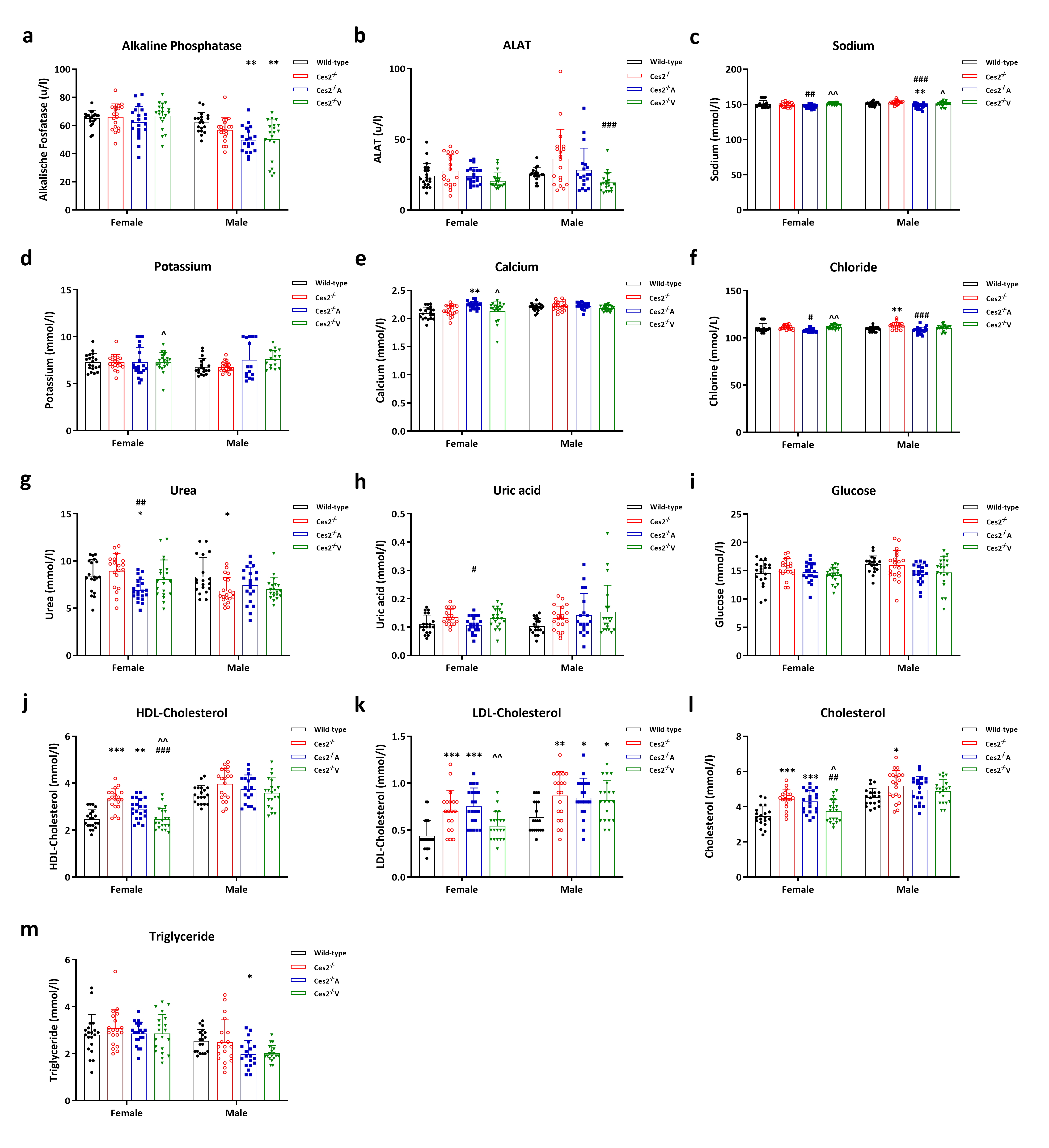

Supplement: Supplementary file 22 — Supplementary Fig. S20 [file 41401_2024_1407_MOESM22_ESM.tif]

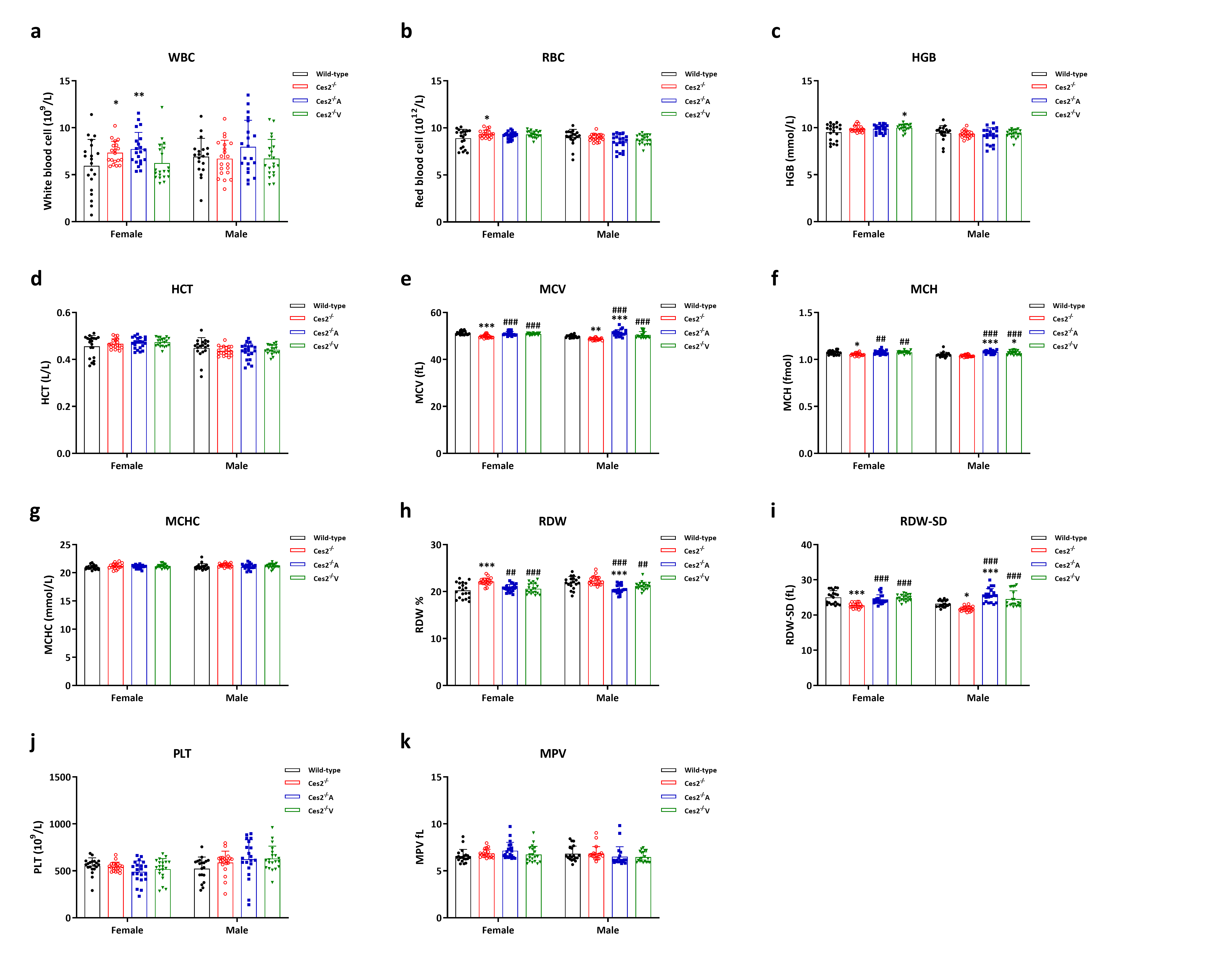

Supplement: Supplementary file 23 — Supplementary Fig. S21 [file 41401_2024_1407_MOESM23_ESM.tif]

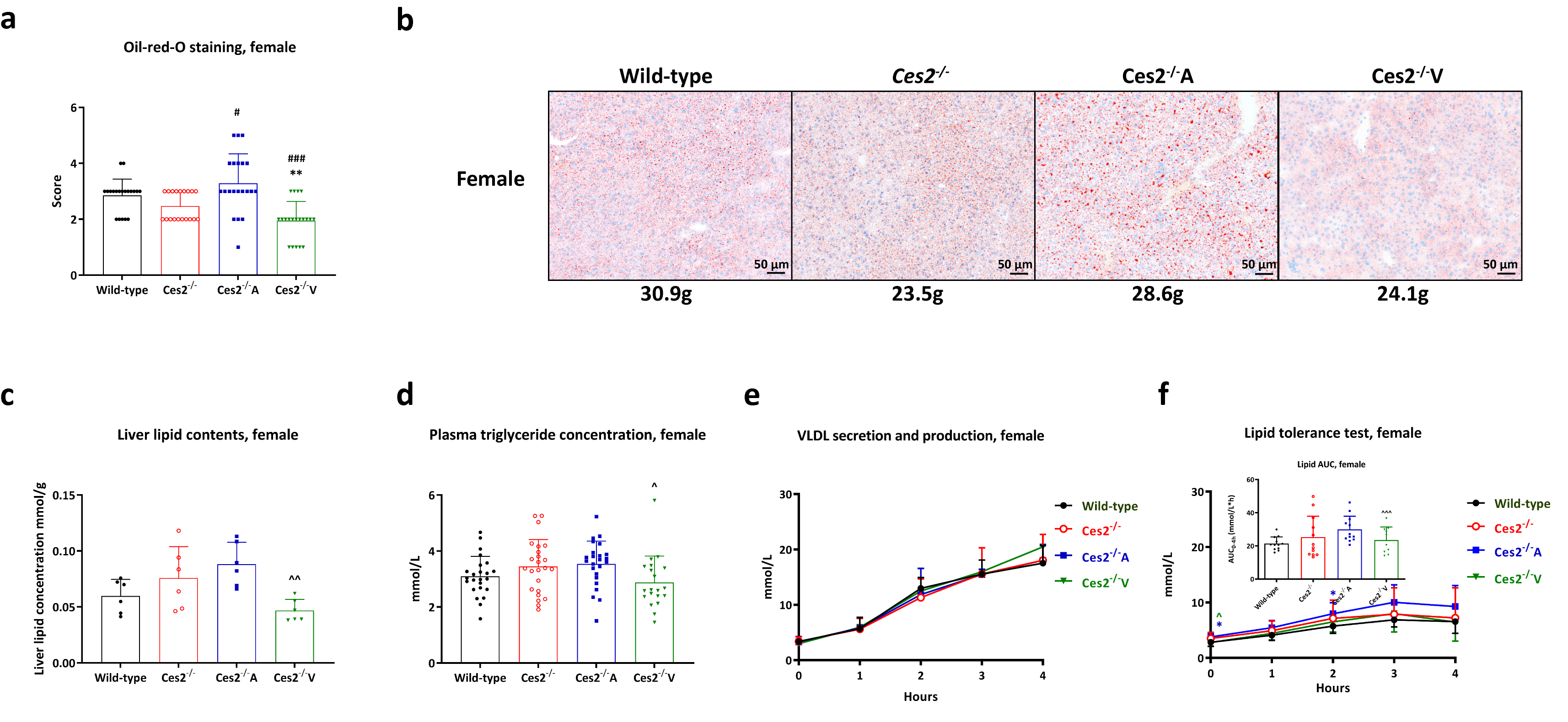

Supplement: Supplementary file 24 — Supplementary Fig. S22 [file 41401_2024_1407_MOESM24_ESM.tif]

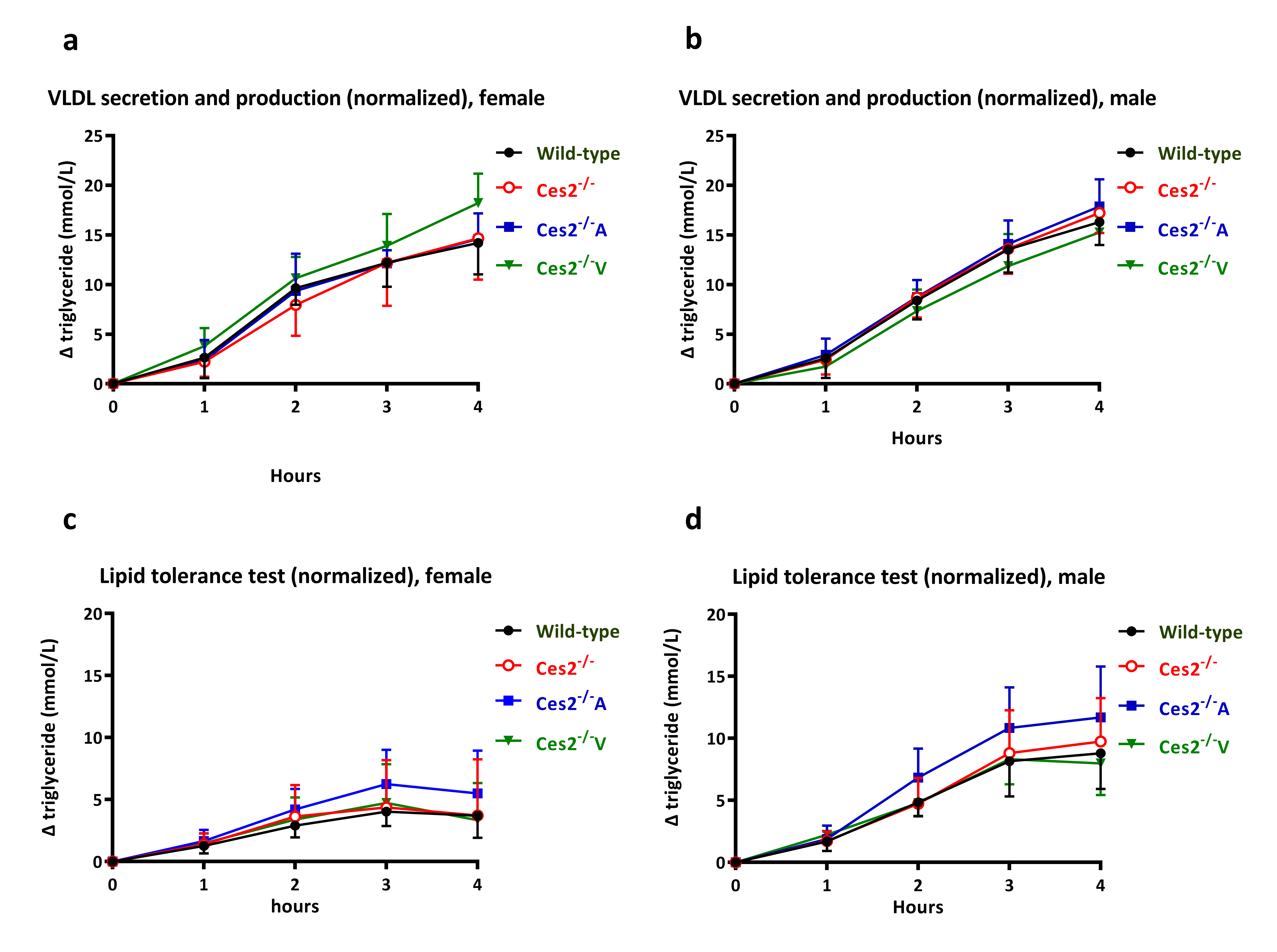

Supplement: Supplementary file 25 — Supplementary Fig. S23 [file 41401_2024_1407_MOESM25_ESM.tif]

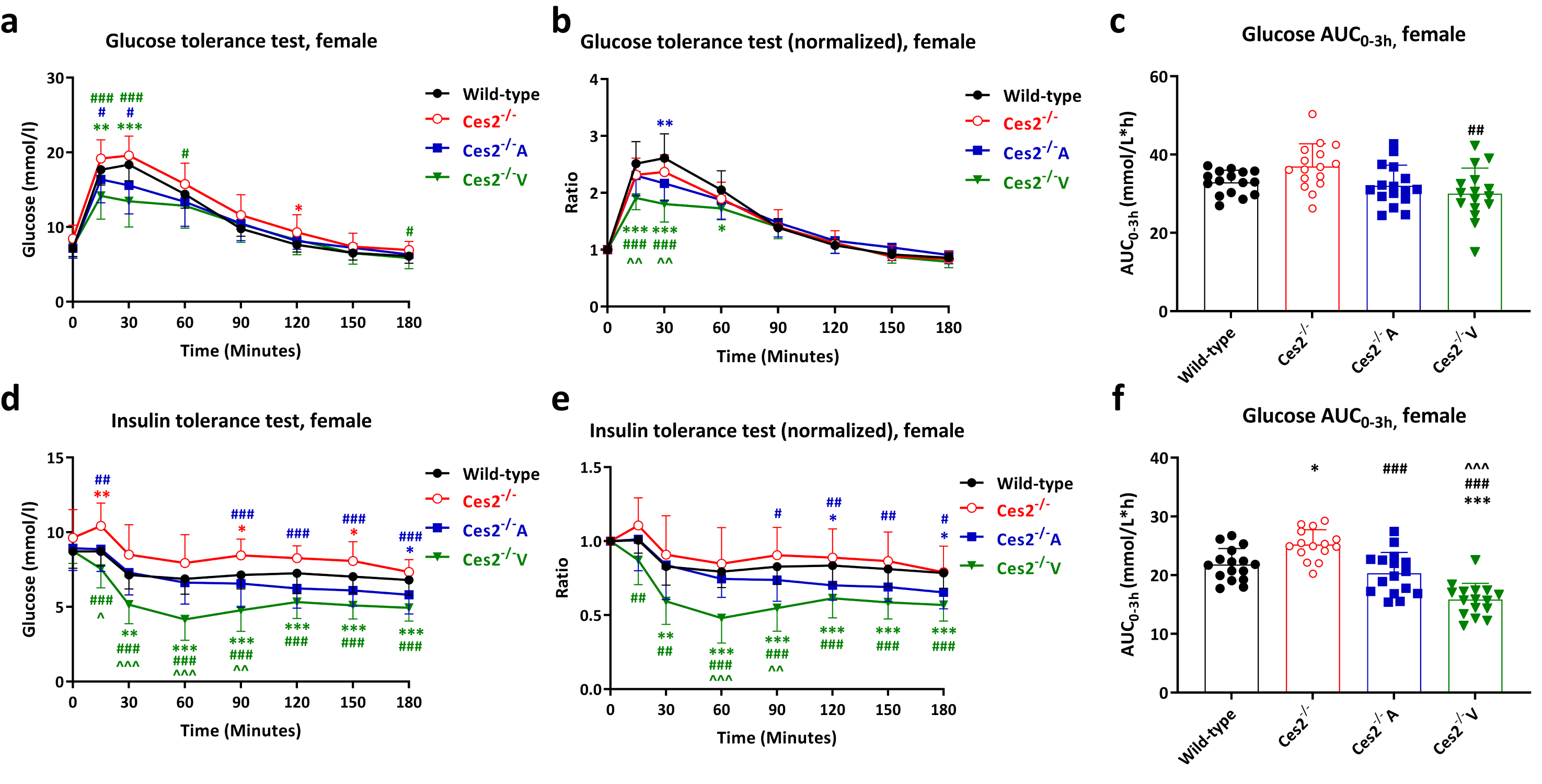

Supplement: Supplementary file 26 — Supplementary Fig. S24 [file 41401_2024_1407_MOESM26_ESM.tif]
