# Supplementary material for: Intestinal human carboxylesterase 2 (CES2) expression rescues drug metabolism and most metabolic syndrome phenotypes in global Ces2 cluster knockout mice
Source: Acta Pharmacol Sin. 2024 Nov 4;46(3):777–93. doi: 10.1038/s41401-024-01407-4 (PMC11845761; doi:10.1038/s41401-024-01407-4)
Supplement: Supplementary file 27 — Supplementary Figure Legend [file 41401_2024_1407_MOESM27_ESM.docx]

Supplementary Fig. S1 Real-time PCR analysis of related carboxylesterase genes (*Ces1*, *Ces2*, and *Ces3*) for wild-type and *Ces2^-/-^* mice in liver (a) and small intestine (SI) (b) tissues. Data (linear expression level normalized to the wild-type RNA level for each gene) are given as mean ± SD (*n* = 4). Student’s *t*-test was used for statistical analysis. *, *P* < 0.05; **, *P* < 0.01; ***, *P* < 0.001 compared to wild-type mice.

Supplementary Fig. S2 Chemical structure and the bio-activation process of capecitabine to 5’-DFCR, 5’-DFUR, 5-FU and FBAL. The red frame indicates where esterases effectively cleave capecitabine. CDA, cytidine deaminase; dThdPase, thymidine phosphorylase; DPD, dihydropyrimidine dehydrogenase; DPH, dihydropyrimidinase; BUP, b-alanine synthase.

Supplementary Fig. S3 Liver, kidney, spleen, small intestine (SI) and colon capecitabine concentrations (a, c, e, g and i), and capecitabine tissue-to-plasma concentration ratios (b, d, f, h and j) in female wild-type, *Ces2^-/-^*, *Ces2^-/-^*A and *Ces2^-/-^*V mice over 2 h after oral administration of 500 mg/kg capecitabine. Data are given as mean ± SD (*n* = 5 - 7). *, *P* < 0.05; **, *P* < 0.01; ***, *P* < 0.001 compared to wild-type mice. ^#^, *P* < 0.05; ^##^, *P* < 0.01; ^###^, *P* < 0.001 compared to *Ces2^-/-^* mice. ^, *P* < 0.05; ^^, *P* < 0.01; ^^^, *P* < 0.001 for comparison between *Ces2^-/-^*A and *Ces2^-/-^*V mice. Statistical analysis was applied after log-transformation of linear data.

Supplementary Fig. S4 Liver, kidney, spleen, small intestine (SI) and colon 5’-DFCR concentrations (a, c, e, g and i), and 5’-DFCR tissue-to-plasma concentration ratios (b, d, f, h and j) in female wild-type, *Ces2^-/-^*, *Ces2^-/-^*A and *Ces2^-/-^*V mice over 2 h after oral administration of 500 mg/kg capecitabine. Data are given as mean ± SD (*n* = 5 - 7). *, *P* < 0.05; **, *P* < 0.01; ***, *P* < 0.001 compared to wild-type mice. ^#^, *P* < 0.05; ^##^, *P* < 0.01; ^###^, *P* < 0.001 compared to *Ces2^-/-^* mice. ^, *P* < 0.05; ^^, *P* < 0.01; ^^^, *P* < 0.001 for comparison between *Ces2^-/-^*A and *Ces2^-/-^*V mice. Statistical analysis was applied after log-transformation of linear data.

Supplementary Fig. S5 Liver, kidney, spleen, small intestine (SI) and colon 5’-DFUR concentrations (a, c, e, g and i), and 5’-DFUR tissue-to-plasma concentration ratios (b, d, f, h and j) in female wild-type, *Ces2^-/-^*, *Ces2^-/-^*A and *Ces2^-/-^*V mice over 2 h after oral administration of 500 mg/kg capecitabine. Data are given as mean ± SD (*n* = 5 - 7). *, *P* < 0.05; **, *P* < 0.01; ***, *P* < 0.001 compared to wild-type mice. ^#^, *P* < 0.05; ^##^, *P* < 0.01; ^###^, *P* < 0.001 compared to *Ces2^-/-^* mice. ^, *P* < 0.05; ^^, *P* < 0.01; ^^^, *P* < 0.001 for comparison between *Ces2^-/-^*A and *Ces2^-/-^*V mice. Statistical analysis was applied after log-transformation of linear data.

Supplementary Fig. S6 Liver, kidney, spleen, small intestine (SI) and colon 5-FU concentrations (a, c, e, g and i), and 5-FU tissue-to-plasma concentration ratios (b, d, f, h and j) in female wild-type, *Ces2^-/-^*, *Ces2^-/-^*A and *Ces2^-/-^*V mice over 2 h after oral administration of 500 mg/kg capecitabine. Data are given as mean ± SD (*n* = 5 - 7). *, *P* < 0.05; **, *P* < 0.01; ***, *P* < 0.001 compared to wild-type mice. ^#^, *P* < 0.05; ^##^, *P* < 0.01; ^###^, *P* < 0.001 compared to *Ces2^-/-^* mice. ^, *P* < 0.05; ^^, *P* < 0.01; ^^^, *P* < 0.001 for comparison between *Ces2^-/-^*A and *Ces2^-/-^*V mice. Statistical analysis was applied after log-transformation of linear data.

Supplementary Fig. S7 Liver, kidney, spleen, small intestine (SI) and colon FBAL concentrations (a, c, e, g and i), and FBAL tissue-to-plasma concentration ratios (b, d, f, h and j) in female wild-type, *Ces2^-/-^*, *Ces2^-/-^*A and *Ces2^-/-^*V mice over 2 h after oral administration of 500 mg/kg capecitabine. Data are given as mean ± SD (*n* = 5 - 7). *, *P* < 0.05; **, *P* < 0.01; ***, *P* < 0.001 compared to wild-type mice. ^#^, *P* < 0.05; ^##^, *P* < 0.01; ^###^, *P* < 0.001 compared to *Ces2^-/-^* mice. ^, *P* < 0.05; ^^, *P* < 0.01; ^^^, *P* < 0.001 for comparison between *Ces2^-/-^*A and *Ces2^-/-^*V mice. Statistical analysis was applied after log-transformation of linear data.

Supplementary Fig. S8 Chemical structure and the conversion of vinorelbine to its metabolites vinorelbine N-oxide and 4’-O-deacetylvinorelbine. The red frame indicates where carboxylesterases and CYP3A4 effectively cleave or modify vinorelbine.

Supplementary Fig. S9 Liver, kidney, lung and spleen vinorelbine concentrations (a, c, e and g), and vinorelbine tissue-to-plasma concentration ratios (b, d, f and h) in male wild-type, *Ces2^-/-^*, *Ces2^-/-^*A and *Ces2^-/-^*V mice over 4 h after oral administration of 10 mg/kg vinorelbine. Data are given as mean ± SD (*n* = 6 - 7). *, *P* < 0.05; **, *P* < 0.01; ***, *P* < 0.001 compared to wild-type mice. ^#^, *P* < 0.05; ^##^, *P* < 0.01; ^###^, *P* < 0.001 compared to *Ces2^-/-^* mice. ^, *P* < 0.05; ^^, *P* < 0.01; ^^^, *P* < 0.001 for comparison between *Ces2^-/-^*A and *Ces2^-/-^*V mice. Statistical analysis was applied after log-transformation of linear data.

Supplementary Fig. S10 Small intestine (SI), small intestine contents (SIC), small intestine contents percentage of total dose and colon vinorelbine concentrations (a, c, e and g), and vinorelbine tissue-to-plasma concentration ratios in SI, SIC, SIC (% of dose) and colon (b, d, f and h) in male wild-type, *Ces2^-/-^*, *Ces2^-/-^*A and *Ces2^-/-^*V mice over 4 h after oral administration of 10 mg/kg vinorelbine. Data are given as mean ± SD (*n* = 6 - 7). *, *P* < 0.05; **, *P* < 0.01; ***, *P* < 0.001 compared to wild-type mice. ^#^, *P* < 0.05; ^##^, *P* < 0.01; ^###^, *P* < 0.001 compared to *Ces2^-/-^* mice. ^, *P* < 0.05; ^^, *P* < 0.01; ^^^, *P* < 0.001 for comparison between *Ces2^-/-^*A and *Ces2^-/-^*V mice. Statistical analysis was applied after log-transformation of linear data.

Supplementary Fig. S11 Liver, kidney, lung and spleen deacetylvinorelbine concentrations (a, c, e and g), and deacetylvinorelbine tissue-to-plasma concentration ratios (b, d, f and h) in male wild-type, *Ces2^-/-^*, *Ces2^-/-^*A and *Ces2^-/-^*V mice over 4 h after oral administration of 10 mg/kg vinorelbine. Data are given as mean ± SD (*n* = 6 - 7). *, *P* < 0.05; **, *P* < 0.01; ***, *P* < 0.001 compared to wild-type mice. ^#^, *P* < 0.05; ^##^, *P* < 0.01; ^###^, *P* < 0.001 compared to *Ces2^-/-^* mice. ^, *P* < 0.05; ^^, *P* < 0.01; ^^^, *P* < 0.001 for comparison between *Ces2^-/-^*A and *Ces2^-/-^*V mice. Statistical analysis was applied after log-transformation of linear data.

Supplementary Fig. S12 Small intestine (SI), small intestine contents (SIC), small intestine contents percentage of total dose and colon deacetylvinorelbine concentrations (a, c, e and g), and deacetylvinorelbine tissue-to-plasma concentration ratios in SI, SIC, SIC (% of dose) and colon (b, d, f and h) in male wild-type, *Ces2^-/-^*, *Ces2^-/-^*A and *Ces2^-/-^*V mice over 4 h after oral administration of 10 mg/kg vinorelbine. Data are given as mean ± SD (*n* = 6 - 7). *, *P* < 0.05; **, *P* < 0.01; ***, *P* < 0.001 compared to wild-type mice. ^#^, *P* < 0.05; ^##^, *P* < 0.01; ^###^, *P* < 0.001 compared to *Ces2^-/-^* mice. ^, *P* < 0.05; ^^, *P* < 0.01; ^^^, *P* < 0.001 for comparison between *Ces2^-/-^*A and *Ces2^-/-^*V mice. Statistical analysis was applied after log-transformation of linear data.

Supplementary Fig. S13 Liver, kidney, lung and spleen vinorelbine concentrations (a, c, e and g), and vinorelbine tissue-to-plasma concentration ratios (b, d, f and h) in male wild-type, *Ces2^-/-^*, *Ces2^-/-^*A and *Ces2^-/-^*V mice over 4 h after I.V. injection of 10 mg/kg vinorelbine. Data are given as mean ± SD (*n* = 6 - 7). *, *P* < 0.05; **, *P* < 0.01; ***, *P* < 0.001 compared to wild-type mice. ^#^, *P* < 0.05; ^##^, *P* < 0.01; ^###^, *P* < 0.001 compared to *Ces2^-/-^* mice. ^, *P* < 0.05; ^^, *P* < 0.01; ^^^, *P* < 0.001 for comparison between *Ces2^-/-^*A and *Ces2^-/-^*V mice. Statistical analysis was applied after log-transformation of linear data.

Supplementary Fig. S14 Small intestine (SI), small intestine contents (SIC), small intestine contents percentage of total dose and colon vinorelbine concentrations (a, c, e and g), and vinorelbine tissue-to-plasma concentration ratios in SI, SIC, SIC (% of dose) and colon (b, d, f and h) in male wild-type, *Ces2^-/-^*, *Ces2^-/-^*A and *Ces2^-/-^*V mice over 4 h after I.V. injection of 10 mg/kg vinorelbine. Data are given as mean ± SD (*n* = 6 - 7). *, *P* < 0.05; **, *P* < 0.01; ***, *P* < 0.001 compared to wild-type mice. ^#^, *P* < 0.05; ^##^, *P* < 0.01; ^###^, *P* < 0.001 compared to *Ces2^-/-^* mice. ^, *P* < 0.05; ^^, *P* < 0.01; ^^^, *P* < 0.001 for comparison between *Ces2^-/-^*A and *Ces2^-/-^*V mice. Statistical analysis was applied after log-transformation of linear data.

Supplementary Fig. S15 Liver, kidney, lung and spleen deacetylvinorelbine concentrations (a, c, e and g), and deacetylvinorelbine tissue-to-plasma concentration ratios (b, d, f and h) in male wild-type, *Ces2^-/-^*, *Ces2^-/-^*A and *Ces2^-/-^*V mice over 4 h after I.V. injection of 10 mg/kg vinorelbine. Data are given as mean ± SD (*n* = 6 - 7). *, *P* < 0.05; **, *P* < 0.01; ***, *P* < 0.001 compared to wild-type mice. ^#^, *P* < 0.05; ^##^, *P* < 0.01; ^###^, *P* < 0.001 compared to *Ces2^-/-^* mice. ^, *P* < 0.05; ^^, *P* < 0.01; ^^^, *P* < 0.001 for comparison between *Ces2^-/-^*A and *Ces2^-/-^*V mice. Statistical analysis was applied after log-transformation of linear data.

Supplementary Fig. S16 Small intestine (SI), small intestine contents (SIC), small intestine contents percentage of total dose and colon deacetylvinorelbine concentrations (a, c, e and g), and deacetylvinorelbine tissue-to-plasma concentration ratios in SI, SIC, SIC (% of dose) and colon (b, d, f and h) in male wild-type, *Ces2^-/-^*, *Ces2^-/-^*A and *Ces2^-/-^*V mice over 4 h after I.V. injection of 10 mg/kg vinorelbine. Data are given as mean ± SD (*n* = 6 - 7). *, *P* < 0.05; **, *P* < 0.01; ***, *P* < 0.001 compared to wild-type mice. ^#^, *P* < 0.05; ^##^, *P* < 0.01; ^###^, *P* < 0.001 compared to *Ces2^-/-^* mice. ^, *P* < 0.05; ^^, *P* < 0.01; ^^^, *P* < 0.001 for comparison between *Ces2^-/-^*A and *Ces2^-/-^*V mice. Statistical analysis was applied after log-transformation of linear data.

Supplementary Fig. S17 Vinorelbine conversion to deacetylvinorelbine in liver and small intestine (SI). Deacetylvinorelbine-to-vinorelbine ratios in liver (a and c) and small intestine (b and d) after oral or i.v. administration, respectively, of 10 mg/kg vinorelbine. Data are given as mean ± SD (*n* = 6 - 7). *, *P* < 0.05; **, *P* < 0.01; ***, *P* < 0.001 compared to wild-type mice. ^#^, *P* < 0.05; ^##^, *P* < 0.01; ^###^, *P* < 0.001 compared to *Ces2^-/-^* mice. ^, *P* < 0.05; ^^, *P* < 0.01; ^^^, *P* < 0.001 for comparison between *Ces2^-/-^*A and *Ces2^-/-^*V mice. Statistical analysis was applied after log-transformation of linear data.

Supplementary Fig. S18 Body weight (a), liver weight (b), gonadal white adipose tissue (gWAT) weight (c), liver-to-body weight ratio (d) and gWAT-to-body weight ratio (e) of 20 weeks old female and male wild-type, *Ces2^-/-^*, *Ces2^-/-^*A and *Ces2^-/-^*V mice. Data are given as mean ± SD (*n* = 19 - 20). *, *P* < 0.05; **, *P* < 0.01; ***, *P* < 0.001 compared to wild-type mice. ^#^, *P* < 0.05; ^##^, *P* < 0.01; ^###^, *P* < 0.001 compared to *Ces2^-/-^* mice. ^, *P* < 0.05; ^^, *P* < 0.01; ^^^, *P* < 0.001 for comparison between *Ces2^-/-^*A and *Ces2^-/-^*V mice. Statistical analysis was applied after log-transformation of linear data.

Supplementary Fig. S19 Body weight and weights of different tissues and tissue-to-body weight ratios in young adult (12-weeks old) female (a and b and male (c and d) mice from wild-type, *Ces2^-/-^*, *Ces2^-/-^*A and *Ces2^-/-^*V mouse strains. IngWAT, inguinal white adipose tissue; gWAT, gonadal white adipose tissue; mWAT, mediastinal brown adipose tissue; rWAT, renal white adipose tissue; iBAT, interscapular brown adipose tissue. Data are given as mean ± SD (*n* = 9 - 16). *, *P* < 0.05; **, *P* < 0.01; ***, *P* < 0.001 compared to wild-type mice. ^#^, *P* < 0.05; ^##^, *P* < 0.01; ^###^, *P* < 0.001 compared to *Ces2^-/-^* mice. ^, *P* < 0.05; ^^, *P* < 0.01; ^^^, *P* < 0.001 for comparison between *Ces2^-/-^*A and *Ces2^-/-^*V mice. Statistical analysis was applied after log-transformation of linear data.

Supplementary Fig. S20 Plasma alkaline phosphatase (a), ALAT (b), sodium (c), potassium (d), calcium (e), chloride (f), urea (g), uric acid (h), glucose (i), HDL-Cholesterol (j), LDL-Cholesterol (k), total cholesterol (l) and triglyceride (m) concentrations in female and male wild-type, *Ces2^-/-^*, *Ces2^-/-^*A and *Ces2^-/-^*V 20-week-old mice. Data are given as mean ± SD (*n* = 15 - 21). *, *P* < 0.05; **, *P* < 0.01; ***, *P* < 0.001 compared to wild-type mice. ^#^, *P* < 0.05; ^##^, *P* < 0.01; ^###^, *P* < 0.001 compared to *Ces2^-/-^* mice. ^, *P* < 0.05; ^^, *P* < 0.01; ^^^, *P* < 0.001 for comparison between *Ces2^-/-^*A and *Ces2^-/-^*V mice. Statistical analysis was applied after log-transformation of linear data.

Supplementary Fig. S21 Hematology analysis of WBC (a), RBC (b), HGB (c), HCT (d), MCV (e), MCH (f), MCHC (g), RDW (h), RDW-SD (i), PLT (j) and MPV (k) in female and male wild-type, *Ces2^-/-^*, *Ces2^-/-^*A and *Ces2^-/-^*V 20-week-old mice. Data are given as mean ± SD (*n* = 19 - 21). WBC, white blood cells; RBC, red blood cells; HGB, hemoglobin; HCT, hematocrit (the relative volume of packed erythrocytes to whole blood); MCV, mean Corpuscular Volume (the average volume of individual erythrocytes derived from the RBC histogram, multiplied by a calibration factor); MCH, mean corpuscular hemoglobin (the weight of HGB in the average erythrocyte); MCHC, mean corpuscular hemoglobin concentration (the average weight of HGB in a measured dilution); RDW, red cell distribution width; RDW, red cell distribution width – SD; PLT, platelet count or thrombocyte count; MPV, mean platelet volume; *, *P* < 0.05; **, *P* < 0.01; ***, *P* < 0.001 compared to wild-type mice. ^#^, *P* < 0.05; ^##^, *P* < 0.01; ^###^, *P* < 0.001 compared to *Ces2^-/-^* mice. ^, *P* < 0.05; ^^, *P* < 0.01; ^^^, *P* < 0.001 for comparison between *Ces2^-/-^*A and *Ces2^-/-^*V mice. Statistical analysis was applied after log-transformation of linear data.

Supplementary Fig. S22 Lipid disposition, metabolism and homeostasis in the CES2 mouse models. Oil-red-O staining lipid droplet levels (a) and representative Oil-red-O staining images for liver lipid accumulation for each mouse strain (b) in female wild-type, *Ces2^-/-^*, *Ces2^-/-^*A and *Ces2^-/-^*V 20-week old mice (*n* = 19 - 21); Liver lipid contents in female wild-type, *Ces2^-/-^*, Ces2^-/-^A and Ces2^-/-^V 20-week old mice (*n* = 8); Plasma triglyceride basal concentration (overnight fast) before very low-density lipoprotein (VLDL) production and secretion and oral lipid tolerance test in female wild-type, *Ces2^-/-^*, Ces2^-/-^A and Ces2^-/-^V 12-week old mice (*n* = 20 - 24) (d), VLDL production and secretion (e) and oral lipid tolerance test (f) in female wild-type, *Ces2^-/-^*, *Ces2^-/-^*A and *Ces2^-/-^*V 12-week old mice (*n* = 10 - 12). Data are given as mean ± SD *, *P* < 0.05; **, *P* < 0.01; ***, *P* < 0.001 compared to wild-type mice. ^#^, *P* < 0.05; ^##^, *P* < 0.01; ^###^, *P* < 0.001 compared to *Ces2^-/-^* mice. ^, *P* < 0.05; ^^, *P* < 0.01; ^^^, *P* < 0.001 for comparison between *Ces2^-/-^*A and *Ces2^-/-^*V mice. Statistical analysis was applied after log-transformation of linear data.

Supplementary Fig. S23 Plasma triglyceride concentration above basal triglyceride concentration time curve in very low-density lipoprotein (VLDL) production and secretion test (a and b) or oral lipid tolerance test (c and d) in wild-type, *Ces2^-/-^*, *Ces2^-/-^*A and *Ces2^-/-^*V 12-week-old mice (*n* = 10 - 12). Mice were fasted overnight before the experiment. The base-line triglyceride plasma concentrations at the start of the experiment (t = 0) were subtracted from the measured triglyceride concentrations at each time point. Data are given as mean ± SD. *, *P* < 0.05; **, *P* < 0.01; ***, *P* < 0.001 compared to wild-type mice. ^#^, *P* < 0.05; ^##^, *P* < 0.01; ^###^, *P* < 0.001 compared to *Ces2^-/-^* mice. ^, *P* < 0.05; ^^, *P* < 0.01; ^^^, *P* < 0.001 for comparison between *Ces2^-/-^*A and *Ces2^-/-^*V mice. Statistical analysis was applied after log-transformation of linear data.

Supplementary Fig. S24 Glucose metabolism and homeostasis in the CES2 mouse models. The plasma concentration time curve (a), plasma glucose concentration to basal glucose concentration (before glucose administration) ratio time curve (b) and glucose AUC_0-3h_ (c) in female wild-type, *Ces2^-/-^*, *Ces2^-/-^*A and *Ces2^-/-^*V 12-week old mice over a 3 h glucose tolerance test after oral administration of 1 mg/g glucose; The plasma glucose concentration time curve (d), plasma glucose concentration to basal glucose concentration (before insulin injection) ratio time curve (e) and glucose AUC_0-3h_ (f) in female wild-type, *Ces2^-/-^*, *Ces2^-/-^*A and *Ces2^-/-^*V 16-week old mice over a 3-h insulin tolerance test after i.p. injection of 0.5 U/kg insulin. Data are given as mean ± SD (*n* = 15 - 16). *, *P* < 0.05; **, *P* < 0.01; ***, *P* < 0.001 compared to wild-type mice. ^#^, *P* < 0.05; ^##^, *P* < 0.01; ^###^, *P* < 0.001 compared to *Ces2^-/-^* mice. ^, *P* < 0.05; ^^, *P* < 0.01; ^^^, *P* < 0.001 for comparison between *Ces2^-/-^*A and *Ces2^-/-^*V mice. Statistical analysis was applied after log-transformation of linear data.
